# Rotaxane Pt^II^-complexes: mechanical bonding for chemically robust luminophores and stimuli responsive behaviour†

**DOI:** 10.1039/c9sc05507j

**Published:** 2020-01-02

**Authors:** Zhihui Zhang, Graham J. Tizzard, J. A. Gareth Williams, Stephen M. Goldup

**Affiliations:** Chemistry, University of Southampton Southampton SO51 5PG UK s.goldup@soton.ac.uk; Department of Chemistry, Durham University Durham DH1 3LE UK j.a.g.williams@durham.ac.uk

## Abstract

We report an approach to rotaxanes in which the metal ion of a cyclometallated Pt^II^ luminophore is embedded in the space created by the mechanical bond. Our results show that the interlocked ligand environment stabilises a normally labile Pt^II^–triazole bond against displacement by competing ligands and that the crowded environment of the mechanical bond retards oxidation of the Pt^II^ centre, without perturbing the photophysical properties of the complex. When an additional pyridyl binding site is included in the axle, the luminescence of the Pt^II^ centre is quenched, an effect that can be selectively reversed by the binding of Ag^I^. Our results suggest that readily available interlocked metal-based phosphors can be designed to be stimuli responsive and have advantages as stabilised triplet harvesting dopants for device applications.

## Abstract 2

We report an approach to interlocked Pt^II^ luminophores in which the mechanical bond stabilises the coordination environment of the embedded metal ion.

## Graphical abstract

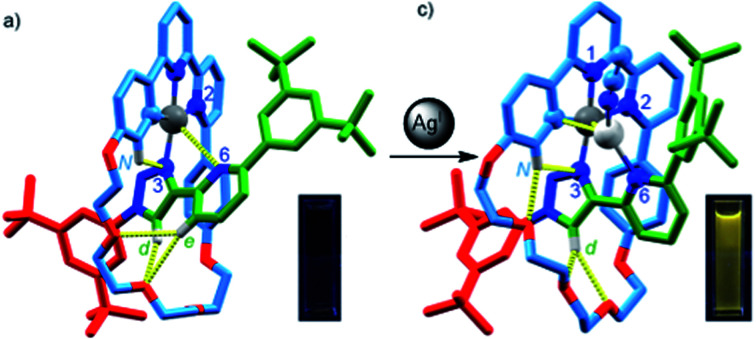

## Introduction

Many mechanically chelated^1^ metal complexes, in which a metal ion bridges mechanically bonded covalent sub-components,^2^ have been synthesised since Sauvage's first report of the Cu^I^ templated synthesis of catenanes.^3^ These complexes are typically reported as intermediates in the synthesis of interlocked molecules,^4^ as opposed to being objects of study in their own right.^5,6^ This is despite the potential benefits that embedding the metal ion within the crowded environment of the mechanical bond might bring in terms of the kinetic stabilisation and electronic, magnetic and catalytic properties. Indeed, Sauvage and co-workers highlighted the kinetic stabilisation of a bound Cu^I^ ion due to a “catenand effect”.^3,7^ Recently, we demonstrated that the mechanical bond allows access to complexes of which the non-interlocked equivalents are inaccessible,^8^ including examples with highly distorted coordination geometries reminiscent of the entatic state of enzyme active sites.^9,10^ These reports suggest that interlocked metal complexes in which the mechanical bond alters the stability or properties of the ion are an untapped resource in the development of coordination complexes for a range of applications, including catalysis, metallo-pharmaceuticals and light harvesting.

Metal-based luminophores have been at the forefront of many recent developments in light-emitting organic electronic materials for various applications.^10,11^ Iridium(iii)-based phosphors are now widely incorporated into commercial organic light-emitting devices (OLEDs) to allow the harvesting of triplet excitons that would otherwise be wasted.^12^ The strong spin–orbit coupling associated with the heavy metal ion promotes the T_1_ → S_0_ phosphorescence process, which is strongly forbidden in purely organic materials.^13^ Complexes of other 2^nd^ and 3^rd^ row metal ions are also widely investigated in this context, particularly those of platinum(ii).^14^

To date, only a limited number of mechanically chelated heavy-metal complexes have been reported. This is perhaps not surprising as most metal-directed passive template syntheses rely on early transition metals,^5^ due to their more favourable ligand exchange kinetics. Furthermore, the small number of passive templates based on Pd^II^,^15^ Ru^II^,^16^ and Au^I^ (ref.17) reported are not luminescent.^18,19^ Thus, the limited examples of interlocked molecules containing metal-based luminophores incorporate the metal complex as a substituent of the interlocked scaffold, or as a structural unit. In 2012, Terao and co-workers demonstrated that emissive Pt^II^–acetylide units embedded in the axle of a rotaxane were insulated from the local environment, leading to no change in emission between solution and solid state.^20^ More recently, Ma and co-workers demonstrated that the emission of a Pt^II^–porphyrin complex appended to a rotaxane could be modulated by mechanical motion.^21^

Here we describe the synthesis and properties of rotaxane-based Pt^II^ complexes in which the metal ion is embedded within the mechanical bond. We show that the mechanical bond does not significantly perturb the photophysical properties of the metal ion but, pleasingly, the chemical stability of the complexes is greatly enhanced relative to non-interlocked analogues. Furthermore, while studying this stabilising influence, we serendipitously identified a metallorotaxane in which the phosphorescence shows a dramatic and selective enhancement in the presence of Ag^I^, through the inhibition of a quenching pathway arising from a pyridyl unit incorporated in the structure.

## Results and discussion

### Synthesis of mechanically chelated Pt^II^ complexes

**Scheme 1:**
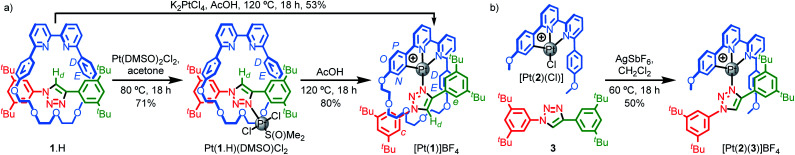
(a) Synthesis of complexes [Pt(**1**)]BF_4_. (b) Synthesis of complex [Pt(**2**)(**3**)]BF_4_.

Our study was in part inspired by a cyclometallated catenane-based Pd^II^ complex reported in early work by Sauvage and co-workers.^22^ Rotaxane **1**.H (Scheme 1) was synthesised in excellent yield from readily available starting materials^23^ using our small macrocycle modification^24^ of the active template^25^ Cu-mediated alkyne–azide cycloaddition reaction (AT–CuAAC)^26^ (see ESI† for details). Treatment of **1**.H with PtCl_2_(DMSO)_2_ under mild conditions failed to give the target cyclometallated complex [Pt(**1**)]^+^ directly, but led instead to *trans*-Pt(**1**.H)(DMSO)Cl_2_, in which the Pt centre is coordinated only by the triazole of the rotaxane axle (Scheme 1a). Subsequent heating of Pt(**1**.H)(DMSO)Cl_2_ in AcOH led to [Pt(**1**)]BF_4_ in good yield after purification.^27^ Using these more forcing conditions, it was also possible to obtain this complex directly from **1**.H and K_2_PtCl_4_ in reasonable yield. The formation of the corresponding non-interlocked complex [Pt(**2**)(**3**)]BF_4_ required a two-step procedure involving initial formation of Pt(**2**)Cl followed by Ag^I^-mediated substitution of the chloride ligand by the triazole (Scheme 1b).^28^

All novel compounds and intermediates were characterised by NMR spectroscopy and MS (see ESI†). The ^1^H NMR spectrum of [Pt(**1**)]BF_4_ clearly shows the expected desymmetrisation of the metallated aromatic ring; individual signals are observed for H_N_, H_O_ and H_P_, the first of which is significantly shielded (5.03 ppm). Perhaps counterintuitively, coordination of Pt to the triazole N leads to an upfield shift of H_d_ as metal coordination interrupts a non-classical H-bonding interaction with the bipyridine Ns. Unusually, whereas H_D_ and H_E_ each appear as 4H doublets in the ^1^H NMR spectra of **1**.H and Pt(**1**.H)(DMSO)Cl_2_, indicating free rotation of the flanking aromatic ring, H_D_ and H_E_ of [Pt(**1**)]BF_4_ each appear as two 1H doublets suggesting this motion is slow on the ^1^H NMR timescale, presumably because of the crowded nature of the macrocycle cavity with the large Pt^II^ ion included.

**Fig. 1:**
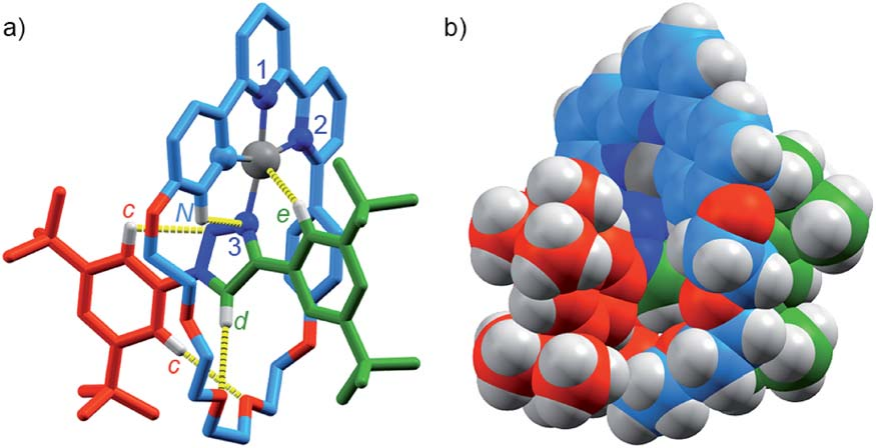
Solid-state structure of rotaxane [Pt(**1**)]BF_4_ displayed in (a) partial sticks (rotaxane framework)/ball-and-stick (metal and ligand sphere) representation (anions and majority of hydrogen atoms omitted for clarity) and (b) space-filling representation (anions omitted for clarity). Colours: H white, C as in Scheme 1, N dark blue, O red, Pt dark grey. Selected bond lengths (Å) and angles (°): Pt–C 1.99, Pt–N1 1.97, Pt1–N2 2.17, Pt1–N3 2.05, H_c_–N 2.49, H_c_–O 2.59, H_d_–O 2.53, H_e_–Pt 2.74, H_N_–N 2.56, C–Pt–N2 160.4, N1–Pt–N3 171.7, C–Pt–N1 81.9, N1–Pt–N2 78.5, N2–Pt–N3 108.4, N3–Pt–C 90.8.

Pt(**1**.H)(DMSO)Cl_2_ (Table S1†) and [Pt(**1**)]BF_4_ (Fig. 1) were further characterised in the solid-state by single-crystal X-ray diffraction (SCXRD). The solid-state structure of [Pt(**1**)]BF_4_ clearly demonstrates the mechanically chelating environment of the metal ion which bridges N^3^ of the triazole axle and the N^N^C chelate provided by the macrocycle. As is typically observed with small, sterically crowded interlocked molecules,^24^ several short intercomponent contacts are present in addition to metal–ligand interactions (Fig. 1a), including a CH⋯Pt contact. Viewing the solid-state structure in space-filling representation (Fig. 1b) underlines the sterically crowded nature of the mechanical bond; the metal ion is embedded in the cavity of the ring and largely isolated from the local environment.

### Photophysical properties of metallorotaxane [Pt(**1**)]BF_4_ and [Pt(**2**)(**3**)]BF_4_

**Fig. 2:**
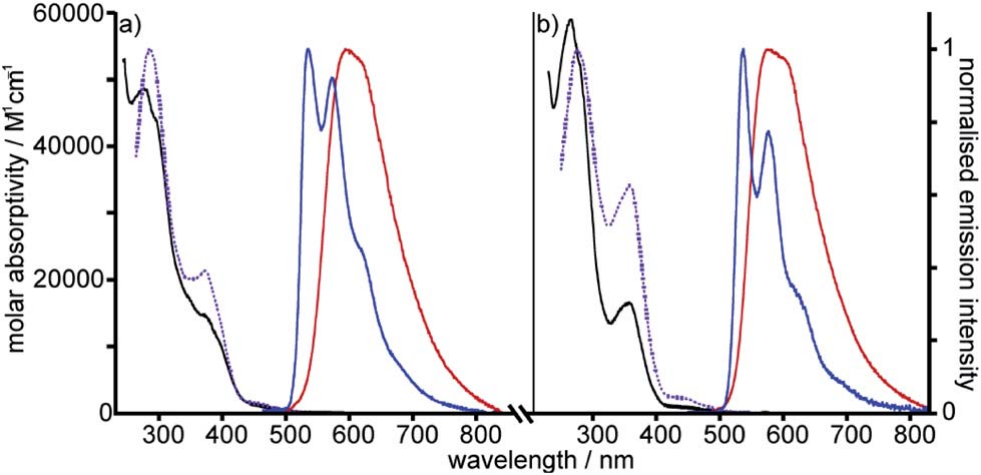
Absorption and excitation spectra in CH_2_Cl_2_ at 298 K (black and dotted purple lines respectively), and emission spectra in CH_2_Cl_2_ at 298 K and in EPA at 77 K (red and blue lines respectively) for (a) Pt[(**1**)]BF_4_ and (b) [Pt(**2**)(**3**)]BF_4_. (EPA is defined in Table 1).

The photophysical properties of the interlocked Pt^II^ complex [Pt(**1**)]BF_4_ and its non-interlocked analogue, [Pt(**2**)(**3**)]BF_4_, are remarkably similar (Table 1 and Fig. 2), demonstrating that the mechanical bond does not significantly perturb the pertinent electronic excited states. Both complexes display UV-visible absorption properties typical of cyclometallated Pt^II^ complexes of arylpyridine ligands,^29^ with intense intra-ligand bands in the far-UV and a somewhat less intense band at lower energy (350–380 nm). Bands in the latter region are typically associated with charge–transfer transitions of mixed 
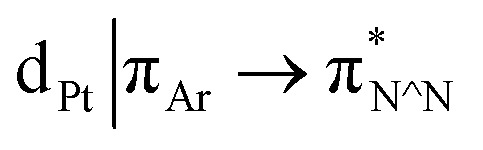
 orbital parentage, that are introduced upon cyclometallation. Notably, there is no such band for the non-cyclometallated Pt(**1**.H)(DMSO)Cl_2_ complex (Fig. S59†).

**Table 1:** Photophysical properties of [Pt(**1**)]BF_4_ and [Pt(**2**)(**3**)]BF_4_

	[Pt(**1**)]BF_4_	[Pt(**2**)(**3**)]BF_4_
**298 K in deoxygenated CH**_**2**_ **Cl**_**2**_		
*λ*_abs max_/nm (*ε*/M^−1^ cm^−1^)	264 (58 900), 358 (16 400), 441 sh (946)	262 (48 700), 356 (14 700), 448 sh (1120)
*λ*_em max_/nm	575	582
*Φ*_lum_ × 10^2^^a^	2.7	1.9
*τ*/μs	2.1 [0.46]^b^	2.2 [0.39]^b^
*k*_r_/10^3^ s^−1^^c^	13	8.6
Σ*k*_nr_/10^5^ s^−1^^c^	4.6	4.5

**77 K in frozen EPA (2 : 2** **:** **1 v/v Et**_**2**_**O–**^***i***^**C**_**5**_**H**_**12**_**–EtOH)**		
*λ*_em max_/nm	536, 576, 623	521, 559, 603
*τ*/μs	15.9	19

a Quantum yield in deoxygenated solution measured using [Ru(bpy)_3_]Cl_2(aq)_ as the standard.

b Luminescence lifetimes in deoxygenated solution; values in parenthesis refer to air-equilibrated solution.

c Radiative *k*_r_ and non-radiative Σ*k*_nr_ rate constants estimated using the approximation that the emissive state is formed with unitary efficiency and thus *k*_r_ = *Φ*/*τ* and Σ*k*_nr_ = (1 − *Φ*)/*τ*.^32^

Excitation of both cyclometallated complexes at 360 nm in degassed solution leads to an emission band centred around 575 nm. The corresponding luminescence lifetimes of around 2 μs in deoxygenated solution are indicative of a formally spin-forbidden phosphorescence process, promoted by the spin–orbit coupling associated with the metal ion. Such phosphorescence is typical of Pt(ii) complexes of 6-phenylbipyridine-based ligands, of the form Pt(N^N^C)X or [Pt(N^N^C)L]^+^, and assigned to a 
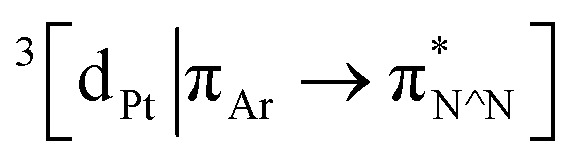
 state.^30^ The measured quantum yields of around 2–3% under these conditions are also quite typical of such complexes. The phosphorescence is modestly quenched by dissolved molecular oxygen, as evident from the shorter lifetimes observed in air-equilibrated solution. At 77 K, vibrational structure becomes clearly resolved, with a progression of around 1300 cm^−1^, quite typical of complexes featuring aromatic ligands. The complexes are also luminescent in the solid state, with similar quantum yields and lifetimes (Table S8†).^31^

### Chemical stability of interlocked Pt^II^ complexes

**Scheme 2:**
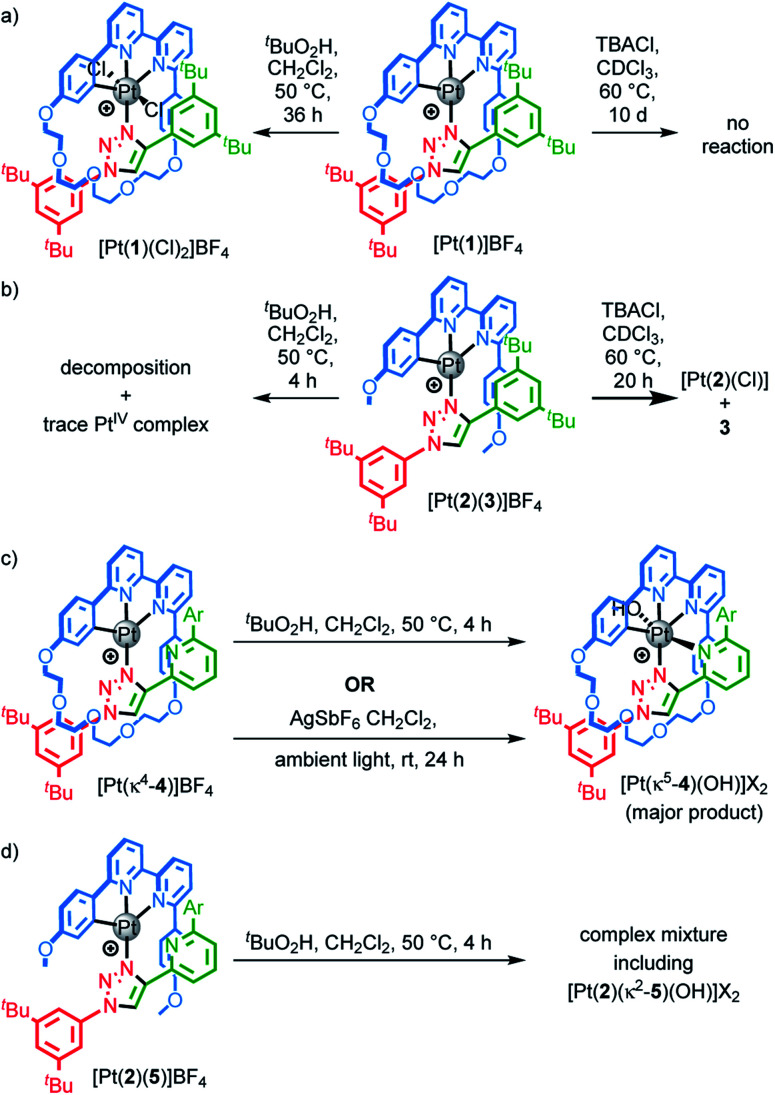
Reactions of (a) [Pt(**1**)]BF_4_, (b) [Pt(**2**)(**3**)]BF_4_, (c) [Pt(κ^4^-**4**)]BF_4_ and (d) [Pt(**2**)(**5**)]BF_4_. Ar = 3,5-di-^*t*^Bu-C_6_H_3_.

Having confirmed that metallorotaxane [Pt(**1**)]BF_4_ retains the emissive properties typical of Pt(N^N^C) complexes, we turned to the effect of the mechanical bond on the stability of the metal complex. Triazole ligands are only weakly coordinating^33^ and indeed, treatment of the non-interlocked complex [Pt(**2**)(**3**)]BF_4_ with NBu_4_Cl led to decomposition to produce [Pt(**2**)Cl] and axle **3** through exchange of the monodentate ligand (Scheme 2). In contrast, the interlocked analogue [Pt(**1**)]BF_4_ is stable over at least 5 days under the same conditions. Similarly, treatment of [Pt(**2**)(**3**)]BF_4_ with ^*t*^BuO_2_H led to rapid decomposition to a complex mixture of products (Scheme 2). The same experiment with [Pt(**1**)]BF_4_ led to a slow reaction to give an oxidised Pt^IV^ complex as the major product, which was identified by ESI-MS as [Pt(**1**)Cl_2_]BF_4_. The Cl ligands in this compound are presumably provided through oxidative decomposition of CH_2_Cl_2_ over the extended reaction time (Scheme 2).

These results suggest that the mechanical bond significantly retards displacement of the weakly coordinating triazole ligand, presumably due to steric repulsion of the incoming Cl nucleophile and destabilisation of the expected 5-coordinate intermediate of the presumed associative pathway. The same steric hindrance would also thermodynamically destabilise the product of the reaction with [Pt(**1**)]BF_4_ as the macrocycle cannot readily accommodate both a Pt–Cl moiety *and* the uncoordinated axle. The mechanical bond also appears to kinetically disfavour oxidation of the Pt^II^ centre, presumably for similar reasons, as the change from Pt^II^ to Pt^IV^ requires the inclusion of two additional ligands within the metal primary coordination sphere.

To examine if the latter effect could be overcome by providing an additional donor atom in the rotaxane framework, we synthesised complex [Pt(κ^4^-**4**)]BF_4_ (see ESI†) and examined its stability to oxidation. As predicted, when [Pt^II^(κ^4^-**4**)]BF_4_ was treated with ^*t*^BuO_2_H, ^1^H NMR analysis (Fig. S55†) revealed that a rapid reaction (<2 h) took place to give a mixture in which the species assigned as [Pt^IV^(κ^5^-**4**)(OH)](BF_4_)_2_ (*m*/*z* = 616.3 corresponding to M^2+^) was the major product. Replacing ^*t*^BuO_2_H with H_2_O_2_ led to a cleaner reaction (Fig. S56†) in which the species assigned as [Pt^IV^(κ^5^-**4**)(OH)](BF_4_)_2_ was the only significant product. This assignment was supported by SCXRD analysis of crystals grown from the crude product mixture which, although of low quality, confirmed the expected connectivity (Table S5†).

**Fig. 3:**
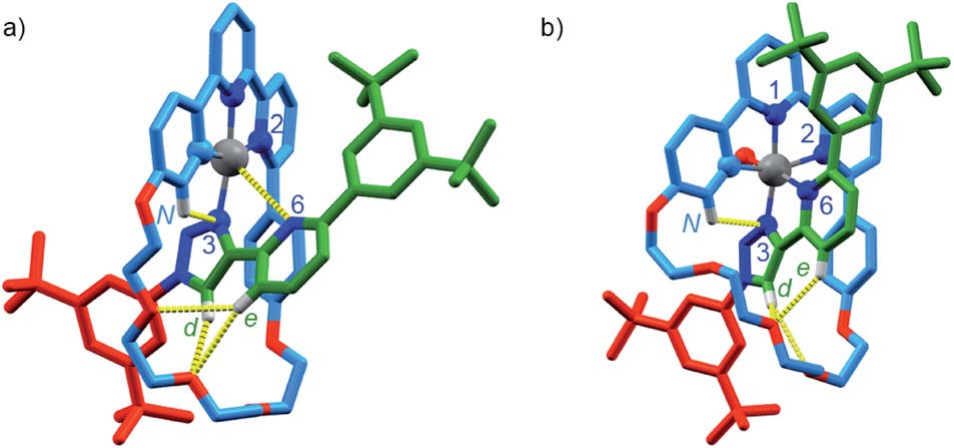
Solid-state structures of (a) [Pt(κ^4^-**4**)]BF_4_ and (b) [Pt(κ^5^-**4**)(OH)](SbF_6_)_2_ in partial sticks (rotaxane framework)/ball-and-stick (metals and ligand sphere) representation (anions and majority of hydrogen atoms omitted for clarity). Colours: H white, C as in Scheme 2, N dark blue, O red, Pt dark grey. [Pt(κ^4^-**4**)]BF_4_ selected distances (Å) and angles (°): Pt–C 1.97, Pt–N1 1.95, Pt–N2 2.17, Pt–N3 2.03, Pt–N6 3.39, H_d_–O 2.25, H_e_–O 2.79, H_e_–O 2.80, H_N_–N 2.55, C–Pt–N2 160.7, N1–Pt–N3 172.0, C–Pt–N1 82.1, N1–Pt–N2 78.8, N2–Pt–N3 107.3, N3–Pt–C 91.6. [Pt(κ^4^-**4**)(OH)](SbF_6_)_2_ selected distances (Å) and angles (°): Pt–C 2.01, Pt–N1 1.99, Pt–N2 2.22, Pt–N3 2.01, Pt–N6 2.13, Pt–O 1.97, H_d_–O 2.49, H_d_–O 2.76, H_e_–O 2.55, H_N_–N3 2.54, C–Pt–N2 160.3, N1–Pt–N3 172.6, N6–Pt–O 171.6, C–Pt–N1 82.4, N1–Pt–N2 77.9, N2–Pt–N3 108.2, N3–Pt–C 91.4.

Serendipitously (*vide infra*), when [Pt(κ^4^-**4**)]BF_4_ was treated with AgSbF_6_ without protection from light (Scheme 2c), higher quality crystals were produced and found to correspond to [Pt(κ^5^-**4**)(OH)](SbF_6_)_2_ in which the same coordination environment was also observed (Fig. 3b). The structures of [Pt(κ^4^-**4**)]BF_4_ and [Pt(κ^5^-**4**)(OH)](SbF_6_)_2_ (Fig. 3) clearly demonstrate the change from a square-planar Pt^II^ unit to a pseudo-octahedral Pt^IV^ complex in which the rotaxane framework provides five of the six donor atoms required to complete the Pt^IV^ coordination sphere (three from the macrocycle and now *two* from the axle).

The corresponding non-interlocked complex, [Pt(**2**)(**5**)]BF_4_, also reacted rapidly with ^*t*^BuO_2_H to give a mixture of products (Fig. S57†), including uncoordinated axle **5**, and a species that was assigned as Pt^IV^ complex by ESI-MS analysis (*m*/*z* = 551.2 corresponding to M^2+^). Replacing ^*t*^BuO_2_H with H_2_O_2_ simplified the product mixture at short reaction times (Fig. S58†). However, whereas [Pt^IV^(κ^5^-**4**)(OH)](BF_4_)_2_ proved stable over extended reaction times, treatment of [Pt(**2**)(**5**)]BF_4_ with H_2_O_2_ for 24 h led to decomposition to produce significant quantities of non-interlocked axle (Fig. S58†), suggesting that even in the Pt^IV^ oxidation state, the Pt–triazole bond remains labile.

### Photophysical properties of [Pt^II^(κ^4^-**4**)]BF_4_ – response to Ag^I^

The pyridine appended rotaxane complex [Pt(κ^4^-**4**)]BF_4_ displays a similar phosphorescence spectrum to that of [Pt(**1**)]BF_4_. However, its emission quantum yield in solution is dramatically reduced by around 20-fold, and the phosphorescence lifetime is an order of magnitude shorter (Table 2*vs.*Table 1). A similar difference was observed between the corresponding non-interlocked complexes [Pt(**2**)(**5**)]BF_4_ and [Pt(**2**)(**3**)]BF_4_. Estimation of the radiative and non-radiative rate constants, *k*_r_ and Σ*k*_nr_ respectively, from the quantum yields and lifetimes suggests that the effect is due primarily to the introduction of an additional non-radiative decay pathway that increases *k*_nr_ by an order of magnitude when the additional pyridine ring is present. Interestingly, the quenching effect is apparently eliminated in the solid state (Table S9†), under which conditions the quantum yields and lifetimes of all of the complexes are similar to one another, suggesting that the quenching process in the pyridine-functionalised structures requires some degree of intramolecular reorganisation that is inhibited in the rigid environment of a solid matrix.^34^

**Table 2:** Photophysical properties of [Pt(κ^4^-**4**)]BF_4_ and [Pt(**2**)(**5**)]BF_4_

	[Pt(**4**)]BF_4_	[Pt(**2**)(**5**)]BF_4_
**298 K in deoxygenated CH**_**2**_ **Cl**_**2**_		
*λ*_abs max_/nm (*ε*/M^−1^ cm^−1^)	268 (48 500), 359 (12 900), 445 sh (698)	266 (47 000), 352 (12 900), 443 (1120)
*λ*_em max_/nm	591	595
*Φ*_lum_ × 10^2^^a^	0.12	0.22
*τ*/μs^b^	0.22 [0.18]	0.10 [0.07]
*k*_r_/10^3^ s^−1^^c^	5.5	22
Σ*k*_nr_/10^5^ s^−1^^c^	45	99

**298 K in deoxygenated CH**_**2**_ **Cl**_**2**_ **with Ag** ^**I**^ **(3 equiv.)**		
*λ*_em max_/nm	581	578
*Φ*_lum_ × 10^2^^a^	10	6.0
*τ*/μs^b^	4.3 [0.95]	5.4 [1.0]
*k*_r_/10^3^ s^−1^^c^	23	11
Σ*k*_nr_/10^5^ s^−1^^c^	2.1	1.7

**77 K in frozen EPA (2 : 2** **:** **1 v/v Et**_**2**_**O–**^***i***^**C**_**5**_**H**_**12**_**–EtOH)**		
*λ*_em max_/nm	534, 574, 622	529, 555
*τ*/μs	19	18

a Quantum yield in deoxygenated solution measured using [Ru(bpy)_3_]Cl_2(aq)_ as the standard.

b Luminescence lifetimes in deoxygenated solution; values in parenthesis refer to air-equilibrated solution.

c Radiative *k*_r_ and non-radiative Σ*k*_nr_ rate constants estimated using the approximation that the emissive state is formed with unitary efficiency and thus *k*_r_ = *Φ*/*τ* and Σ*k*_nr_ = (1 − *Φ*)/*τ*.

Examining the solid state structure of [Pt(κ^4^-**4**)]BF_4_, a striking feature is that the lone pair of the axle pyridine moiety is projected near to the Pt centre (3.39 Å) in the ground state. Thus, one explanation for the quenching of the emission is through exciplex formation, where a conformational rearrangement takes place to bring the pyridine lone pair closer to the Pt^II^ centre, which becomes more electrophilic in the ^3^MLCT excited state. Indeed, it has been noted that Pt^II^ complexes with “dangling” nucleophilic groups are often non-emissive (or only weakly so), ostensibly due to such axial interactions, whilst similar reasoning has been invoked to explain the frequently lower quantum yields of Pt^II^ complexes in solvents incorporating potential donor atoms like DMF.^35^ McMillin and co-workers have highlighted the correlation between the Gutmann donor number^36^ and the rate constant for intermolecular quenching of the luminescence of Pt^II^ complexes by donors such as pyridine.^37^

**Fig. 4:**
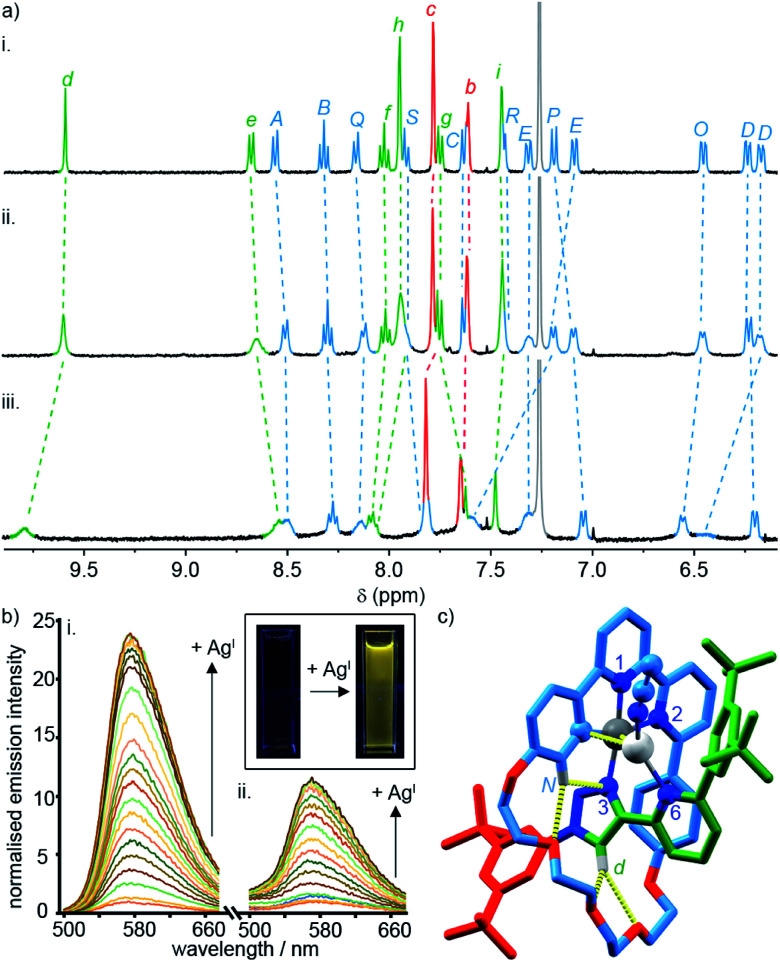
(a) Partial ^1^H NMR of (i) [Pt(κ^4^-**4**)]BF_4_, (ii) [Pt(κ^4^-**4**)]BF_4_ + 1 equiv. AgSbF_6_, (iii) [Pt(κ^4^-**4**)]BF_4_ + 1 equiv. of [Cu(MeCN)_4_]PF_6_. (b) Phosphorescence response of (i) [Pt(κ^4^-**4**)]BF_4_ and (ii) [Pt(**2**)(**5**)]BF_4_ to the portion-wise addition of AgSbF_6_ (inset: visual demonstration of the luminescent switch-on of [Pt(κ^4^-**4**)]BF_4_ in the presence of 10 equiv. of Ag^I^). (c) Solid-state structure of [Pt(κ^4^-**4**)Ag(MeCN)]BF_4_ in partial sticks (rotaxane framework)/ball-and-stick (metals and ligand sphere) representation (anions and majority of Hs omitted for clarity). Colours: H white, C as in Scheme 2, N dark blue, O red, Pt dark grey, Ag light grey. Selected distances (Å) and angles (°): Pt–C 1.98, Pt–N1 1.95, Pt–N2 2.17, Pt–N3 2.02, Ag–Pt 2.83, Ag–C 2.54, H_d_–O 2.28, H_d_–O 2.60, H_N_–N3 2.51, H_N_–O 2.59, C–Pt–N2 161.3, N1–Pt–N3 172.1, C–Pt–N1 82.2, N1–Pt–N2 79.1, N2–Pt–N3 108.4, N3–Pt–C 90.3.

To probe the role of the axle pyridine in [Pt(κ^4^-**4**)]BF_4_, we attempted to protonate it, in order to determine if its elimination as a potential donor group would enhance the luminescence. However, no change was observed by ^1^H NMR spectroscopy (Fig. S61†) when [Pt(κ^4^-**4**)]BF_4_ was treated with trifluoroacetic acid. The apparent lack of basic behaviour may be due to charge–charge repulsion between the formally cationic Pt^II^ centre and the protonated pyridine moiety that destablises the protonated state. We therefore examined instead the binding of the soft, monovalent cations Cu^I^, Ag^I^, and Au^I^ in the hope that this charge–charge repulsion might be compensated for by attractive metal–metal interactions. Pleasingly, when [Pt(κ^4^-**4**)]BF_4_ was titrated with AgSbF_6_ or [Cu(MeCN)_4_]PF_6_, significant changes were observed by ^1^H NMR (Fig. 4a), suggesting that Ag^I^ or Cu^I^ may bind to the pyridine ring of the axle. No significant changes were observed upon addition of [AuCl(SMe_2_)]. Similarly, divalent cations Zn^II^ and Cd^II^ failed to elicit any observable change by ^1^H NMR (Fig. S61†).

Titration of [Pt(κ^4^-**4**)]BF_4_ with Ag^I^ and Cu^I^ monitored by UV-vis or luminescence spectroscopy revealed significant changes with incremental addition of metal salt (Fig. S61–S70†). Most strikingly, a switch-on of the emission was observed as a result of Ag^I^ binding but not in the case of Cu^I^; in the presence of 3 equiv. of Ag^I^, the emission intensity increased by ∼25-fold at *λ*_max_ upon excitation at 400 nm (Fig. 4b). The quantum yield and lifetime of the emission increased by two orders of magnitude, and the corresponding estimated rate constants (Table 2) suggest that the dramatic effect of silver is due to the combined effect of suppressed non-radiative decay and an increase in *k*_r_. The consequent increase in luminescence is strikingly visible to the naked eye (Fig. 4b inset).

SCXRD analysis of crystals obtained from a solution of [Pt(κ^4^-**4**)]BF_4_ and AgSbF_6_ in the dark confirmed that the isolated product incorporates a Ag^I^ ion coordinated to the pyridine ring of the axle (Fig. 4c). This heterodinuclear complex appears to be stabilised both by a metal–metal interaction between the Pt^II^ and Ag^I^ centres (2.83 Å) and by an *η*^2^ π-interaction between the Ag^I^ centre and the bipyridine unit (2.54 Å), although it should be noted that in crystals grown under different conditions, only the Ag–Pt contact is maintained (Table S7†). Thus, we tentatively assign the observed increase in the luminescence quantum yield of [Pt(κ^4^-**4**)]BF_4_ in the presence of Ag^I^ to inhibition of the proposed quenching mechanism involving the pyridine-N⋯Pt interaction in the excited state, thus decreasing Σ*k*_nr_. The increase in *k*_r_, albeit a modest one, may reflect more efficient spin–orbit coupling pathways in the dinuclear system, in line with other recent reports of hetero-dinuclear^38^ and polynuclear^39^ complexes incorporating Pt^II^, although these pathways remain poorly understood.

Titration of [Pt(**2**)(**5**)]BF_4_ with Ag^I^ revealed an increase in luminescence efficiency, albeit with a lower switch-on effect (∼12 fold). The difference in behaviour between [Pt(κ^4^-**4**)]BF_4_ and the corresponding non-interlocked complex may reflect the lower rigidity of the non-interlocked system; the pyridine moiety is sterically constrained in [Pt(κ^4^-**4**)]BF_4_ to project towards the Pt^II^ centre, whereas the non-interlocked complex has more conformational freedom which may entropically disfavour the Pt^II^⋯N interaction and thus lower the efficiency of the proposed quenching mechanism.

## Conclusions

In conclusion, we have demonstrated that interlocked cyclometallated Pt^II^ complexes – which are readily synthesised using an active template strategy – retain the photophysical properties of the parent non-interlocked complexes. The mechanical bond sterically stabilises the metal centre towards ligand displacement and – when no additional donors are present in the structure – towards oxidation, suggesting that this approach may have significant benefits for the construction of more stable emitters (*e.g.*, as required in applications such as OLEDs, where stability and longevity are crucial). In particular, it allows one or more weakly coordinating ligands to be considered for the optimisation of the luminescence properties, since the chemical integrity of the luminophore is maintained by the mechanical bond rather than relying on the *intrinsic* strength of the metal–ligand interaction.^33^ In addition, we serendipitously identified a luminescent sensory system for Ag^+^ with >20 fold switch-on, in which the preorganisation provided by the mechanical bond appears to lead to enhanced performance compared with the non-interlocked analogue.

More generally, we have demonstrated that interlocked, mechanically chelated late transition metal complexes can be stabilised by taking advantage of the catenand effect, something that has previously, to our knowledge, only been observed in the case of Cu^I^.^7^ Just taking the example of platinum alone, such complexes are not only of interest for their photophysical properties but also in cancer chemotherapy – where both Pt^II^ and Pt^IV^ species are known to be effective^40,41^ – and in catalysis.^42^ Our results indicate that it may be possible to use the mechanical bond to augment the stability of key reaction intermediates or even divert the system down an alternative pathway^43^ in such chemical applications.

Finally, it is also noteworthy that all of the interlocked Pt complexes reported here are examples of mechanically planar chiral rotaxanes – systems in which the mechanical bond acts as a stereogenic unit.^44^ Indeed, ^1^H NMR analysis of [Pt(**1**)]BF_4_ in the presence of the chiral anion “trisphat” revealed the appearance of two sets of signals corresponding to the diastereomeric ion pairs of *R*_mp_-[Pt(**1**)]Δ-trisphat and *S*_mp_-[Pt(**1**)]Δ-trisphat (Fig. S75†). [Pt(κ^5^-**4**)(OH)](BF_4_)_2_ also contains a stereogenic Pt^IV^ centre, the configuration of which is dictated by the mechanical stereochemistry of the precursor. Although all of these compounds were formed as racemic mixtures in the present study, we have recently developed methodology to stereoselectively access mechanically planar chiral rotaxanes^45^ and related topologically chiral catenanes.^46^ These results add further perspectives to the potential applications of interlocked heavy metal complexes in, for example, asymmetric catalysis^47^ and circularly polarised luminescence.^48^

## Conflicts of interest

There are no conflicts to declare.

## Supplementary Material

SC-011-C9SC05507J-s001

SC-011-C9SC05507J-s002
