# Supplementary material for: Rotaxane PtII-complexes: mechanical bonding for chemically robust luminophores and stimuli responsive behaviour
Source: Chem Sci. 2020 Jan 2;11(7):1839–47. doi: 10.1039/c9sc05507j (PMC8148368; doi:10.1039/c9sc05507j)
Supplement: SC-011-C9SC05507J-s001 [file SC-011-C9SC05507J-s001.pdf]

Electronic Supplementary Material (ESI) for Chemical Science.
This journal is © The Royal Society of Chemistry 2020

Supporting Information

Rotaxane Pt^{II}-Complexes: Mechanical Bonding for Chemically Robust Luminophores and Stimuli Responsive Behaviour

Zhihui Zhang,[a] Graham J. Tizzard,[a] J. A. Gareth Williams,[b]* Stephen M. Goldup[a]*

[a] Chemistry, University of Southampton, Highfield, Southampton, SO17 1BJ, UK.
[b] Department of Chemistry, Durham University, Durham DH1 3LE, U. K.

Email: j.a.g.williams@durham.ac.uk
S.Goldup@soton.ac.uk

1 General experimental procedures 4
2 Experimental Procedures 6
2.1 Synthesis of [Pt(1H)(Cl)$_2$(DMSO)] 6
2.2 Synthesis of [Pt(1)]BF_4 10
2.3 Synthesis of 2H 14
2.4 Synthesis of [Pt(2)Cl] 17
2.5 Synthesis of [Pt(2)(3)]BF_4 20
2.6 Synthesis of 4H 23
2.7 Synthesis of [Pt(4)]BF_4 27
2.8 Synthesis of [Pt(2)(5)]BF_4 31
3 Single crystal X-ray crystallographic data 35
Pt(1H)Cl_2(DMSO) 36
3.1 36
3.2 [Pt(1)]BF_4 37
3.3 Pt(k^4-4)BF_4 38
3.4 [Pt(k^5-4)(0H)]$(SbF_6)_2$ 39
3.5 [Pt(k^5-4)OH]$(BF_4)_2$ 40
3.6 [Pt(k^4-4)Ag(CH_3CN)]$(SbF_6)_2$ 41
3.7 [Pt(k^4-4)Ag(Et_2O)]$(SbF_6)_2$ 42
4 Stability of Pt(II) complex 43
4.1 Slow decomposition of complex [Pt(2)(3)]BF_4 in $CDCl_3$ 43
4.2 Slow decomposition of complex [Pt(2)(5)]BF_4 in $CDCl_3$ 44
4.3 Ligand substitution of [Pt(2)(3)]BF_4 45
4.4 Ligand substitution of [Pt(2)(5)]BF_4 46
4.5 Ligand substitution of [Pt(1)]BF_4 47
4.6 Ligand substitution of [Pt(k^4-4)]BF_4 48
4.7 Oxidation of [Pt(1)]BF_4 with *tert*-butyl hydroperoxide 49
4.8 Oxidation of [Pt(2)(3)]BF_4 with *tert*-butyl hydroperoxide 50
4.9 Oxidation of [Pt(k^4-4)]BF_4 with *tert*-butyl hydroperoxide 51
4.10 Oxidation of [Pt(k^4-4)]BF_4 with H_2O_2 52
4.11 Oxidation of [Pt(2)(5)]BF_4 with *tert*-butyl hydroperoxide 53
4.12 Oxidation of [Pt(2)(5)]BF_4 with H_2O_2 54
5 Photophysical data 55
6 Effect of additives on [Pt(k^4-4)]BF_4 57
6.1 ^{1}H NMR spectra of complex [Pt(k^4-4)]BF_4 with 1 equivalent of H^+, Ag^I, Cu^I or Au^I 57
6.2 ^{1}H NMR Titration of complex [Pt(k^4-4)]BF_4 with $AgSbF_6$ in CD_2Cl_2 57
6.1 UV-Vis titration of complex [Pt(k^4-4)]BF_4 with $AgSbF_6$ 59
6.2 Emission titrations of complex [Pt(k^4-4)]BF_4 with $AgSbF_6$ 60
6.1 Job Plot of complex [Pt(k^4-4)]BF_4 with $AgSbF_6$ 61

6.2 Titration of [Pt(k^4-4)]BF_4 with Cu(I) ... 62
6.3 Titration of [Pt(k^4-4)]BF_4 with [Au(Cl)(Me_2S)] ... 64
7 Titration of non-interlocked Pt complex [Pt(2)(5)]BF_4 with Ag(I) ... 64
7.1 UV-vis titrations of complex [Pt(2)(5)]BF_4 with $AgSbF_6$ ... 64
8 Addition of Δ-trisphat tetrabutylammonium salt to [Pt(1)]BF_4 ... 66
9 References ... 67

1 General experimental procedures

Unless otherwise stated, all reagents were purchased from commercial sources (Sigma Aldrich, Fisher Scientific, Alfa Aesar, Acros and Fluorochem) and used without further purification. $[Cu(MeCN)_4][PF_6]$ was prepared as described by Pigorsch and Köckerling.[1] Anhydrous solvents were purchased from Acros. Experiments carried out in sealed vessels were performed in CEM microwave vials, with crimped caps, with PTFE septa. Unless otherwise stated, all reactions were carried out under an inert atmosphere of nitrogen. Flash column chromatography was performed using Biotage Isolera-4 or Isolera-1 automated chromatography system, employing Biotage SNAP or ZIP cartridges (50 µm, irregular silica, default flow rates). All azide waste was disposed of as described by Gardiner and co-workers.[2] Petrol refers to the fraction of petroleum ether boiling in the range 40-60 °C. TFA refers to trifluoroacetic acid. THF refers to tetrahydrofuran. DIPEA refers to *N,N*-diisopropylethylamine. Analytical TLC was performed on pre-coated silica gel plates on aluminum (0.25 mm thick, 60F254, Merck, Germany) and observed under UV light (254 nm). EDTA-NH_3 solution refers to an aqueous solution of NH_3 (17% w/w) saturated with sodium-ethylenediaminetetraacetate. All melting points were determined using a Griffin apparatus and are uncorrected. NMR spectra were recorded on Bruker AV400 or AV500 instrument, at a constant temperature of 298 K. Chemical shifts are reported in parts per million from low to high field and referenced to residual solvent. Coupling constants (*J*) are reported in Hertz (Hz). Standard abbreviations indicating multiplicity were used as follows: m = multiplet, quint = quintet, q = quartet, t = triplet, d = doublet, s = singlet, app. = apparent, br = broad, sept = septet. Signal assignment was carried out using 2D NMR methods (HSQC, HMBC, COSY, NOESY/ROESY, TOCSY etc) where necessary. In the case of some complex multiplets with contributions from more than proton signals, such as diastereoisomers, exact assignment was not possible. Here indicative either/or assignments (e.g. H_A or H_B) are provided. For clarity all proton signals corresponding to the axle components are in lower case, and all proton signals corresponding to the macrocycle components are in upper case. Low resolution mass spectrometry was carried out by the mass spectrometry services at University of Southampton (Waters TQD mass spectrometer equipped with a triple quadrupole analyser with UHPLC injection [BEH C_{18} column; MeCN-H_2O gradient {0.2% formic acid}]). High resolution mass spectrometry was carried out either by the mass spectrometry service at the University of Edinburgh (ThermoElectron MAT 900) or by the mass spectrometry services at the University of Southampton (MaXis, Bruker Daltonics, with a Time of Flight (TOF) analyser; samples were introduced to the mass spectrometer *via* a Dionex Ultimate 3000 autosampler and uHPLC pump in a gradient of 20% MeCN in hexane to 100% acetonitrile (0.2% formic acid) over 5-10 min at 0.6 mL/min; column: Acquity UPLC BEH C_{18} (Waters) 1.7 micron 50 × 2.1mm).

Absorption spectra in solution were measured on a Biotek Instruments XS spectrometer, using quartz cuvettes of 1 cm path length. Samples for emission measurements were contained within quartz cuvettes of 1 cm path length modified to allow connection to a high-vacuum line. Degassing was achieved *via* a minimum of three freeze-pump-thaw cycles whilst connected to the vacuum manifold; final vapour pressure at 77 K was $< 5 \times 10^{-2}$ mbar, as monitored using a Pirani gauge. Luminescence quantum yields were determined using aqueous $[Ru(bpy)_3]Cl_2$ as the standard ($\Phi_{lum} = 0.028$).[3] The luminescence lifetimes of the complexes were measured by time-correlated single photon counting (TCSPC), following excitation at 405 nm with an EPL-405 pulsed diode

laser. The emitted light was detected at 90° using a Peltier-cooled R928 PMT after passage through a monochromator. The estimated uncertainty in the quoted lifetimes is ± 10% or better. Spectra at 77 K were recorded in a glass of EPA (= diethyl ether / isopentane / ethanol, 2:2:1 v/v).

The following compounds were synthesised according to literature procedures; Macrocycle **S1**,[4] half axle **S2**,[5] 1,3-di-tert-butyl-5-ethynylbenzene (**S3**),[6] 1-azido-3,5-ditert-butylbenzene (**S4**),[7] 2-bromo-6-(4-methoxyphenyl)pyridine (**S5**),[8] **1H**,[4] axle **3**[5] and axle **5**[5] were made according to literature procedures.

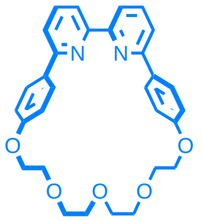

S1

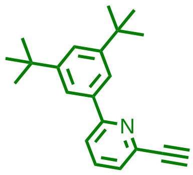

S2

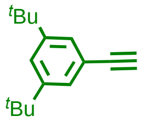

S3

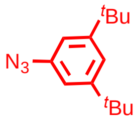

S4

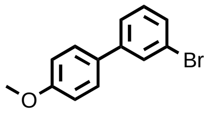

S5

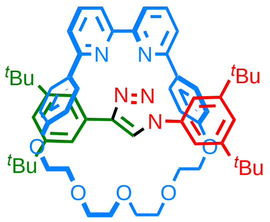

1H

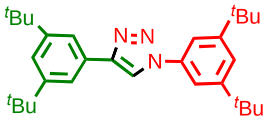

3

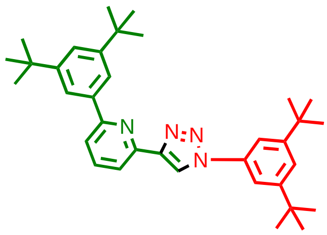

5

2 Experimental Procedures

2.1 Synthesis of [Pt(1H)(Cl)$_2$(DMSO)]

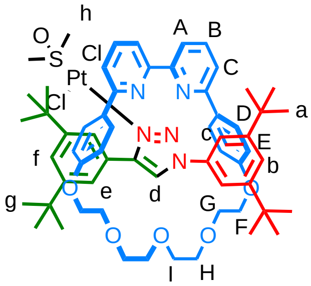

[Pt(**1**H)(Cl)$_2$(DMSO)]

Acetone (5 mL) was added to a mixture of rotaxane **1H** (0.0272 mmol, 25.8 mg, 1 eq) and Pt(DMSO)$_2$Cl$_2$ (0.0326 mmol, 13.8 mg, 1.2 eq), the colourless suspension was stirring at 80 °C for 18 h during which time a pale-yellow solution formed. After removal of solvent, the yellow mixture was purified by flash column chromatography (10 % MeCN in 1/1 CH_2Cl_2/petrol), giving the final product as off-white foam (24 mg, 71%.) ^{1}H NMR (400 MHz, $CDCl_3$) δ 10.57 (s, 1H, H$_d$), 7.80 – 7.72 (m, 4H, H$_e$ + H$_B$), 7.69 (d, *J* = 7.6 Hz, 2H, H$_A$), 7.65 – 7.58 (m, 2H, H$_c$), 7.37 (d, *J* = 7.6 Hz, 2H, H$_C$), 7.27 (s, 1H, H$_b$), 7.23 (t, *J* = 1.6 Hz, 1H, H$_f$), 7.00 (d, *J* = 8.7 Hz, 4H, H$_E$), 6.33 (d, *J* = 8.7 Hz, 4H, H$_D$), 4.40 (dt, *J* = 10.0, 6.0 Hz, 2H, 2 of H$_I$), 4.20 (dt, *J* = 11.6, 6.1 Hz, 2H, 2 of H$_F$), 4.04 (dt, *J* = 11.2, 5.8 Hz, 2H, 2 of H$_H$), 3.88 – 3.77 (m, 4H, 2 of H$_F$ + 2 of H$_I$), 3.69 (m, 4H, 2 of H$_G$ + 2 of H$_H$), 3.56 (dt, *J* = 11.1, 5.8 Hz, 2H, 2 of H$_G$), 3.31 (s, 6H, H$_h$), 1.14 (d, *J* = 3.7 Hz, 18H, H$_g$), 1.09 (s, 18H, H$_a$). ^{13}C NMR (101 MHz, $CDCl_3$) δ 160.3, 159.0, 157.0, 151.9, 150.4, 147.7, 137.5, 135.8, 132.6, 128.8, 127.6, 123.3, 123.0, 122.6, 120.6, 119.5, 114.6, 114.5, 70.6, 70.3, 69.0, 67.2, 43.6, 35.2, 34.9, 31.5, 31.2. HR-MS-ESI: m/z (found) = 1288.5, m/z calc. = 1288.5 [M]$^+$.

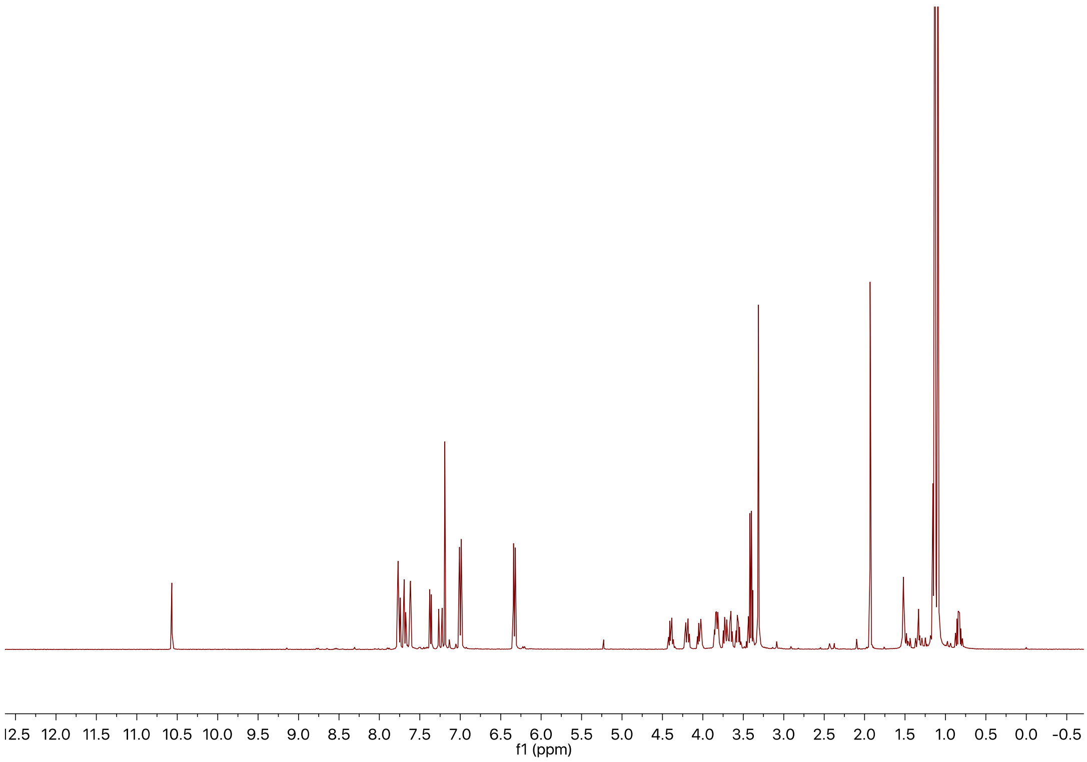

Figure S1. ^{1}H NMR ($CDCl_3$, 400 MHz) of [Pt(**1**H)(Cl)$_2$(DMSO)

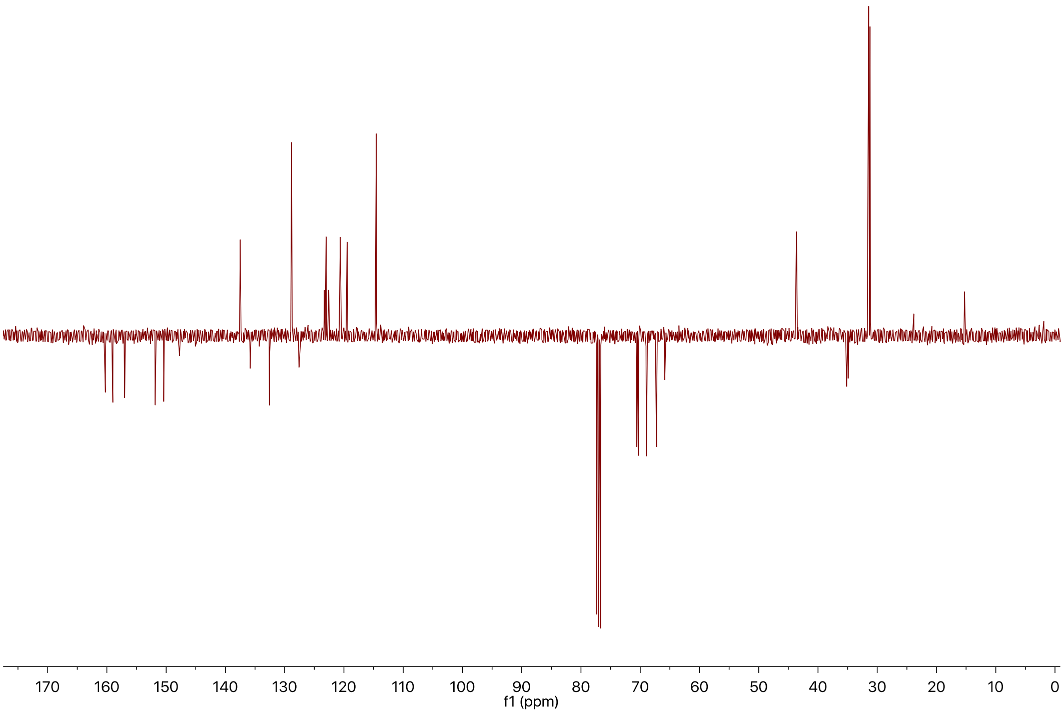

Figure S2. ^{13}C NMR ($CDCl_3$, 101 MHz) of [Pt(**1**H)(Cl)$_2$(DMSO)]

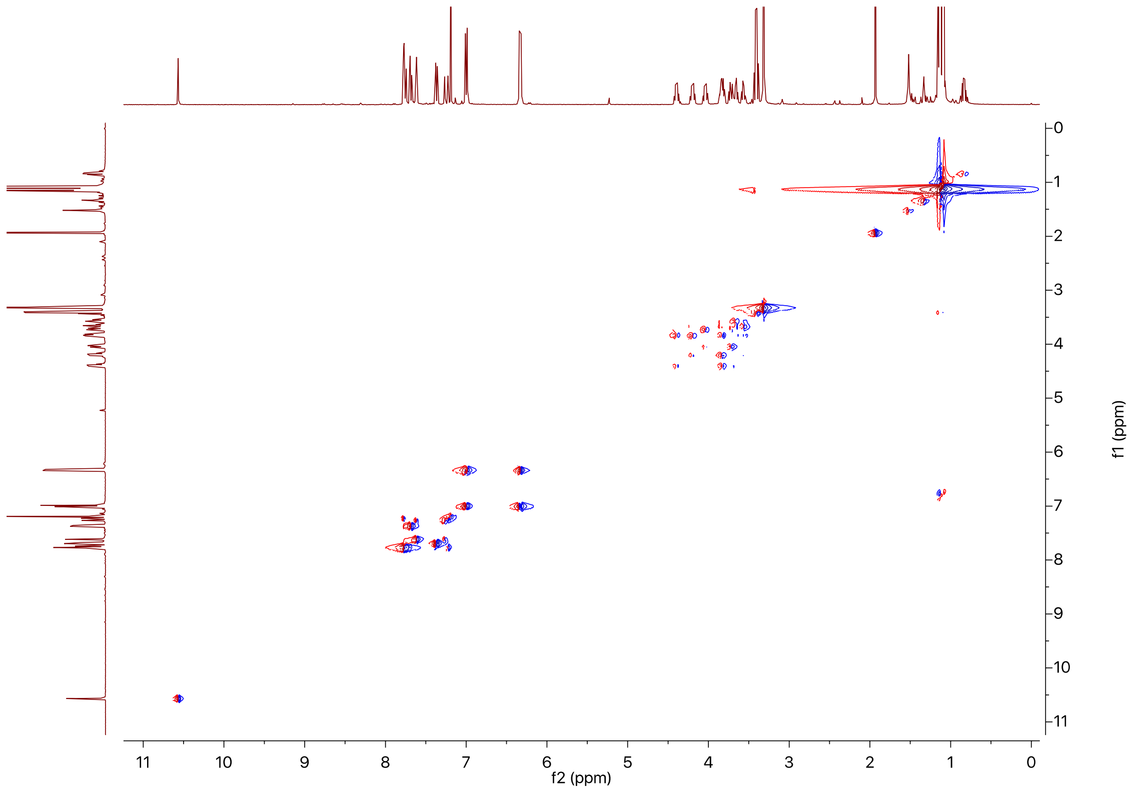

Figure S3. COSY NMR ($CDCl_3$) of [Pt(**1**H)(Cl)$_2$(DMSO)]

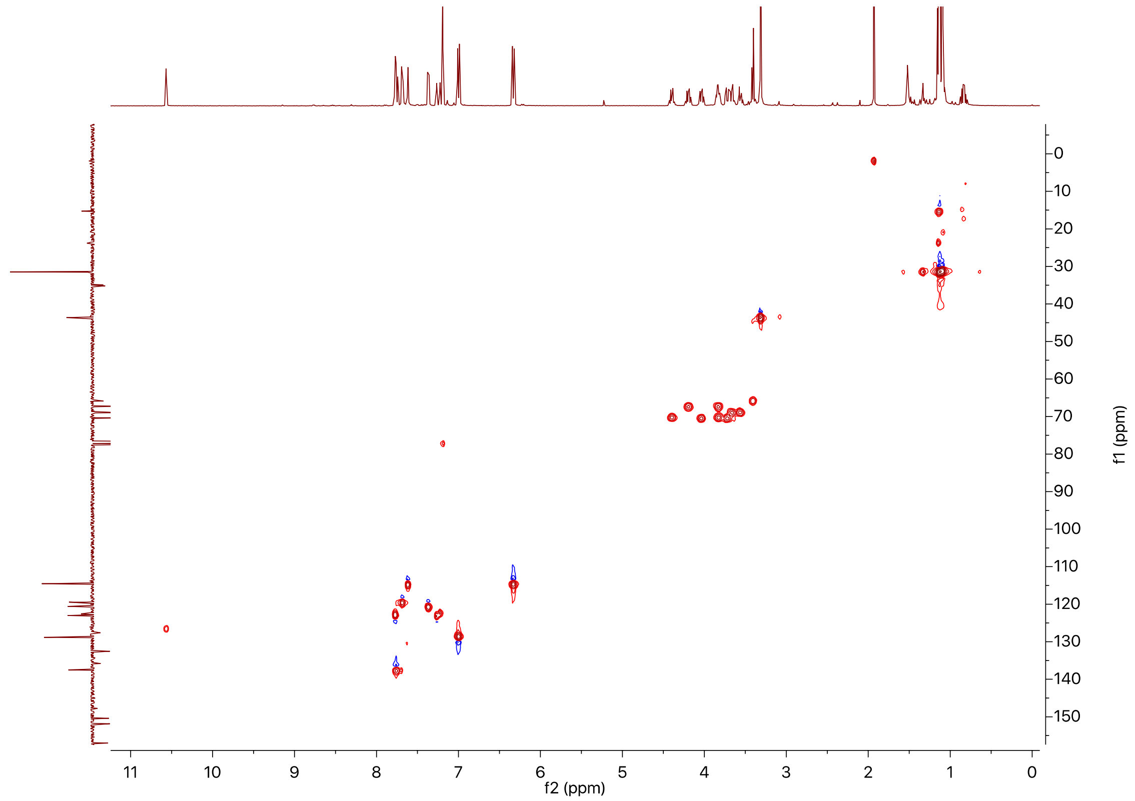

Figure S4. HSQC NMR ($CDCl_3$) of [Pt(**1**H)(Cl)$_2$(DMSO)]

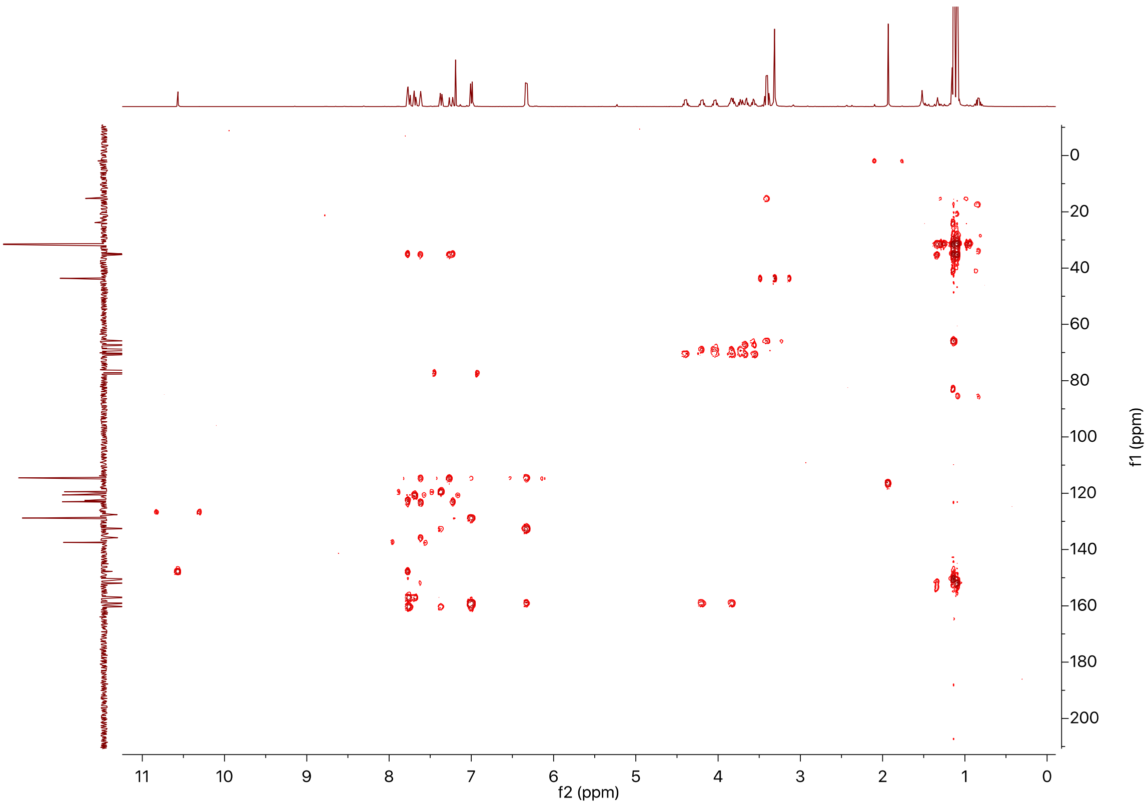

Figure S5. HMBC NMR ($CDCl_3$) of [Pt(**1**H)(Cl)$_2$(DMSO)]

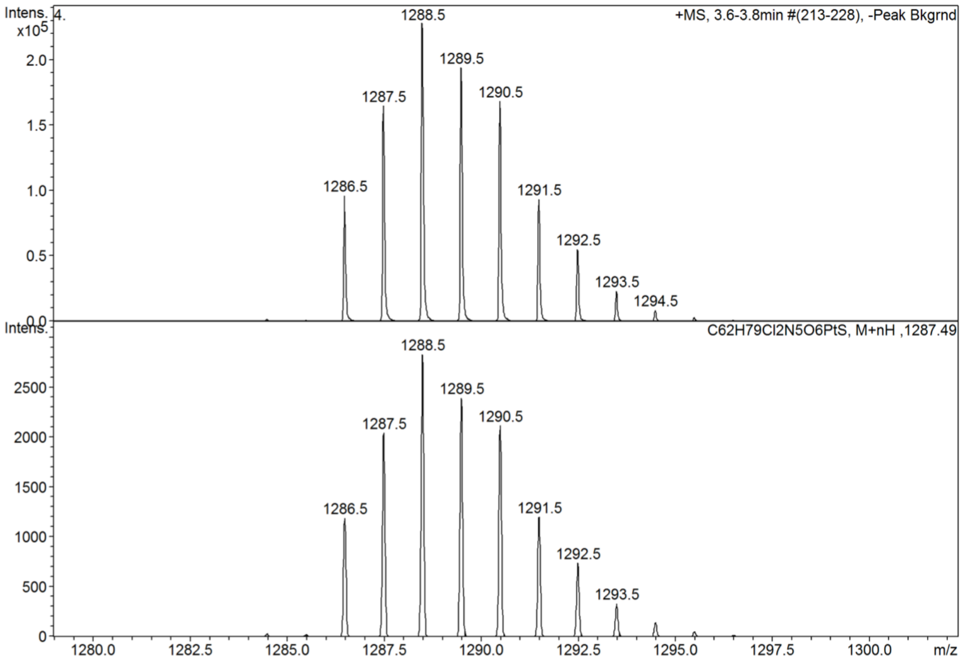

Figure S6. Isotope pattern of [Pt(**1**H)(Cl)$_2$(DMSO)]

2.2 Synthesis of [Pt(1)]BF_4

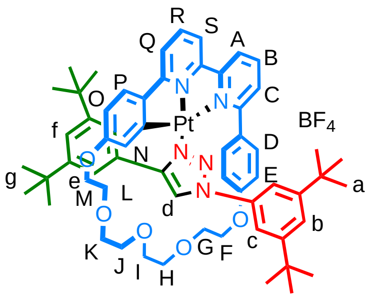

[Pt(**1**)]BF_4

A mixture of **1H** (38 mg, 0.040 mmol, 1 eq), K_2PtCl_4 (22 mg, 0.054 mmol, 1.5 eq) in glacial acetic acid (1 mL) was stirred at 120 ºC overnight. The yellow mixture was washed with saturated NH_4BF_4 solution (2 × 30 mL) and aqueous phase was extracted with CH_2Cl_2 (2 × 15 mL). The organic phase was dried over $MgSO_4$ and the solvent was evaporated *in vacuo*. The yellow mixture was purified by flash column chromatography (0 to 7% MeOH in CH_2Cl_2), affording bright yellow solid (26 mg, 53%). ^{1}H NMR (500 MHz, $CDCl_3$) δ 9.41 (s, 1H, H_d), 8.64 (dd, J = 8.1, 1.0 Hz, 2H, H_A), 8.57 (d, J = 1.5 Hz, 2H, H_c), 8.36 (t, J = 7.9 Hz, 1H, H_B), 8.25 (d, J = 7.6 Hz, 1H, H_S), 8.07 (t, J = 8.1 Hz, 1H, H_R), 7.78 (d, J = 1.7 Hz, 2H, H_e), 7.67 – 7.60 (m, 2H, H_C + H_f), 7.48 (d, J = 7.7 Hz, 1H, H_Q), 7.39 (t, J = 1.7 Hz, 1H, H_b), 7.21 (d, J = 8.6 Hz, 1H, H_P), 7.18 (dd, J = 8.4, 2.3 Hz, 1H, one of H_D), 6.93 (dd, J = 8.4, 2.3 Hz, 1H, one of H_D), 6.46 (dd, J = 8.5, 2.5 Hz, 1H, H_O), 6.28 (dd, J = 8.4, 2.6 Hz, 1H, one of H_E), 6.20 (dd, J = 8.5, 2.6 Hz, 1H, one of H_E), 5.03 (d, J = 2.5 Hz, 1H, H_N), 3.99 – 3.91 (m, 1H, 1 of H_F), 3.86 (m, 2H, 1 of H_F, 1 of H_G or H_H), 3.81-3.67 (m, 6H, 1 of H_M, 2 of H_G + H_H or 2 of H_H + H_G, H_I), 3.64 – 3.48 (m, 3H, 1 of H_M + H_J), 3.28 (ddd, J = 10.3, 6.1, 2.0 Hz, 1H, 1 of H_K), 3.22 – 3.05 (m, 3H, 1 of H_K + H_L), 1.43 (s, 18H, H_a), 1.24 (s, 18H, H_g). ^{13}C NMR (126 MHz, $CDCl_3$) δ 165.4, 162.9, 160.1, 158.7, 157.1, 155.9, 153.3, 151.2, 148.1, 141.8, 141.7, 139.6, 138.2, 136.2, 131.2, 129.8, 129.6, 128.8, 126.3, 126.1, 124.2, 123.8, 123.7, 122.4, 119.6, 118.3, 116.2, 114.8, 114.0, 112.9, 112.4, 71.2, 71.0, 70.8, 70.7, 69.6, 67.1(2), 66.0, 35.4, 35.2, 31.4, 31.2. HR-MS-ESI: m/z (found) = 1138.5, m/z calc. = 1139.0 [M-BF_4]$^+$.

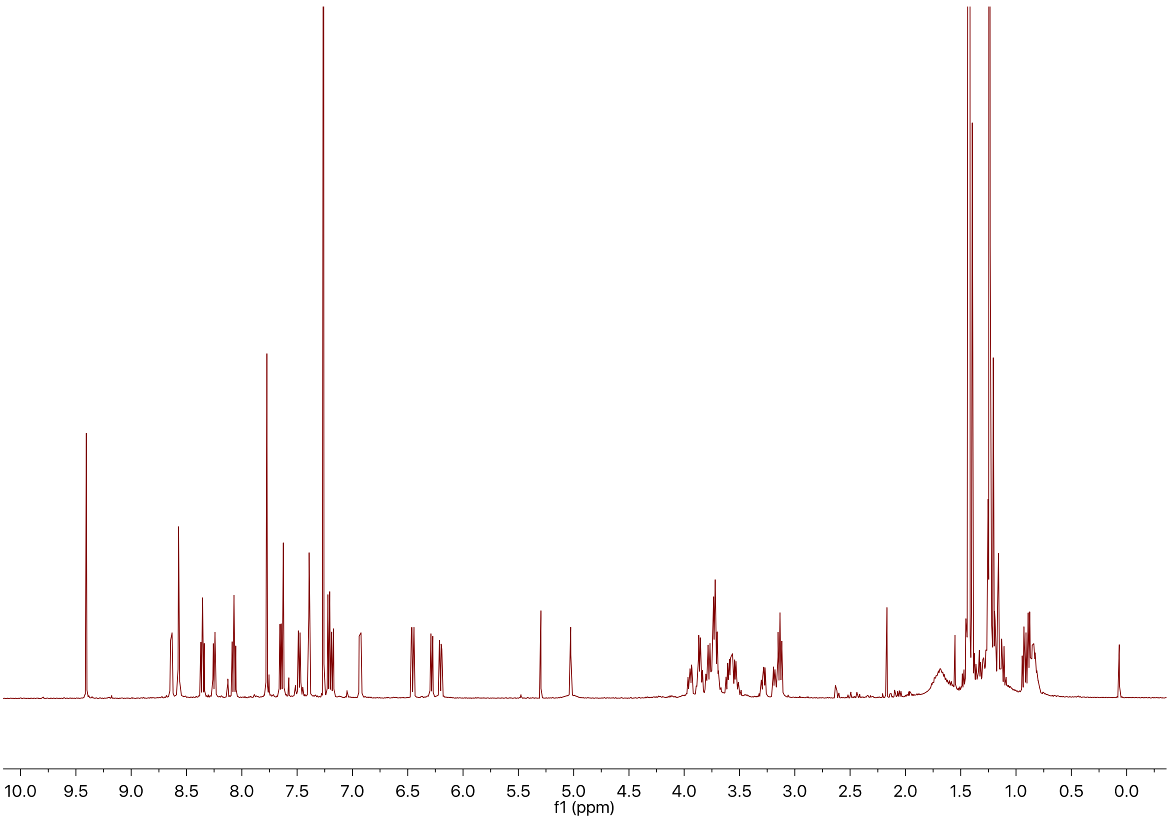

Figure S7. ^{1}H NMR ($CDCl_3$, 500 MHz) of [Pt(**1**)]BF_4

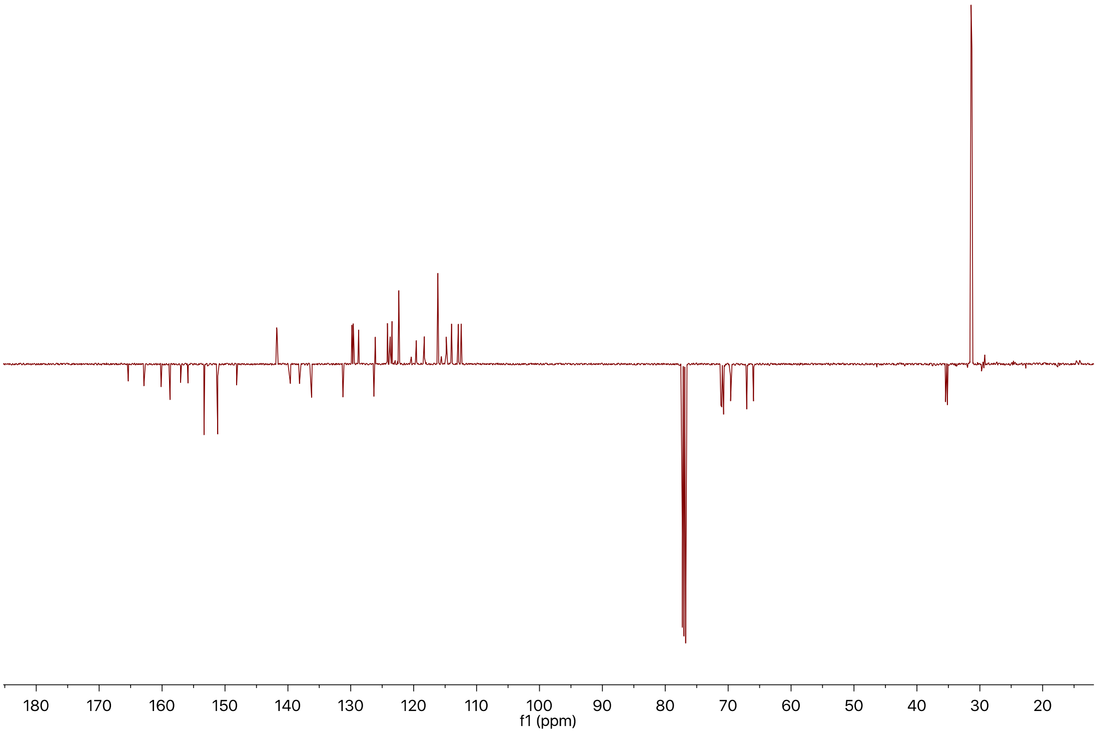

Figure S8. ^{13}C NMR ($CDCl_3$, 126 MHz) of [Pt(**1**)]BF_4

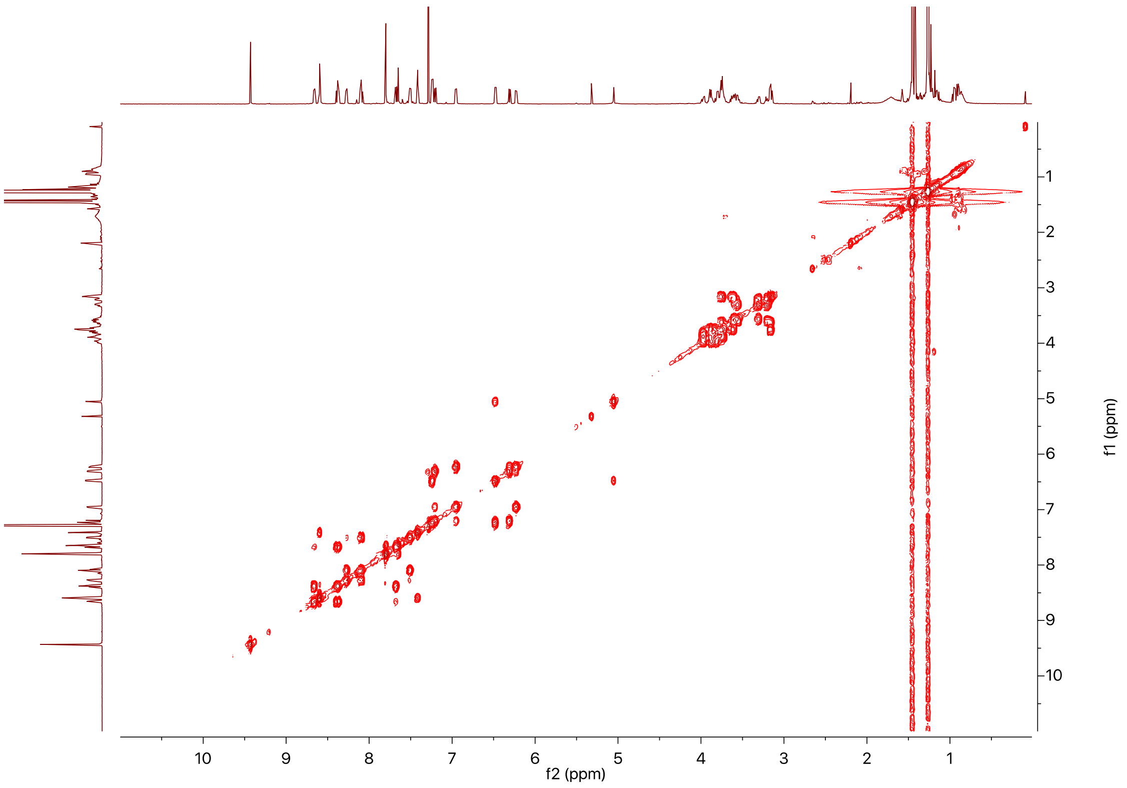

Figure S9. COSY NMR ($CDCl_3$, 500 MHz) of [Pt(**1**)]BF_4

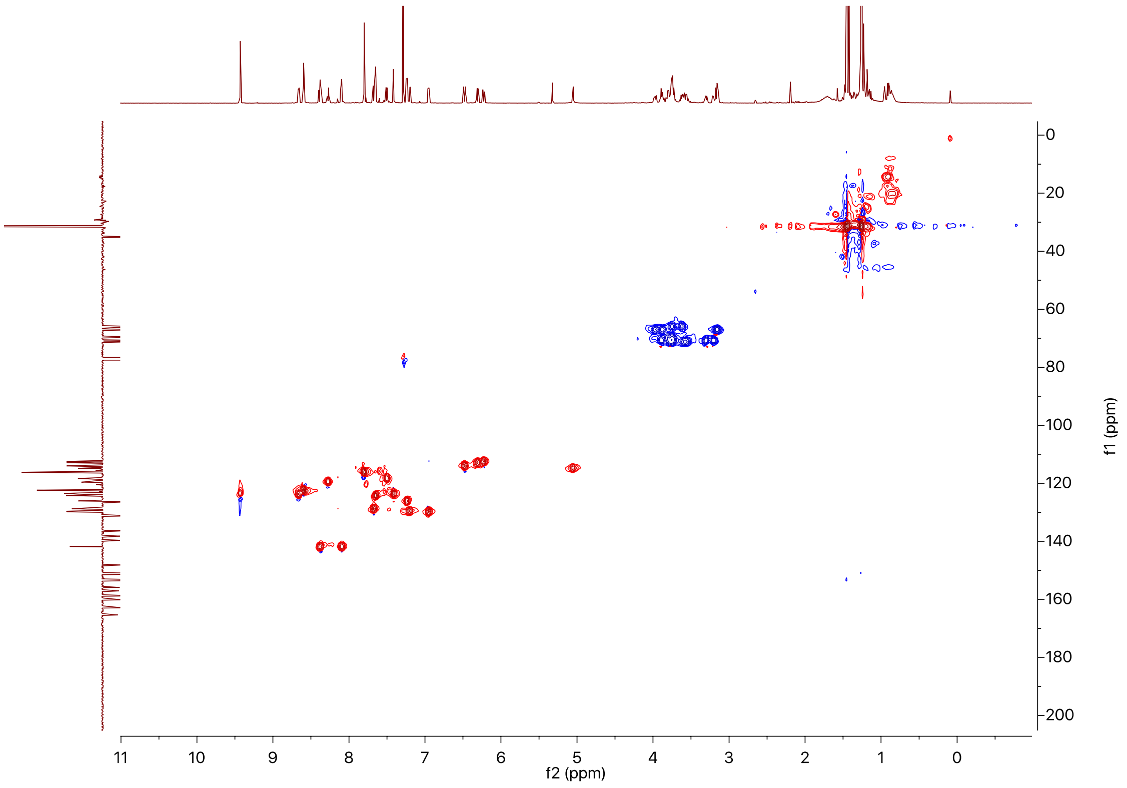

Figure S10. HSQC NMR ($CDCl_3$, 500 MHz) of [Pt(**1**)]BF_4

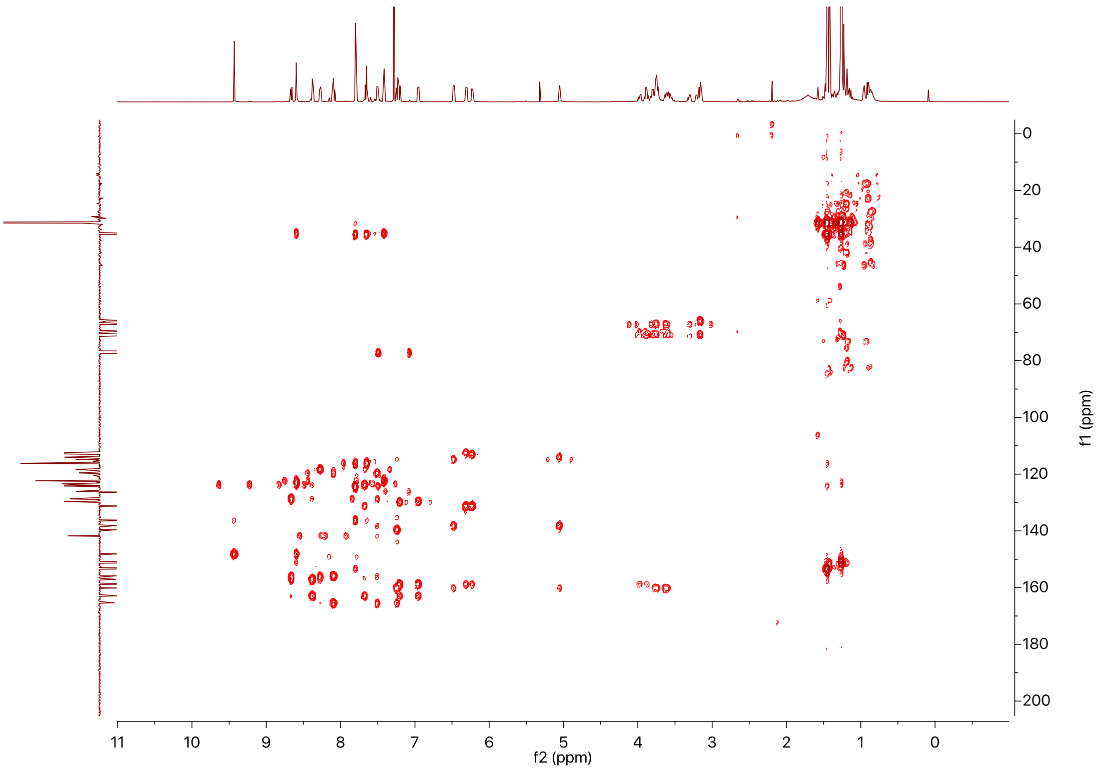

Figure S11. HMBC NMR ($CDCl_3$, 500 MHz) of [Pt(**1**)]BF_4

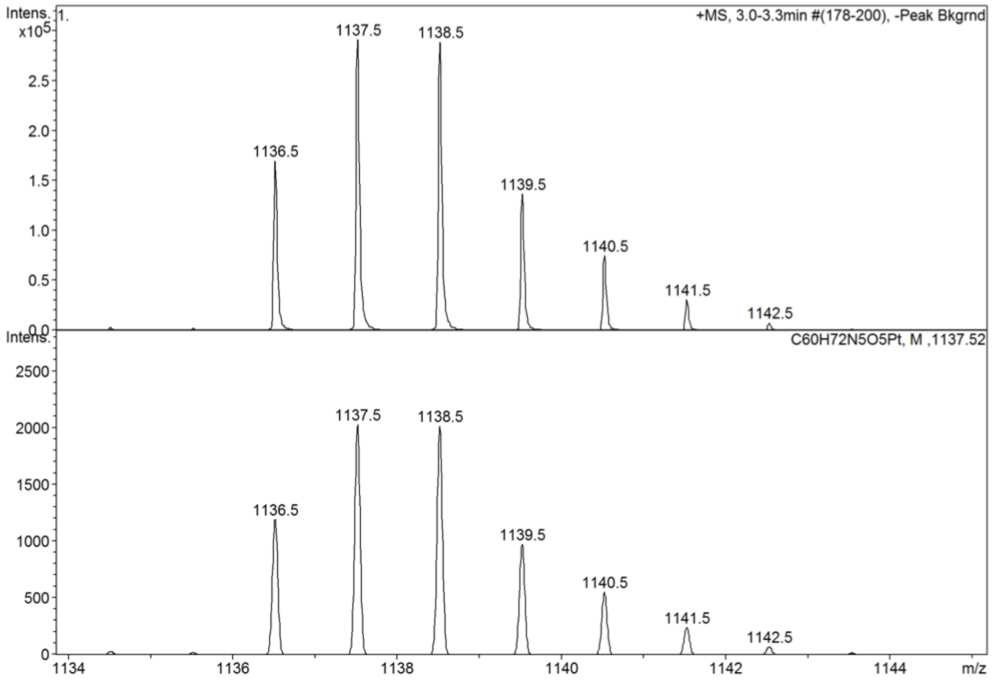

Figure S12. Isotope pattern of [Pt(**1**)]BF_4

2.3 Synthesis of 2H

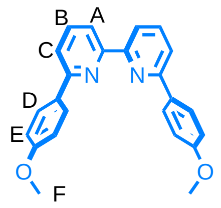

2H

[Ni(PPh_3)$_2$$Br_2$] (1.49 g, 2.00 mmol, 1 eq.), PPh_3 (1.05 g, 4.00 mmol, 2 eq.), Mn (1.10 g, 2.00 mmol, 10 eq.) and NEt_4I (0.514 g, 2.00 mmol, 1 eq.) in DMF (20 mL) were sonicated for 10 min, followed by stirring at 50 °C for 1 h. To this catalyst mixture was added **S5** (0.52 g, 2.00 mmol, 2 eq.) in DMF (20 mL), and was stirred for 4 hours. To the cooled reaction was added CH_2Cl_2 (100 mL) and EDTA-NH_3 solution (100 mL). The organic phase was washed by H_2O (100 mL), brine (100 mL) separately, followed by drying over $MgSO_4$. After solvent removal *in vacuo*, the pale-yellow solid was dissolved in CH_2Cl_2 (100 mL), acetone (300 mL) was added, the product was precipitated as white solid (300 mg, yield: 82%). Due to the poor soliblity of **2H**, the NMR spetra were obtained by adding 5% TFA to $CDCl_3$. ^1H NMR (400 MHz, 5% TFA + $CDCl_3$) δ 8.33 (s, 4H, H_A + H_B), 8.07 (d, *J* = 5.8 Hz, 2H, H_C), 7.95 (d, *J* = 8.5 Hz, 4H, H_D), 7.14 (d, *J* = 8.5 Hz, 4H, H_E), 3.93 (s, 6H, H_F). ^{13}C NMR (101 MHz, 5% TFA + $CDCl_3$) δ 163.0, 155.0, 145.4, 144.0, 128.8, 125.6, 124.4, 120.7, 115.5, 55.7. HR-ESI-MS *m/z* (found) = 368.1525; $[M+H]^+$ *m/z* (calc.) 369.1600.

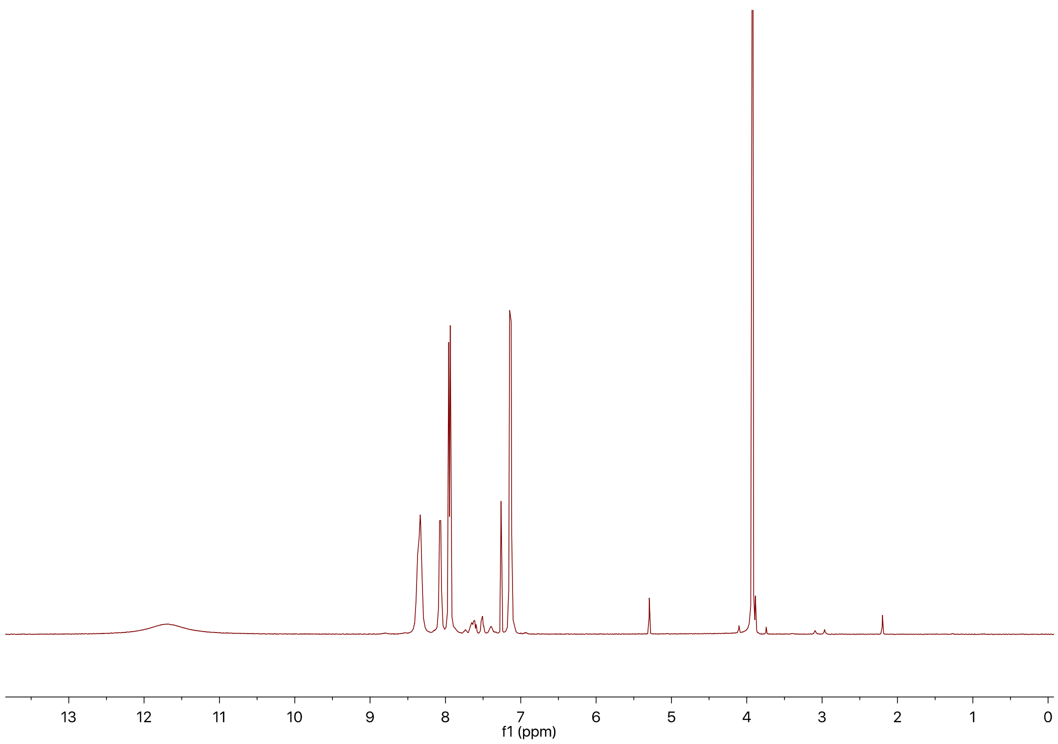

Figure S13. ^1H NMR ($CDCl_3$, 400 MHz) of **2**H

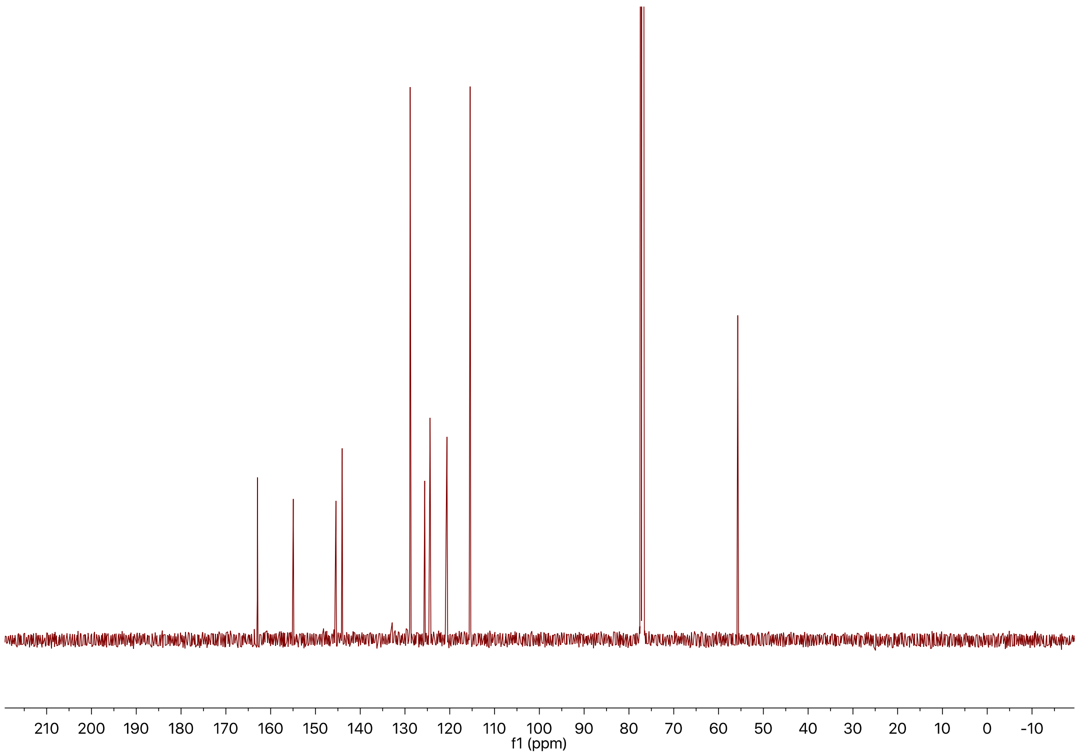

Figure S14. ^{13}C NMR ($CDCl_3$, 126 MHz) of **2**H

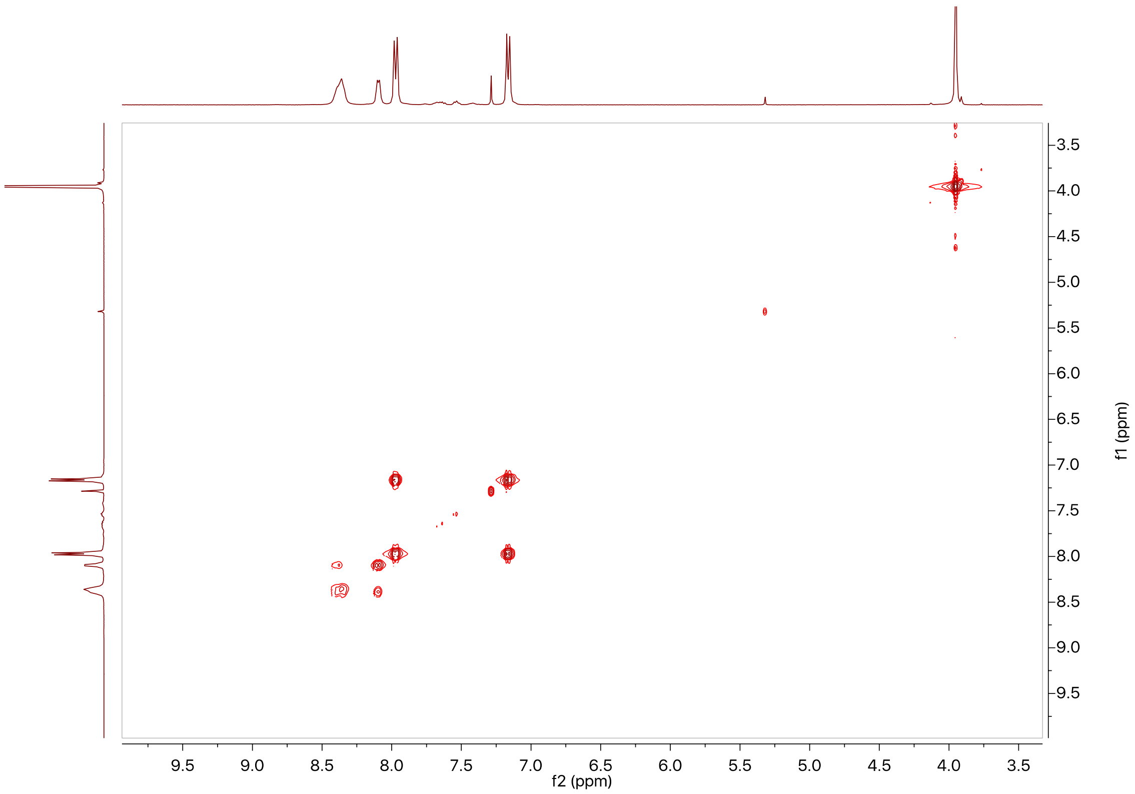

Figure S15. COSY NMR ($CDCl_3$, 400 MHz) of **2**H

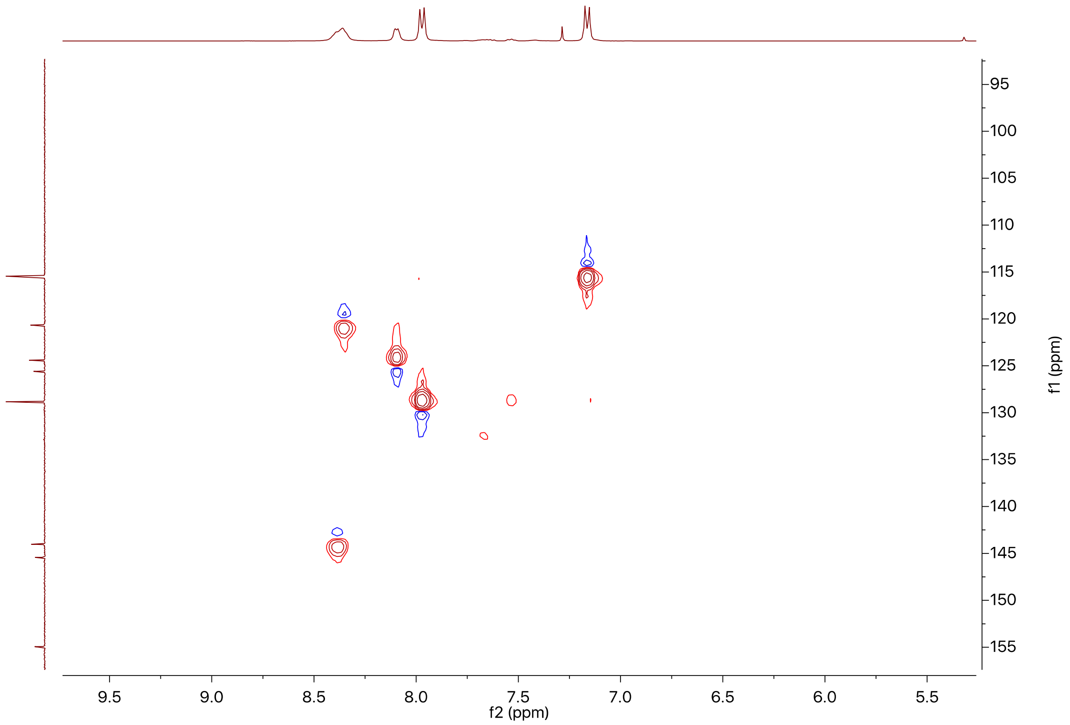

Figure S16. HSQC NMR ($CDCl_3$, 400 MHz) of **2**H

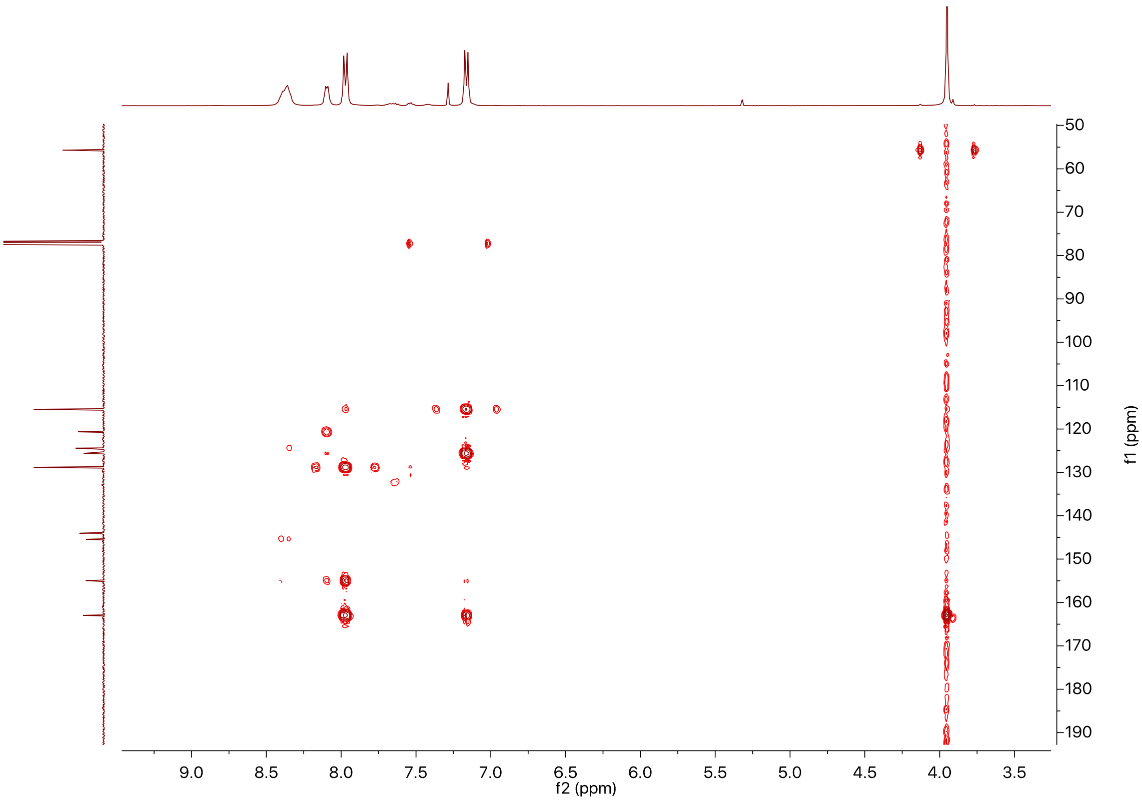

Figure S17. HMBC NMR ($CDCl_3$, 400 MHz) of **2**H

2.4 Synthesis of [Pt(2)Cl]

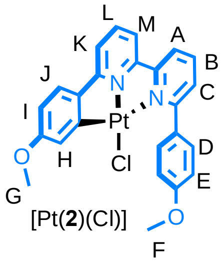

[Pt(**2**)(Cl)]

2H (33 mg, 0.09 mmol, 1eq), K_2PtCl_4 (44 mg, 0.11 mmol, 1.2 eq) in glacial acetic acid (2 mL) was stirred at 120 ºC overnight. The yellow mixture was washed with saturated NH_4BF_4 solution (2 × 30 mL) and aqueous phase was extracted with CH_2Cl_2 (2 × 20 mL). The organic phase was dried over $MgSO_4$ and the solvent was evaporated *in vacuo*. The yellow mixture was purified by flash column chromatography (0 to 5% MeOH in CH_2Cl_2), affording bright yellow solid (28 mg, 53%). ^1H NMR (400 MHz, DMSO-*d*$_6$) δ 8.45 (d, *J* = 7.1 Hz, 1H, H_A), 8.28 (t, *J* = 7.9 Hz, 1H, H_B), 8.19 – 7.99 (m, 2H, H_K + H_M), 7.94 – 7.78 (m, 2H, H_C + H_L), 7.63 (d, *J* = 8.7 Hz, 2H, H_D), 7.56 (d, *J* = 8.6 Hz, 1H, H_J), 7.08 (d, *J* = 2.6 Hz, 1H, H_H), 6.99 (d, *J* = 8.7 Hz, 2H, H_E), 6.64 (dd, *J* = 8.5, 2.6 Hz, 1H, H_I), 3.83 (s, 3H, H_F), 3.72 (s, 3H, H_G). ^{13}C NMR (101 MHz, DMSO-*d*$_6$) δ 164.8, 163.0, 160.8, 160.4, 157.4, 155.0, 140.6, 139.6, 138.8, 132.1, 131.5, 129.5, 126.7, 121.7, 119.2, 118.9, 118.6, 113.7, 110.0 (2), 55.6, 55.3. HR-ESI-MS *m/z* (found) $[M-Cl]^+$ = 562.1073; $[M-Cl]^+$ *m/z* (calc) = 562.1094.

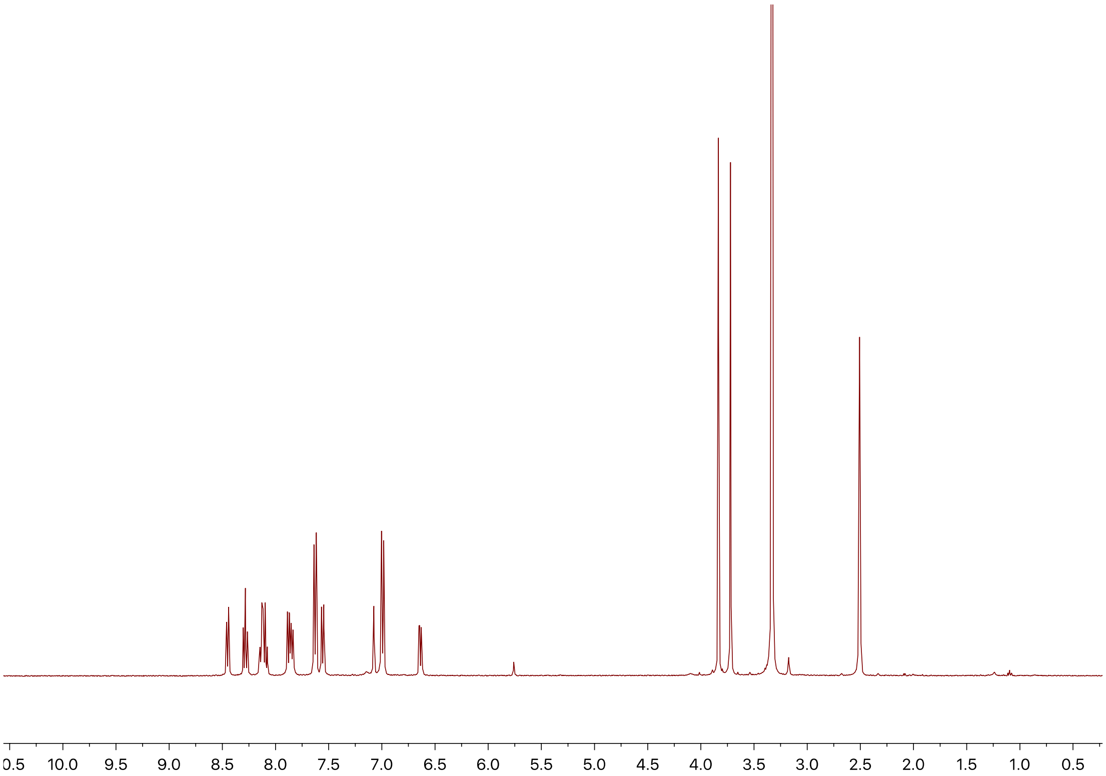

Figure S18. ^1H NMR (DMSO-d$_6$, 400 MHz) [Pt(**2**)Cl]

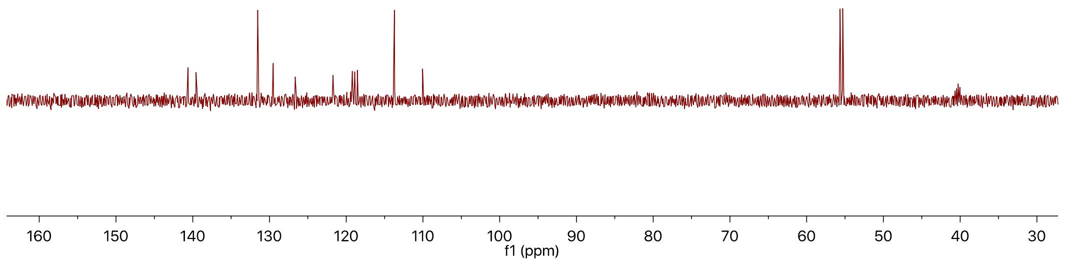

Figure S19. ^{13}C NMR (DMSO-d_6, 101 MHz) [Pt(**2**)Cl]

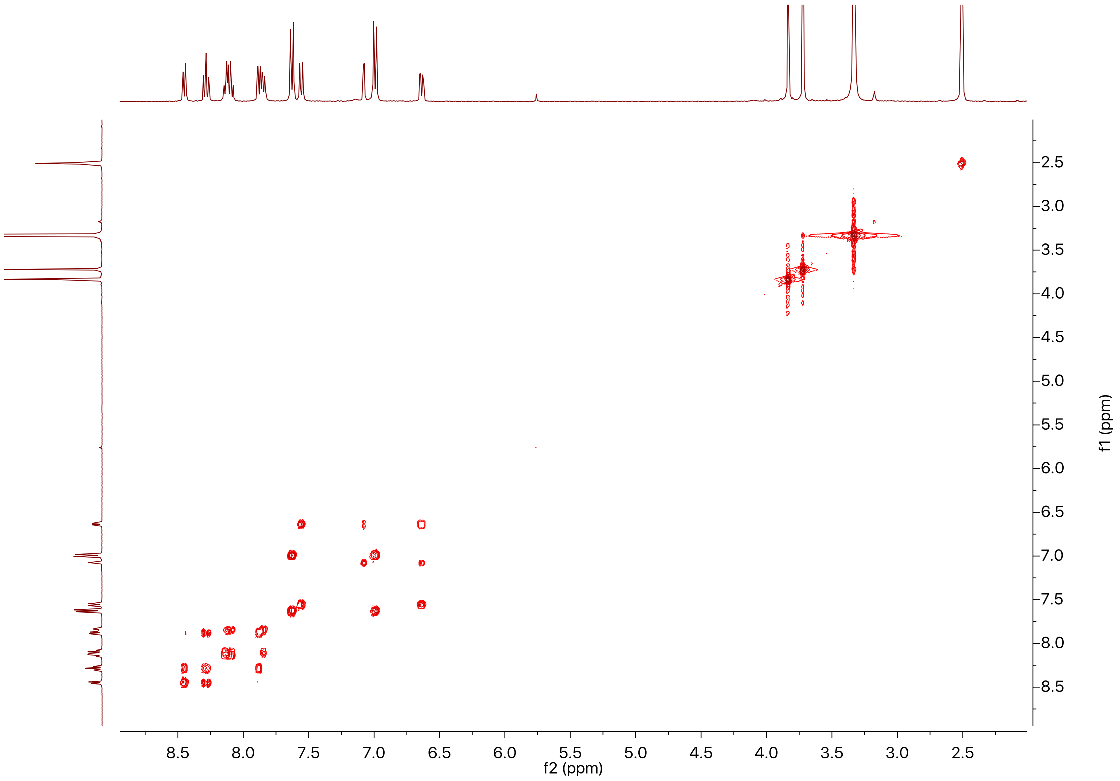

Figure S20. COSY NMR (DMSO-d_6, 400 MHz) [Pt(**2**)Cl]

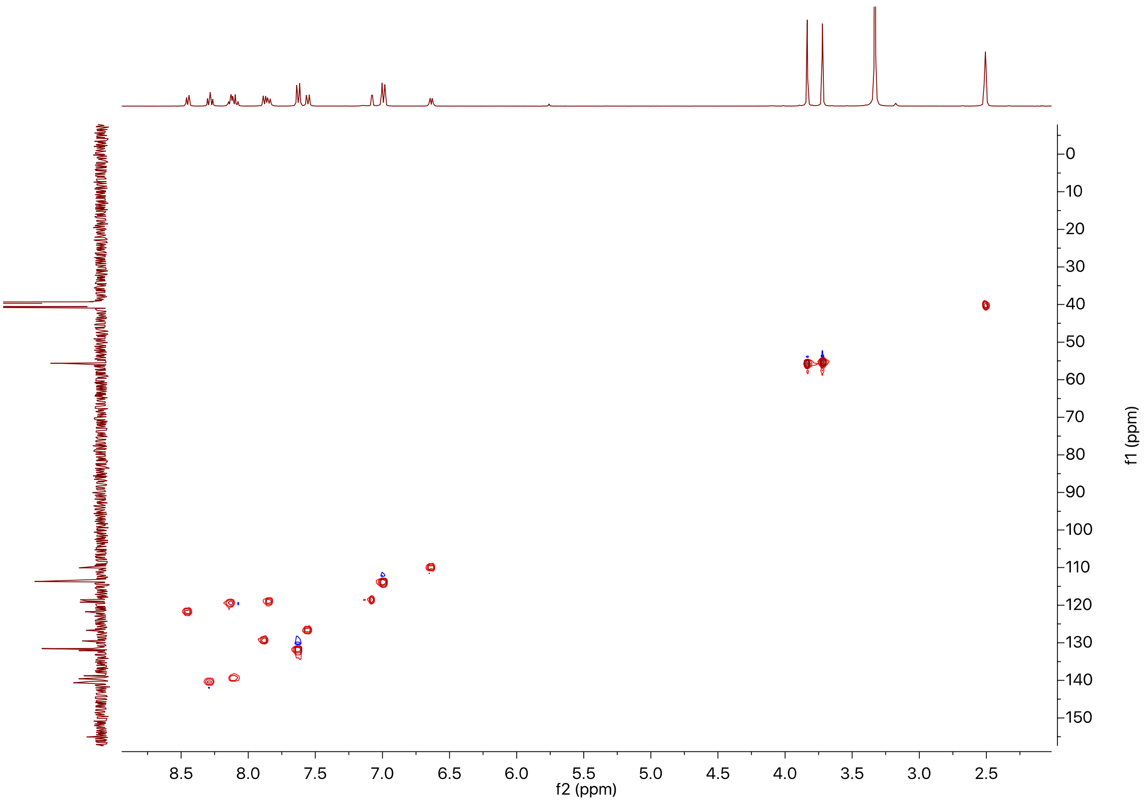

Figure S21. HSQC NMR (DMSO-d$_6$, 400 MHz) [Pt(**2**)Cl]

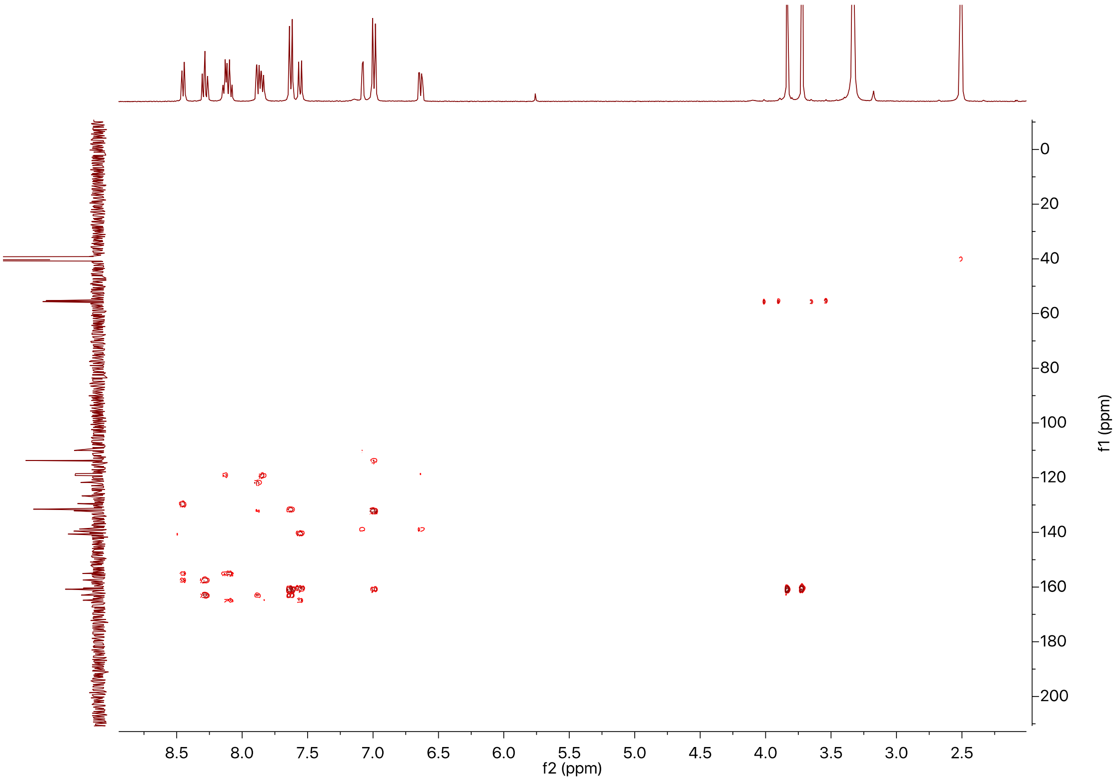

Figure S22. HMBC NMR (DMSO-d$_6$, 400 MHz) [Pt(**2**)Cl]

2.5 Synthesis of [Pt(2)(3)]BF4

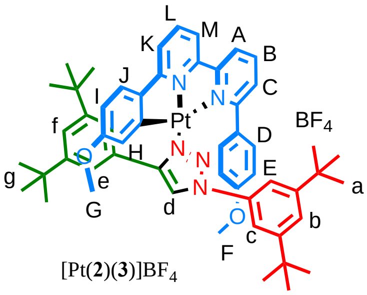

[Pt(**2**)(**3**)]BF_4

[Pt(**2**)(Cl)] (32.6 mg, 0.05 mmol, 1eq), **3** (26.7 mg, 0.06 mmol, 1.2 eq) , $AgSbF_6$ (17.2 mg, 0.05 mmol, 1 eq) were mixed in CH_2Cl_2 (2 mL) and heated at 60ºC for overnight. The yellow suspension were diluted with CH_2Cl_2 (15 mL), and then washed with saturaed NH_4BF_4 (20 mL). The organic phase was dried over $MgSO_4$ and the solvent was removed *in vacuo*. The crude product was purified by flash column chromatography (5% MeOH in CH_2Cl_2), affording the product as yellow oil (30 mg, 50 %). ^1H NMR (500 MHz, $CDCl_3$) δ 8.35 (dd, *J* = 8.0, 1.1 Hz, 1H, H_C), 8.19 – 8.14 (m, 1H, H_B), 8.09 – 8.07 (m, 5H, H_d + H_e + H_L + H_K), 7.69 (t, *J* = 1.7 Hz, 1H, H_b), 7.66 (d, *J* = 1.7 Hz, 2H, H_c), 7.63 – 7.58 (m, 1H, H_M), 7.48 – 7.42 (m, 3H, H_J + H_A + one of H_D), 7.37 (t, *J* = 1.8 Hz, 1H, H_f), 6.89 (br m, 1H, one of H_E), 6.73 (br m, 1H, one of H_D), 6.68 (dd, *J* = 8.6, 2.5 Hz, 1H, H_I), 6.46 (br m, one of H_E), 5.73 (d, *J* = 2.5 Hz, 1H, H_H), 3.65 (s, 3H, H_G), 3.57 (s, 3H, H_F), 1.48 (s, 18H, H_a), 1.20 (s, 18H, H_g). ^{13}C NMR (126 MHz, $CDCl_3$) δ 165.4, 162.4, 161.3, 160.8, 157.0, 155.4, 153.7, 151.2, 149.8, 141.4, 141.1, 138.6, 138.3, 135.5, 131.6, 130.0, 128.9, 126.7, 125.7, 124.8, 124.2, 122.4, 122.0, 120.8, 119.2, 119.0, 118.5, 115.4, 112.8, 109.3, 55.3, 55.0, 35.4, 35.0, 31.3, 31.1. HR-ESI-MS: *m/z* (found) = 1007.5, *m/z* (cal) = 1007.5. [M-BF_4]$^+$.

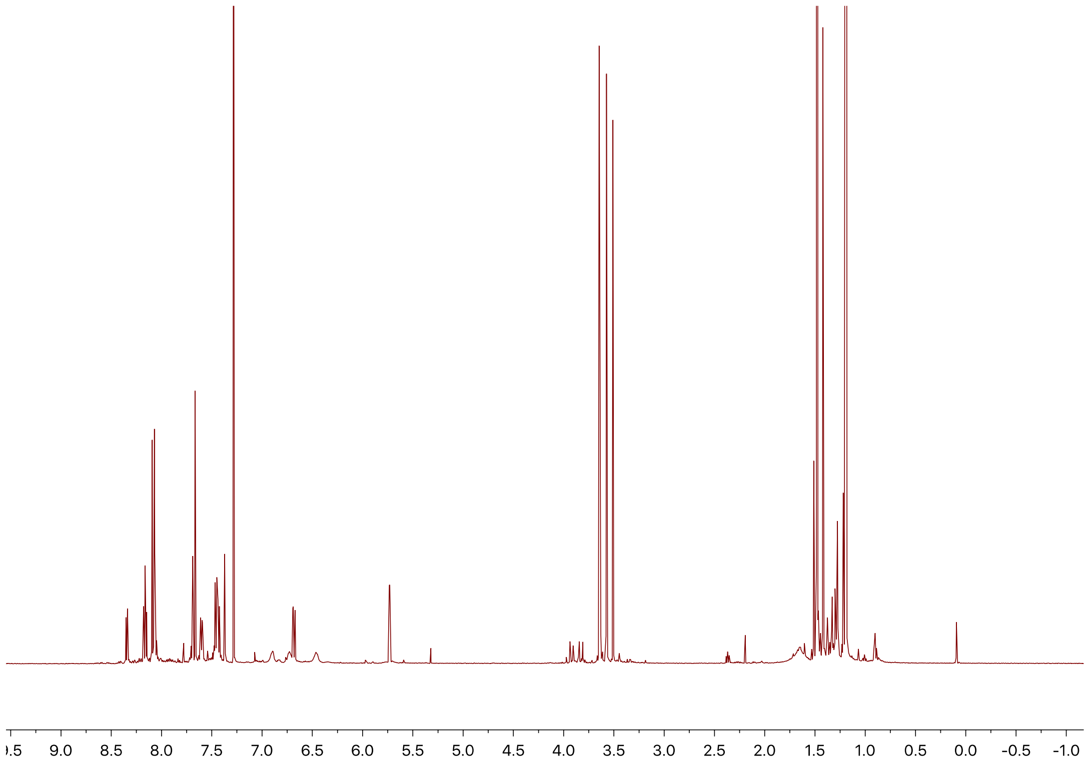

Figure S23. ^1H NMR ($CDCl_3$, 500 MHz) of [Pt(**2**)(**3**)]BF_4

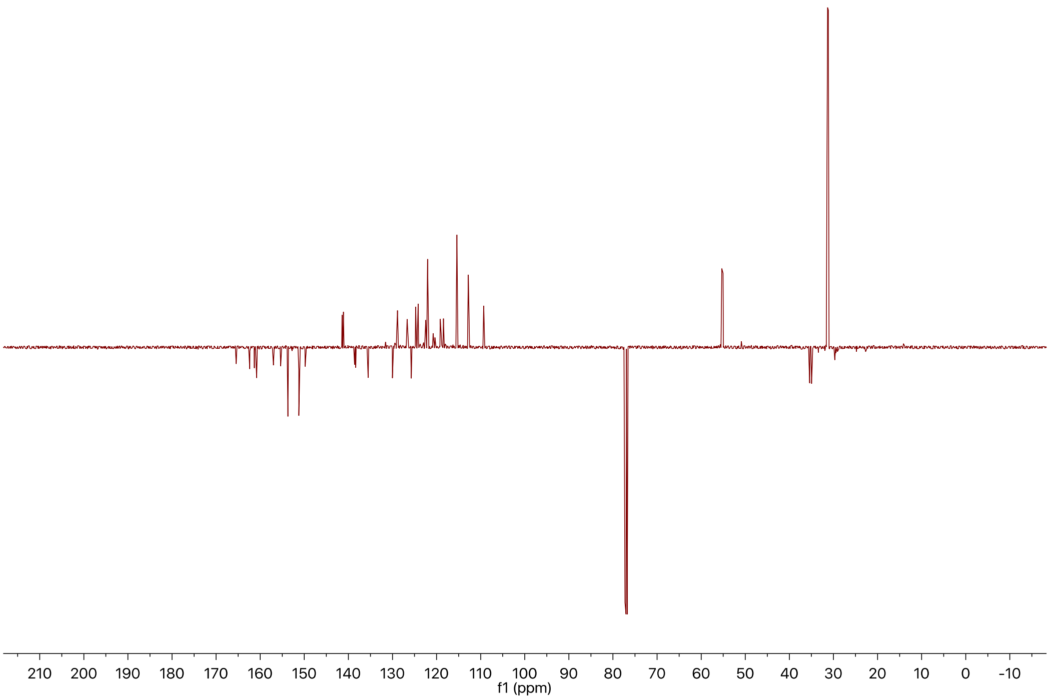

Figure S24. ^{13}C NMR ($CDCl_3$, 126 MHz) of [Pt(**2**)(**3**)]BF_4

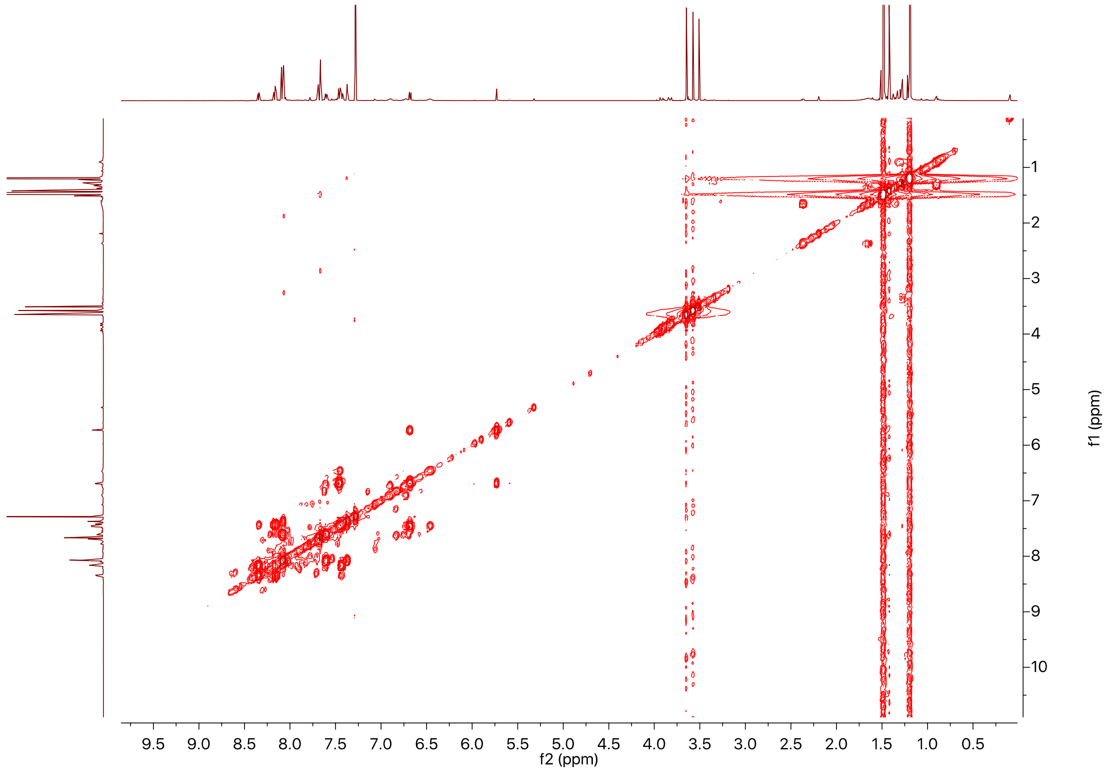

Figure S25. COSY NMR ($CDCl_3$, 500 MHz) of [Pt(**2**)(**3**)]BF_4

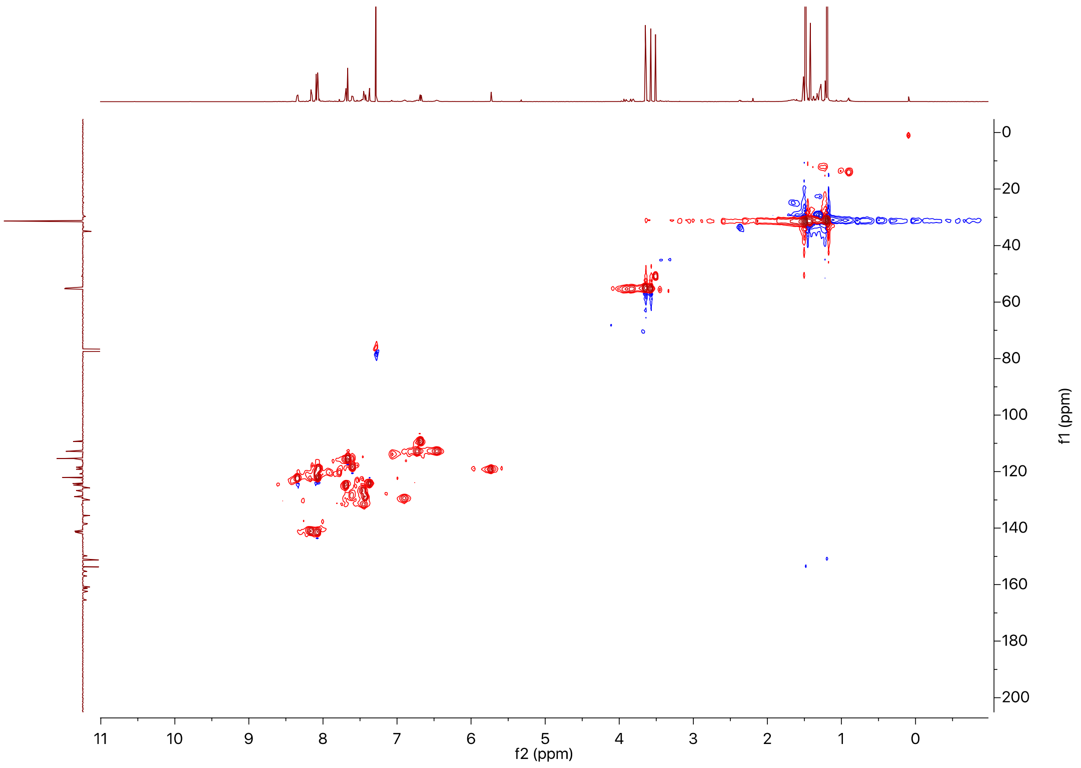

Figure S26. HSQC NMR ($CDCl_3$, 500 MHz) of [Pt(**2**)(**3**)]BF_4

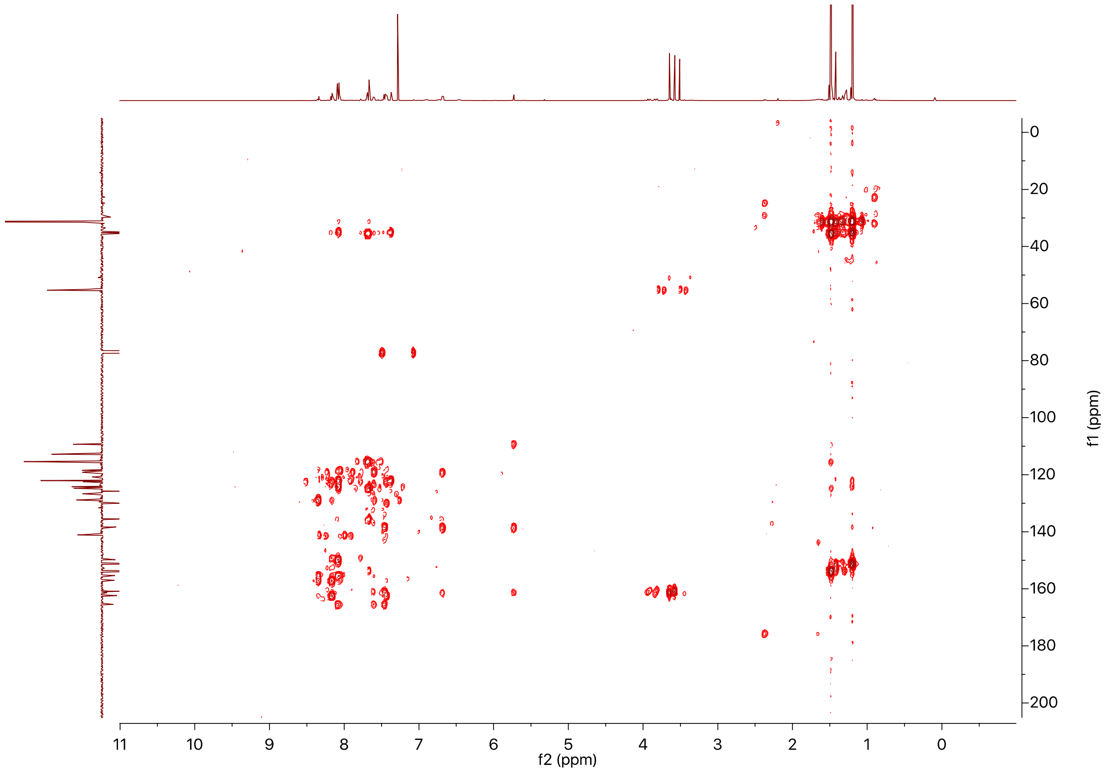

Figure S27. HMBC NMR ($CDCl_3$, 500 MHz) of [Pt(**2**)(**3**)]BF_4

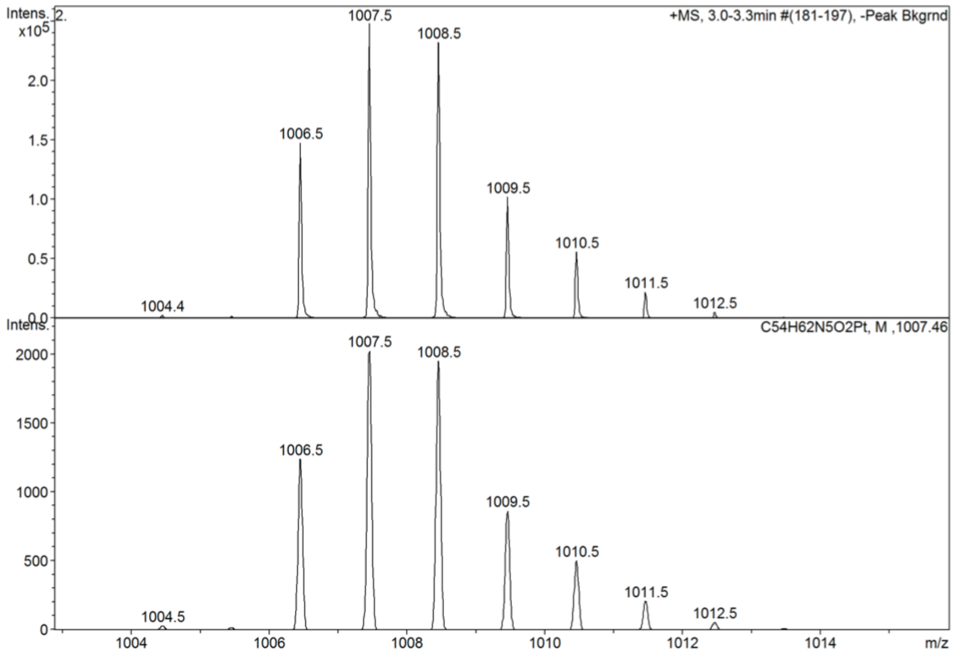

Figure S28. Isotope pattern of [[Pt(**2**)(**3**)]BF_4

2.6 Synthesis of 4H

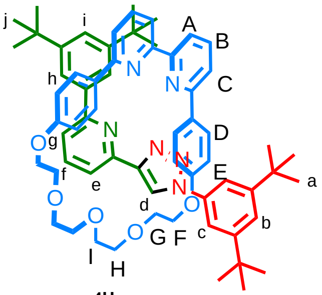

4H

A solution of **S2** (34.9 mg, 0.12 mmol, 1.2 eq), **S4** (27.7 mg, 0.12 mmol, 1.2 eq), **S1** (49.8 mg, 0.10 mmol, 1 eq), [$Cu(MeCN)_4$]PF_6, (35.8 mg, 0.096 mmol. 0.96 eq) and $^i Pr_2EtN$ (0.020 mL, 0.40 mmol, 4 eq) in 1:1 CH_2Cl_2/MeCN (2 mL) was stirred at r.t. overnight. The solvent was removed *in vacuo* and a red crude residue was obtained. The residue was dissolved in CH_2Cl_2 (10 mL) and KCN (0.070 mg, 10 eq) in MeOH (5 mL) was added. After stirring for 3h, the yellow solution was washed with H_2O (2 × 20 mL) solution and organic phase was dried over $MgSO_4$ and the solvent was removed *in vacuo*. The yellow mixture was purified by flash column chromatography (0 to 10% MeCN in 1:1 CH_2Cl_2/Petrol), affording white solid (70 mg, 78%). ^1H NMR (400 MHz, $CDCl_3$) δ 10.75 (s, 1H, H_d), 7.74 (d, J = 1.8 Hz, 2H, H_c), 7.67 (m, 3H, HB + H_e), 7.53 (m, 4H, $H_A + H_f + H_g$), 7.41 (d, J = 1.7 Hz, 2H, H_i), 7.40 – 7.34 (m, 3H, $H_b + H_C$), 7.28-7.26 (m, 4H, H_E), 7.22 (t, J = 1.7 Hz, 1H, H_h), 6.26 (d, J = 8.7 Hz, 4H, H_D), 4.08 – 3.83 (m, 12H, $H_F + H_H + H_I$), 3.71 – 3.56 (m, 4H, H_G), 1.24 (s, 18H, H_j), 1.13 (s, 18H, H_a). ^{13}C NMR (101 MHz, $CDCl_3$) δ 159.5, 158.5, 157.8, 157.4, 151.2, 151.1, 150.5, 146.9, 139.3, 136.9, 136.6, 136.2, 132.5, 129.0, 127.0, 122.5, 121.6, 121.4, 119.7,

119.3, 119.1, 118.7, 114.7, 114.1, 77.2, 69.8, 68.4, 66.4, 34.8, 34.8, 31.4, 31.2. HR-MS-ESI: *m/z* (found) $[M+H]^+$ = 1021.6, *m/z* (calc.) $[M+H]^+$ = 1021.6.

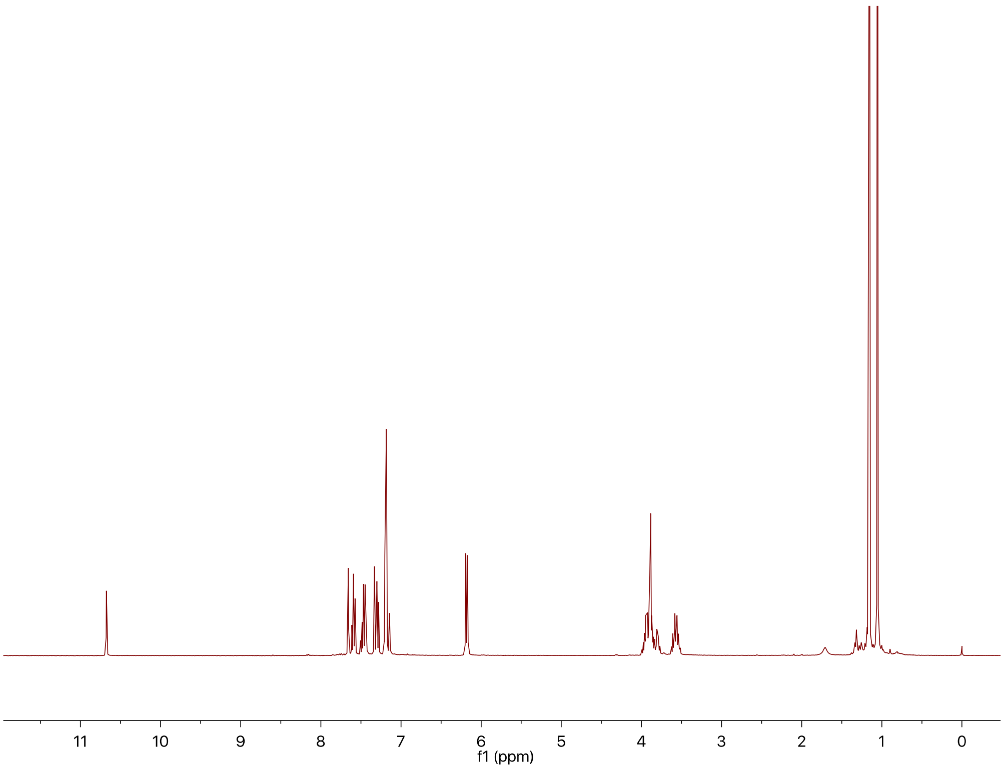

Figure S29. ^1H NMR ($CDCl_3$, 400 MHz) of **4**H

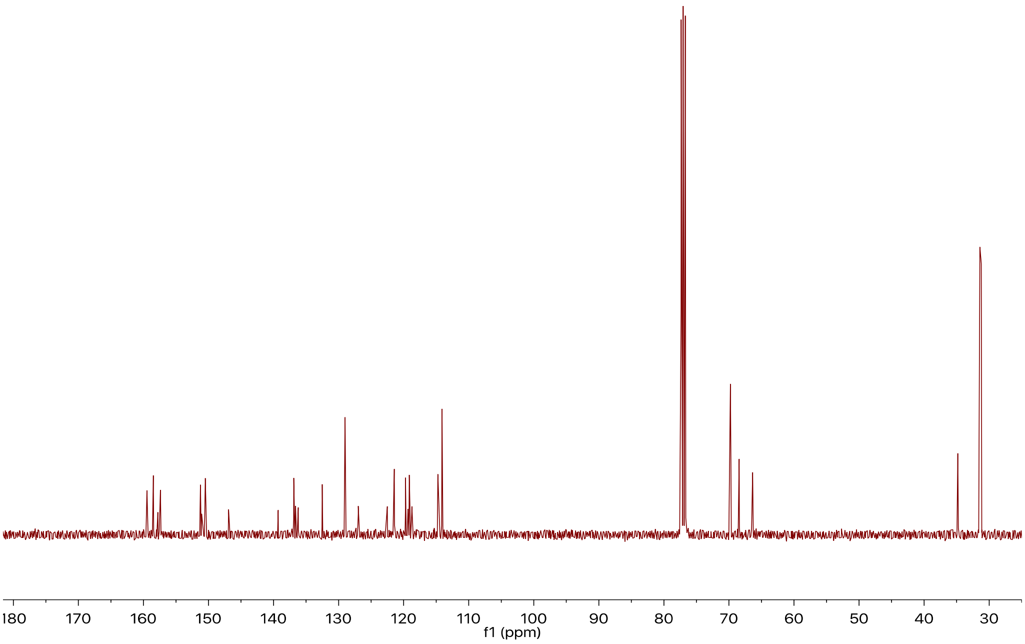

Figure S30. ^{13}C NMR ($CDCl_3$, 126 MHz) of **4**H

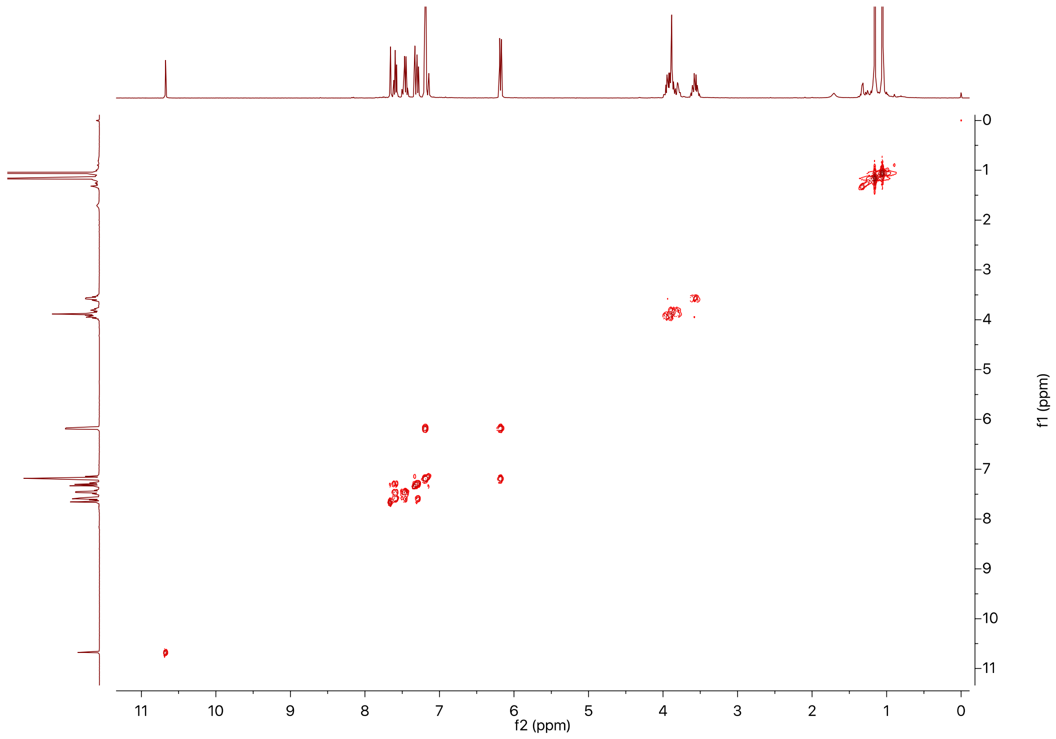

Figure S31. COSY NMR ($CDCl_3$, 400 MHz, 400 MHz) of **4**H

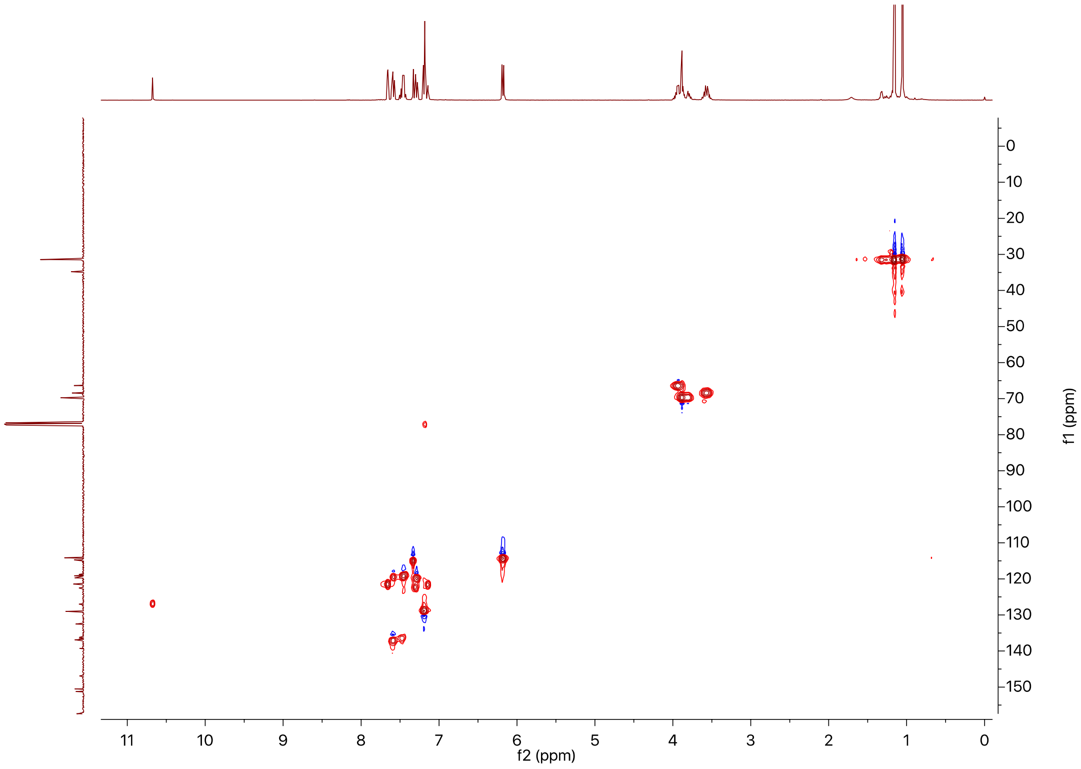

Figure S32. HSQC NMR ($CDCl_3$, 400 MHz) of **4**H

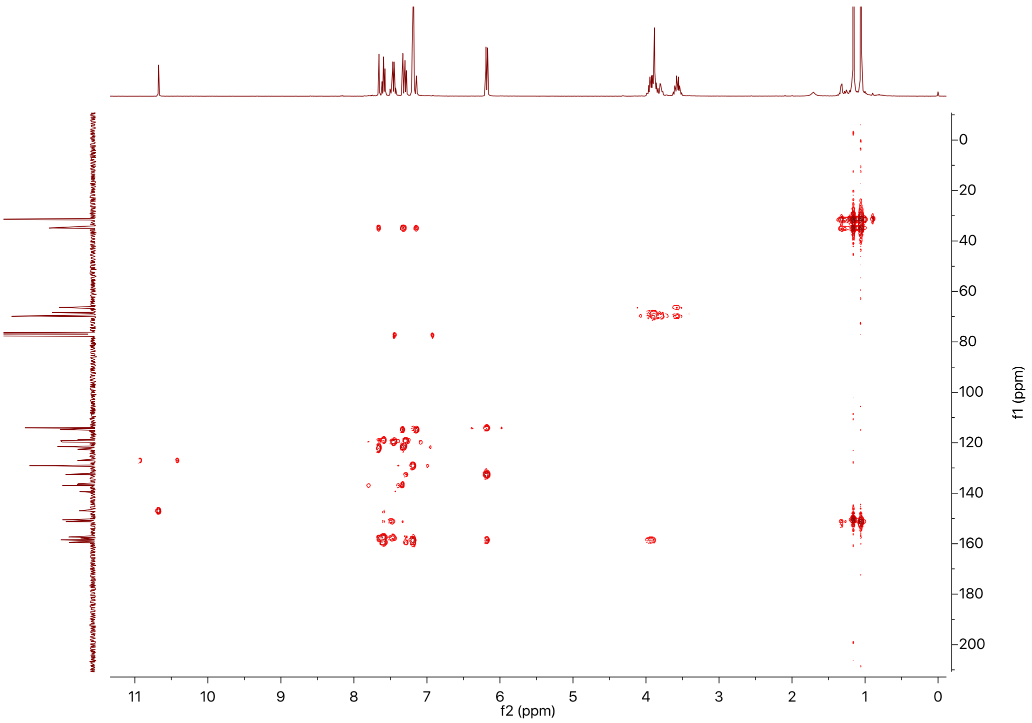

Figure S33. HMBC NMR ($CDCl_3$, 400 MHz) of **4**H

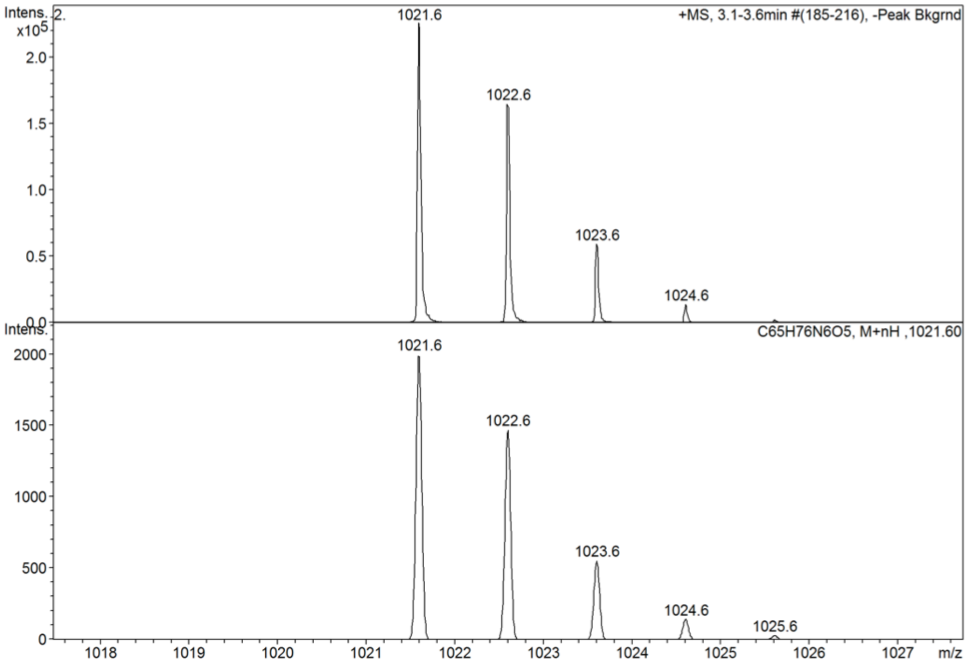

Figure S34. Isotope pattern of **4**H

2.7 Synthesis of [Pt(4)]BF_4

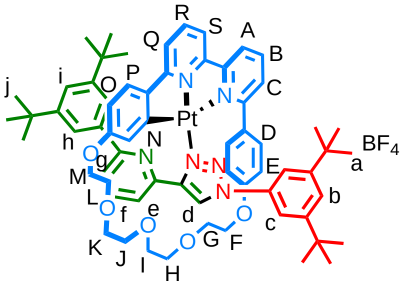

[Pt(**4**)]BF_4

A mixture of **4**H (28 mg, 0.027 mmol, 1 eq), K_2PtCl_4 (18 mg, 0.043 mmol, 2 eq) in glacial acetic acid (1 mL) was stirred at 120 ºC overnight. After cooling to room temperature, the yellow suspension was added with CH_2Cl_2 (15 mL), and washed by saturated NH_4BF_4 solution (2 × 15 mL). The organic phase was dried over $MgSO_4$ and the solvent was removed *in vacuo*. The yellow mixture was purified by flash column chromatography (0 to 2% MeOH in CH_2Cl_2), affording yellow solid (15 mg, 47%); Melting point: 198-202 ºC. ^{1}H NMR (500 MHz, $CDCl_3$) δ 9.63 (s, 1H, H_d), 8.61 (d, *J* = 7.4 Hz, 1H, H_e), 8.39 (dd, *J* = 8.1, 1.2 Hz, 1H, H_A), 8.27 (app t, *J* = 7.9 Hz, 1H, H_B), 8.04 – 7.93 (m, 5H, H_h + H_Q + H_f + H_S), 7.79 (dd, *J* = 8.8, 1.2 Hz, 3H, H_c + H_g), 7.68 – 7.60 (m, 2H, H_C + H_b), 7.47 – 7.42 (m, 2H, H_i +H_R), 7.38 (dd, *J* = 8.4, 2.3 Hz, 1H, one of H_E), 7.19 (d, *J* = 8.6 Hz, 1H, H_P), 7.10 (dd, *J* = 8.4, 2.3 Hz, 1H, one of H_E), 6.46 (dd, *J* = 8.5, 2.5 Hz, 1H, H_O), 6.26 (dd, *J* = 8.4, 2.6 Hz, 1H, one of H_D), 6.18 (dd, *J* = 8.4, 2.6 Hz, 1H, one of H_D), 5.33 (d, *J* = 2.6 Hz, 1H, H_N), 3.99 – 3.82 (m, 4H, 1 of H_M, 1 of H_H, H_F), 3.75 (q, J = 8.5 Hz, 1H, 1 of H_H or H_I), 3.70 (m, 3H, H_G, 1 of H_H or H_I), 3.67 – 3.55 (m, 3H, 1 of H_M, H_J), 3.45 – 3.31 (m, 3H, 1 of H_L, 1 of H_K, 1 of H_H or H_I), 3.21 (dq, J = 8.8, 5.0 Hz, 1H, 1 of H_L), 3.12 (t, J = 8.8 Hz, 1H, 1 of H_K), 1.45 (s, 18H, H_a), 1.25 (s, 18H, H_j).^{13}C NMR (126 MHz, $CDCl_3$) δ 165.8, 163.1, 160.8, 159.0, 158.7, 157.1, 156.1, 153.3, 151.1, 147.5, 146.4, 141.2, 141.1, 139.6, 138.3, 138.3, 137.5, 136.0, 131.1, 130.7, 129.6, 128.8, 126.3, 125.8, 124.1, 123.2, 122.8, 121.8, 121.8, 121.6, 118.8, 118.3, 116.1, 115.0, 114.1, 113.4, 112.4, 71.0, 70.9, 70.7, 70.6, 69.2, 67.3, 67.3, 66.9, 35.5, 34.9, 31.4, 31.3. HR-MS-ESI: *m*/*z* (found) = 1214.5, *m*/*z* (calc.) = 1214.5.

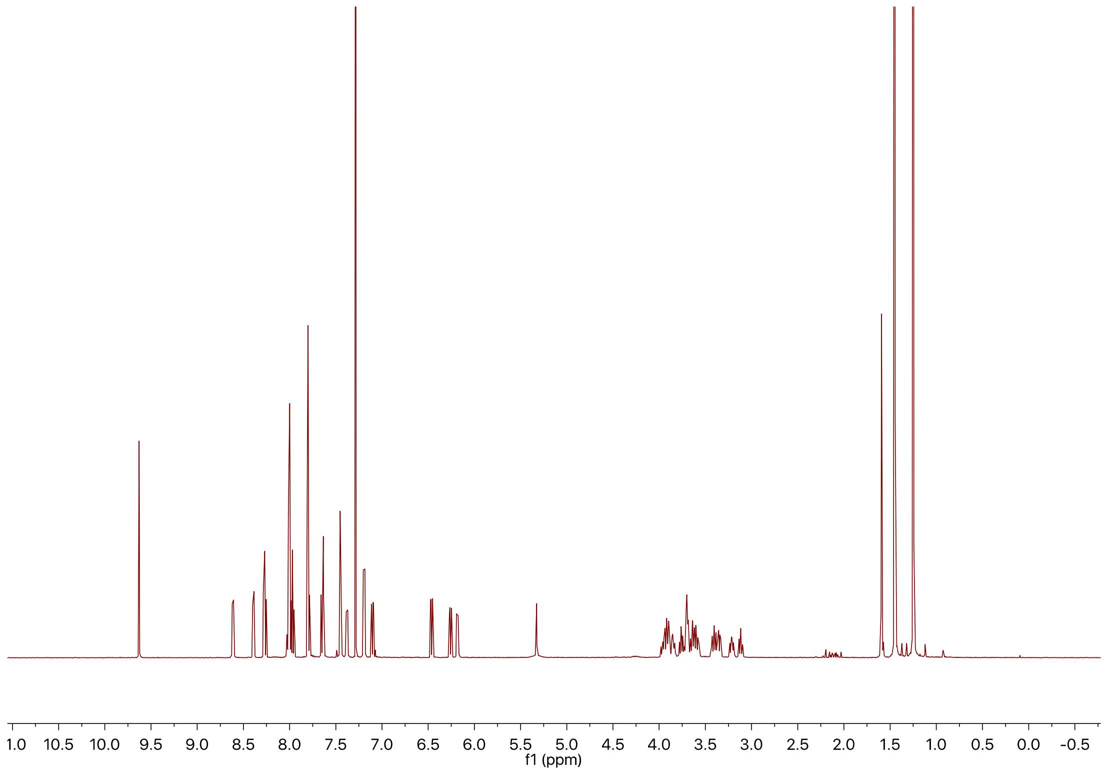

Figure S35. ^{1}H NMR ($CDCl_3$, 500 MHz) of [Pt(**4**)]BF_4

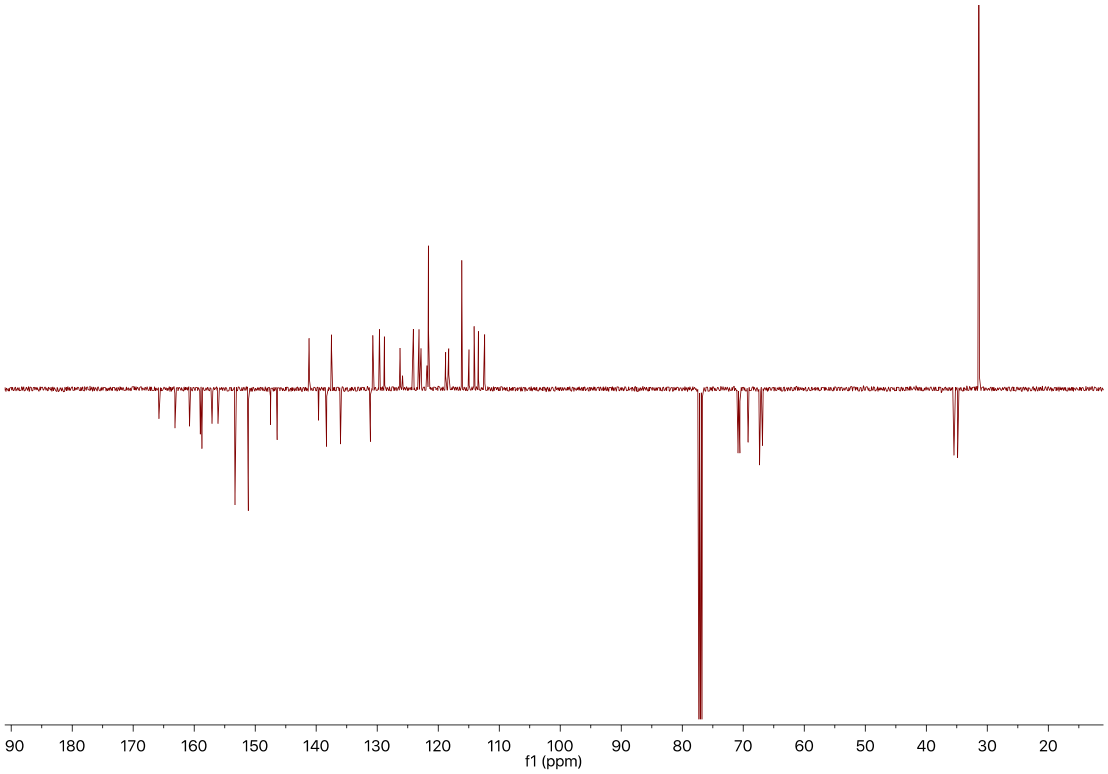

Figure S36. ^{13}C NMR ($CDCl_3$, 126 MHz) of [Pt(**4**)]BF_4

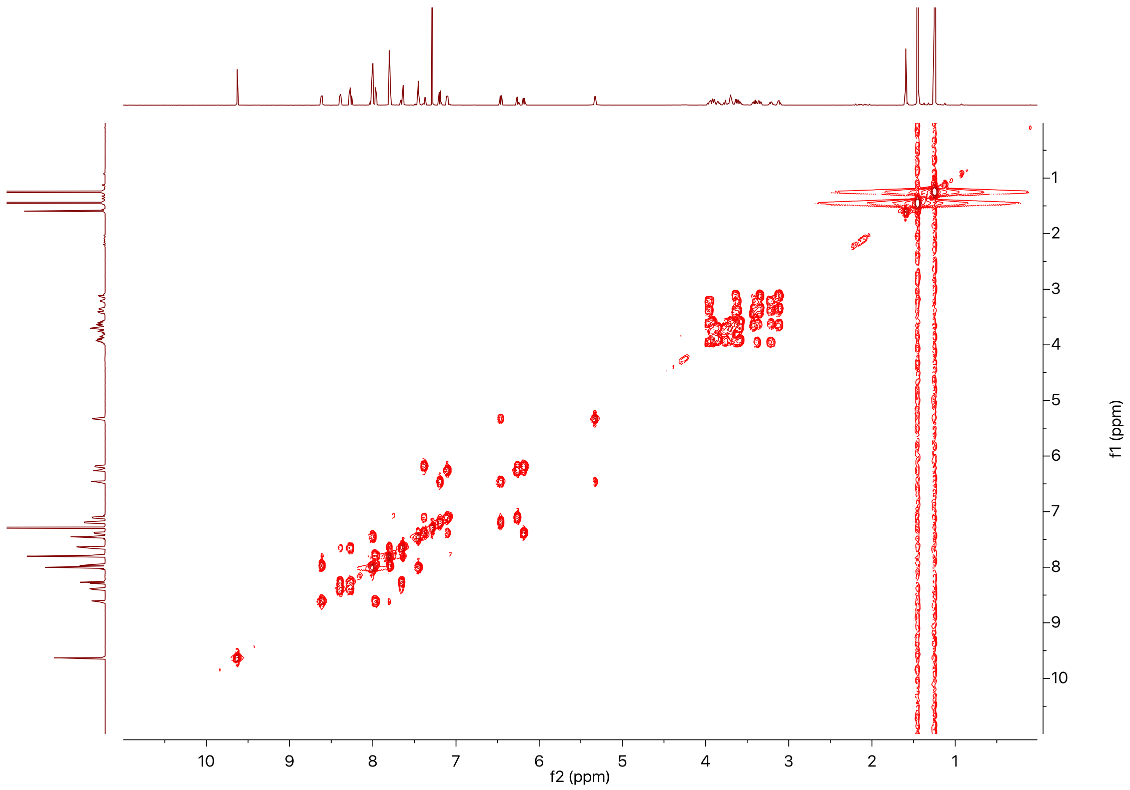

Figure S37. COSY NMR ($CDCl_3$, 500 MHz) of [Pt(**4**)]BF_4

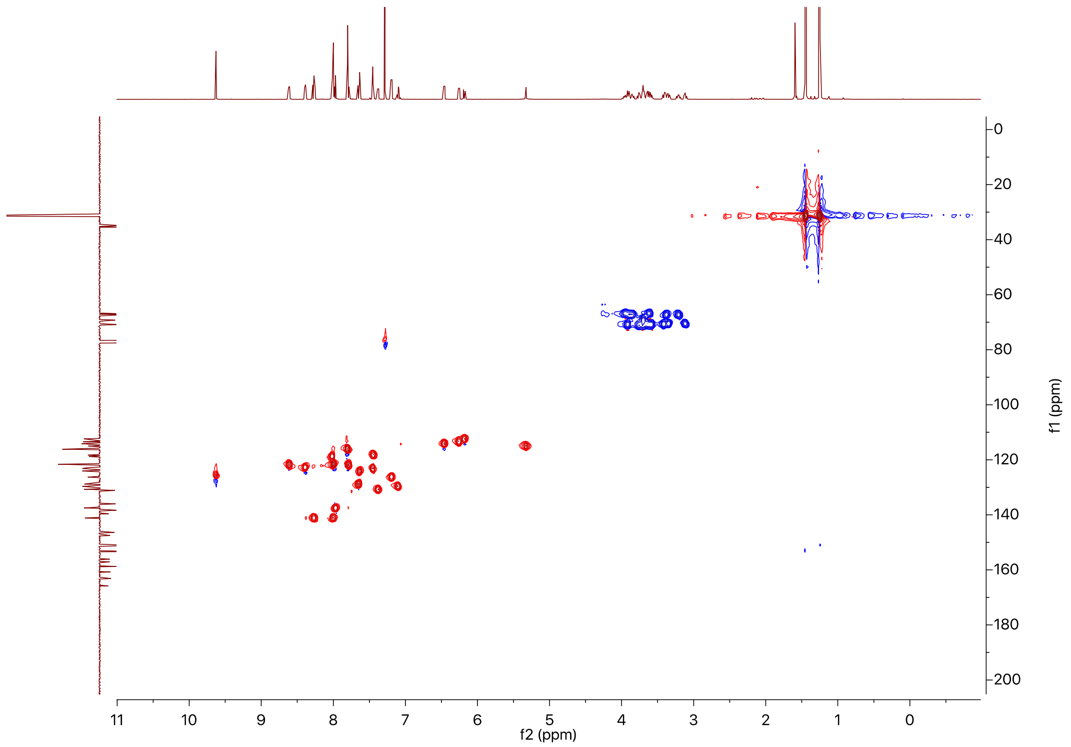

Figure S38. HSQC NMR ($CDCl_3$, 500 MHz) of [Pt(**4**)]BF_4

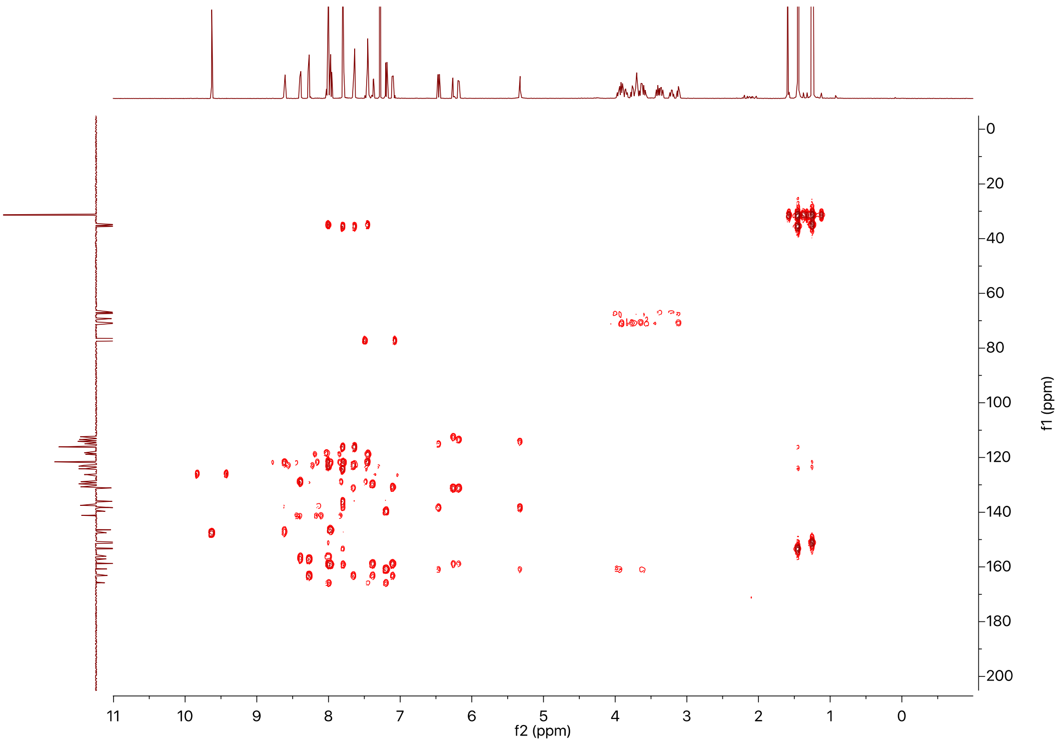

Figure S39. HMBC NMR ($CDCl_3$, 500 MHz) of [Pt(**4**)]BF_4

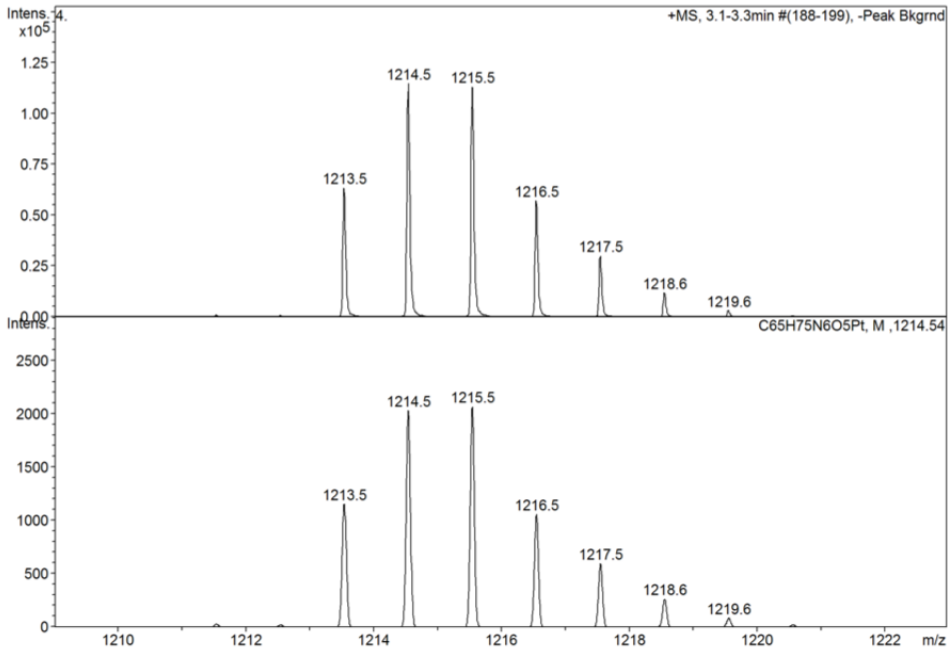

Figure S40. Isotope pattern of [Pt(**4**)]BF_4

2.8 Synthesis of [Pt(2)(5)]BF_4

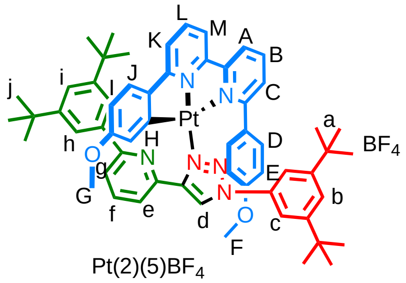

Pt(2)(5)BF_4

[Pt(**2**)(Cl)] (28 mg, 0.043 mmol, 1 eq), **5** (22 mg, 0.43 mmol, 1 eq) , $AgSbF_6$ (17.2 mg, 0.065 mmol, 1.5 eq) were mixed in CH_2Cl_2 (2 mL), heating at 60 ºC for overnight. After cooling down, the yellow suspension were diluted with CH_2Cl_2 (15 mL), and then washed with saturated NH_4BF_4 solution (20 mL), The organic phase was dried over $MgSO_4$ and the solvent was removed *in vacuo*. The crude product was purified by flash column chromatography (5% MeOH in CH_2Cl_2), affording the product as yellow oil (30 mg, 56 %). ^{1}H NMR (500 MHz, Chloroform-*d*) δ 8.96 (dd, *J* = 7.8, 0.7 Hz, 1H, H_e), 8.63 (s, 1H, H_d), 8.20 (dd, *J* = 8.1, 1.2 Hz, 1H, H_A), 8.12 (app t, *J* = 7.8 Hz, 1H, H_B), 8.02 (app t, *J* = 8.1 Hz, 1H, H_L), 7.93 (dd, *J* = 8.1, 0.9 Hz, 1H, H_M), 7.85 – 7.81 (m, 1H, H_f), 7.75 (d, *J* = 1.8 Hz, 2H, H_h), 7.70 – 7.65 (m, 4H, H_g + H_c + H_b), 7.56 (dd, *J* = 8.1, 0.9 Hz, 1H, H_K), 7.52 – 7.48 (m, 1H, H_i), 7.44 – 7.38 (m, 2H, H_C +H_J), 7.32 (br. m, 1H, one of H_E), 6.81 (br. m, 1H, one of H_E), 6.64 (dd, *J* = 8.6, 2.5 Hz, 1H, H_I), 6.53 (br. m, 1H, one of H_D), 6.38 (br. m, 1H, one of H_D), 5.72 (d, *J* = 2.5 Hz, 1H, H_H), 3.61 (s, 3H, H_F), 3.58 (s, 3H, H_G), 1.46 – 1.44 (m, 18H, H_j or H_a), 1.35 (s, 18H, H_i or H_j).^{13}C NMR (126 MHz, $CDCl_3$) δ 165.5, 162.4, 161.3, 160.7, 158.2, 157.0, 155.6, 153.7, 151.3, 149.1, 145.6, 141.1, 140.9, 138.5, 138.2, 137.9, 137.9, 135.5, 130.3, 129.5, 128.7, 126.7, 124.7, 123.8, 123.6, 122.3, 122.0, 121.6, 121.3, 120.2, 119.1, 118.9, 118.4, 115.2, 113.4, 112.6, 109.3, 55.3, 55.0, 35.4, 35.0, 31.5, 31.3. HR-MS-ESI: *m*/*z* (found) = 1085.5, *m*/*z* (calc.) = 1085.5.

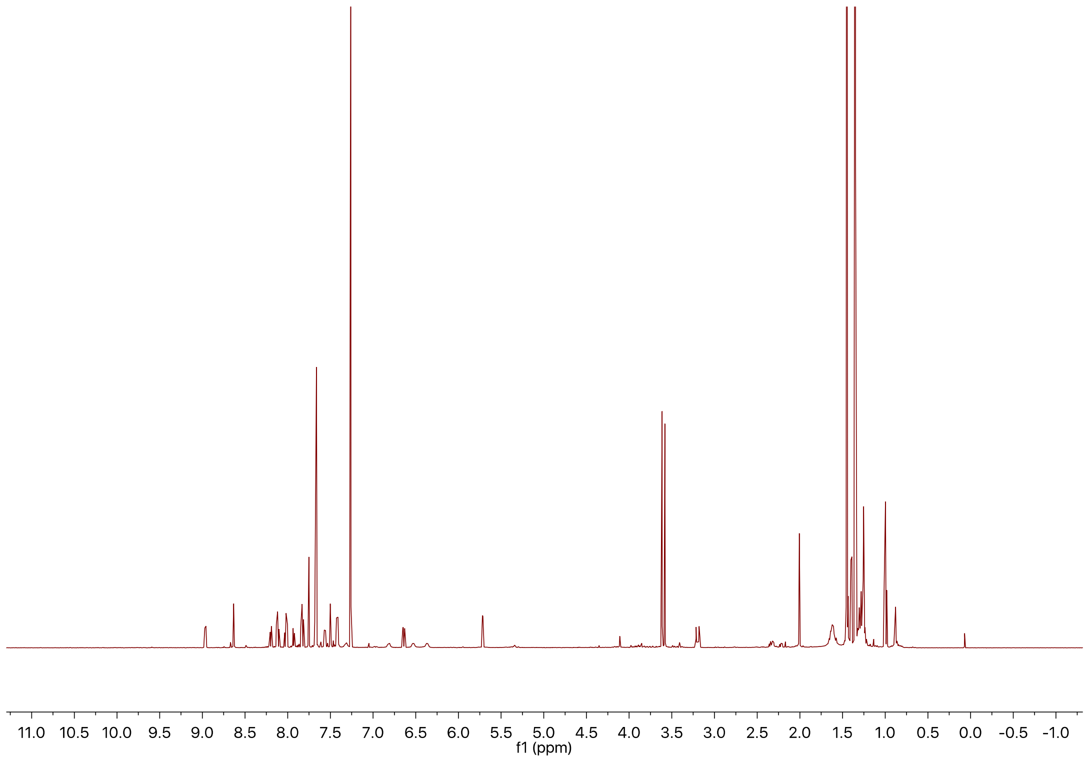

Figure S41. ^{1}H NMR ($CDCl_3$, 500 MHz) of [Pt(**2**)(**5**)]BF_4

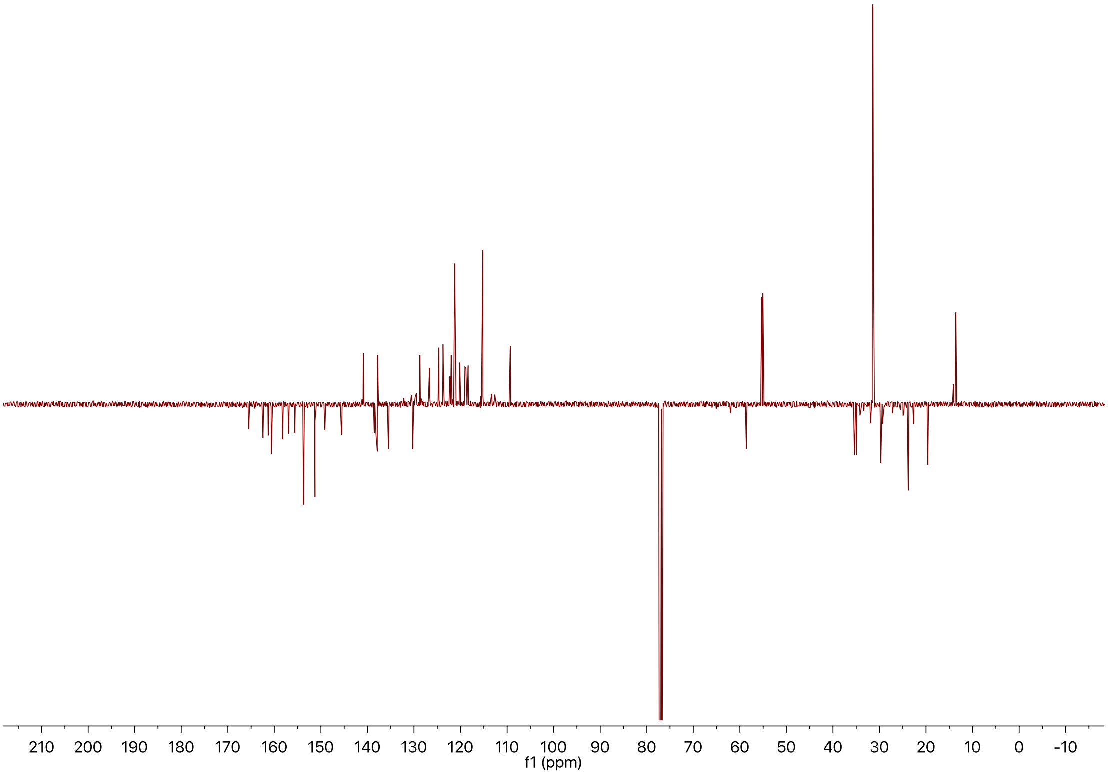

Figure S42. ^{13}C NMR ($CDCl_3$, 126 MHz) of [Pt(**2**)(**5**)]BF_4

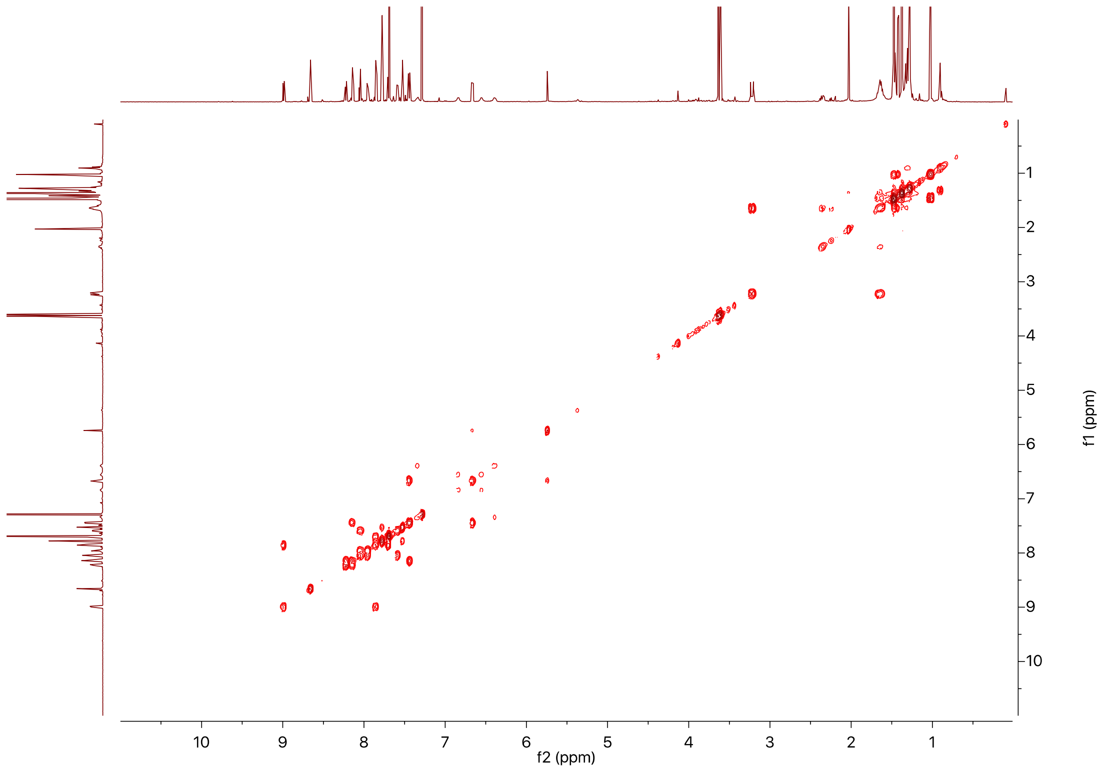

Figure S43. COSY NMR ($CDCl_3$, 500 MHz) of [Pt(**2**)(**5**)]BF_4

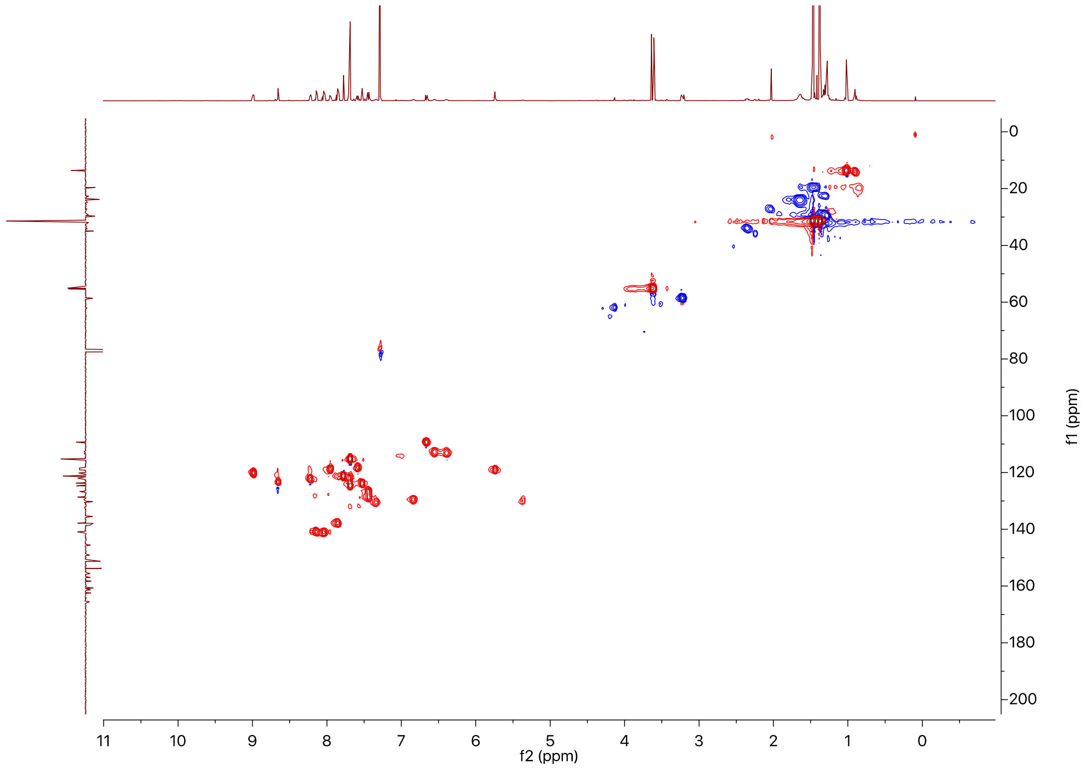

Figure S44. HSQC NMR ($CDCl_3$, 500 MHz) of [Pt(**2**)(**5**)]BF_4

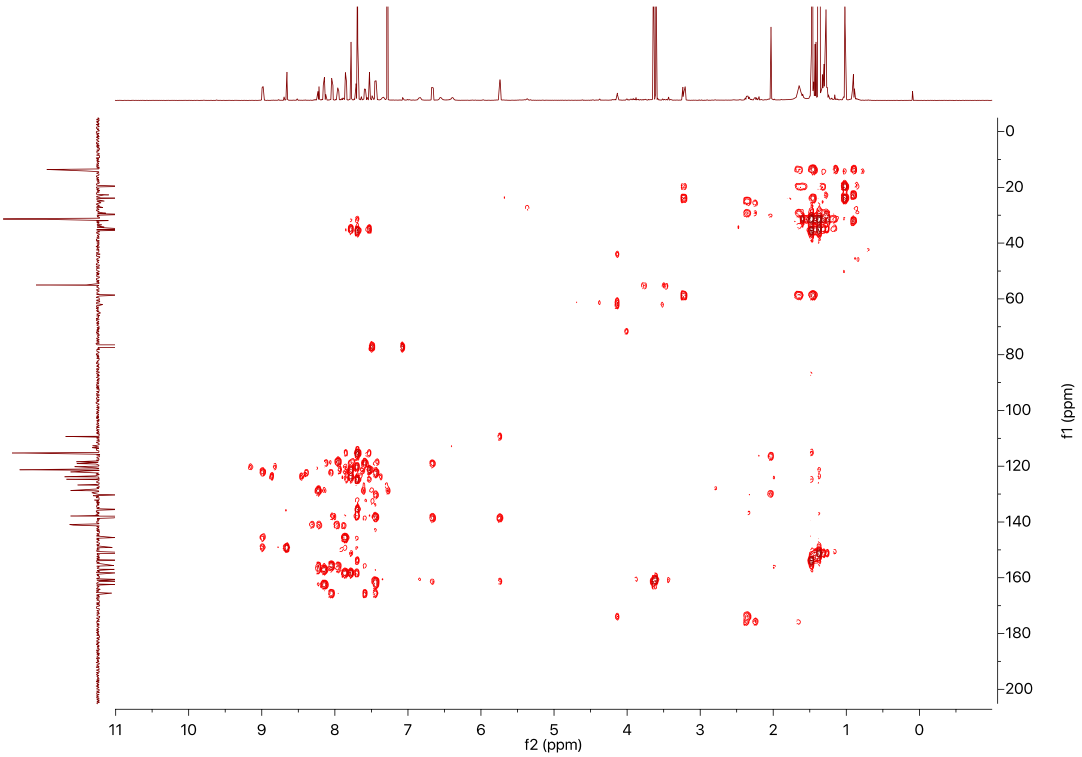

Figure S45. HMBC NMR ($CDCl_3$, 500 MHz) of [Pt(**2**)(**5**)]BF_4

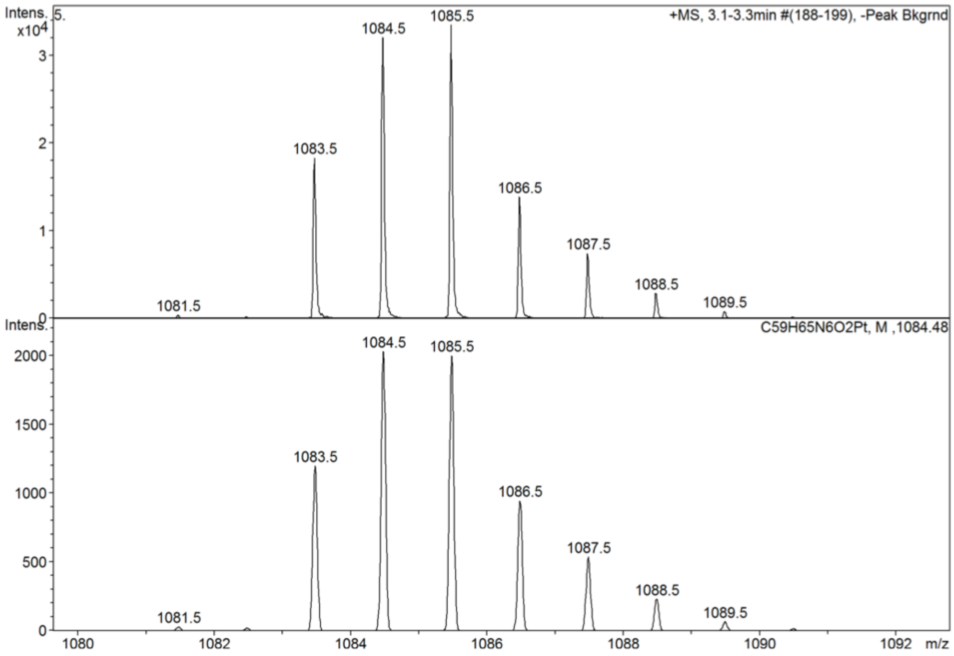

Figure S46. Isotope pattern of [Pt(**2**)(**5**)]BF_4

3 Single crystal X-ray crystallographic data

Crystals of Pt(**1**H)Cl_2(DMSO) were grown by cooling down a saturated solution of Pt(**1**H)Cl_2(DMSO) in ethyl acetate. Data for Pt(**1**H)Cl_2(DMSO) was collected on a Rigaku 007 HF diffractometer equipped with a Saturn 944+ enhanced sensitivity detector. The crystal was kept at 100(2) K during data collection. Using Olex2,[9] the structure was solved with the ShelXT[10] structure solution program using Intrinsic Phasing and refined with the ShelXL[11] refinement package using Least Squares minimisation.

Crystals of Pt(**1**)BF_4 were grown by vapour diffusion of Et_2O into MeCN solution. Data for Pt(**1**)BF_4 was collected on a FRE+ HF diffractometer equipped with a Saturn 724+ enhanced sensitivity detector. The crystal was kept at 100 K during data collection. Using Olex2,[9] the structure was solved with the ShelXT[10] structure solution program using Intrinsic Phasing and refined with the ShelXL[11] refinement package using Least Squares minimisation.

Crystals of Pt(k^4-**4**)BF_4 were grown by vapour diffusion of Et_2O into MeCN solution. Data for Pt(k^4-**4**)BF_4 was collected on a FRE+ VHF diffractometer equipped with a Saturn 724+ enhanced sensitivity detector. The crystal was kept at 100(2) K during data collection. Using Olex2, [9] the structure was solved with the ShelXT[10] structure solution program using Intrinsic Phasing and refined with the ShelXL[11] refinement package using Least Squares minimisation.

Crystals [Pt(k^5-**4**)OH]$(SbF_6)_2$ was grown by vapour diffusion of Et_2O into MeCN solution of mixed [Pt(k^4-**4**)]BF_4 with excess $AgSbF_6$ in ambient light. [Pt(k^5-**4**)OH]$(SbF_6)_2$ was collected on a Rigaku 007 HF diffractometer equipped with a Saturn 944+ enhanced sensitivity detector. The crystal was kept at 100(2) K during data collection. Using Olex2, [9] the structure was solved with the ShelXT[10] structure solution program using Intrinsic Phasing and refined with the ShelXL[11] refinement package using Least Squares minimisation.

Crystals of [Pt(k^5-**4**)OH]$(BF_4)_2$ were grown of vapour diffusion of Et_2O into MeCN solution of reaction mixture, which was obtained through oxidation of [Pt(k^4-**4**)]BF_4 by H_2O_2 followed by washing with NH_4BF_4 solution. [Pt(k^5-**4**)OH]$(BF_4)_2$ was selected on a Kat diffractometer. The crystal was kept at 100 K during data collection. Using Olex2,[9] the structure was solved with the Superflip[12] structure solution program using charge flipping and refined with the ShelXL[11] package using Least Squares minimisation.

Crystals of [Pt(k^4-**4**)Ag(MeCN)]$(SbF_6)_2$ were grown by vapour diffusion of Et_2O into MeCN solution of mixed [Pt(k^4-**4**)]BF_4 with excess $AgSbF_6$ in the dark. Data for Pt(k^4-**4**)Ag(CH_3CN)]$(SbF_6)_2$ was collected on a FRE+ VHF diffractometer equipped with a Saturn 724+ enhanced sensitivity detector. The crystal was kept at 100(2) K during data collection. Using Olex2,[9] the structure was solved with the ShelXT[10] structure solution program using Intrinsic Phasing and refined with the ShelXL[11] refinement package using Least Squares minimisation.

Crystals of [Pt(k^4-**4**)Ag(Et_2O)]$(SbF_6)_2$ were grown by vapour diffusion of Et_2O into $CHCl_3$ solution of mixed [Pt(k^4-**4**)]BF_4 with excess $AgSbF_6$ in the dark. [Pt(k^4-**4**)Ag(Et_2O)]$(SbF_6)_2$ was collected on a Rigaku 007 HF diffractometer equipped with a Saturn 944+ enhanced sensitivity detector. The crystal was kept at 100(2) K during data collection. Using Olex2, [9] the structure was solved with the ShelXT[10] structure solution program using Intrinsic Phasing and refined with the ShelXL[11] package using Least Squares minimisation.

3.1 Pt(1H)Cl_2(DMSO)

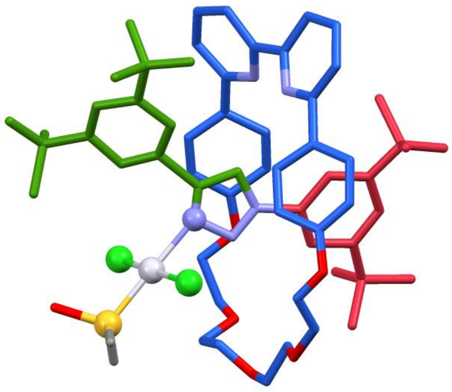

Table S1. Crystal data and structure refinement for Pt(**1**H)Cl_2(DMSO)

Identification code	Pt(**1**H)Cl_2(DMSO)
Empirical formula	$C_{62}H_{79}Cl_2N_5O_6PtS$
Formula weight	1288.35
Temperature/K	100(2)
Crystal system	monoclinic
Space group	I2/a
a/Å	26.7934(5)
b/Å	12.4321(3)
c/Å	39.1414(7)
α/°	90
β/°	106.494(2)
γ/°	90
Volume/Å^3	12501.4(5)
Z	8
ρ_{calc}g/cm^3	1.369
μ/mm^{-1}	5.706
F(000)	5296.0
Crystal size/mm^3	0.1 × 0.08 × 0.07
Radiation	CuKα (λ = 1.54178)
2Θ range for data collection/°	6.882 to 136.484
Index ranges	-31 ≤ h ≤ 23, -13 ≤ k ≤ 14, -46 ≤ l ≤ 41
Reflections collected	24565
Independent reflections	10265 [R_{int} = 0.0437, R_{sigma} = 0.0348]
Data/restraints/parameters	10265/39/776
Goodness-of-fit on F^2	1.042
Final R indexes [I>=2σ (I)]	R_1 = 0.0374, wR_2 = 0.1010
Final R indexes [all data]	R_1 = 0.0401, wR_2 = 0.1034
Largest diff. peak/hole / e Å^{-3}	1.12/-0.74

3.2 [Pt(1)]BF_4

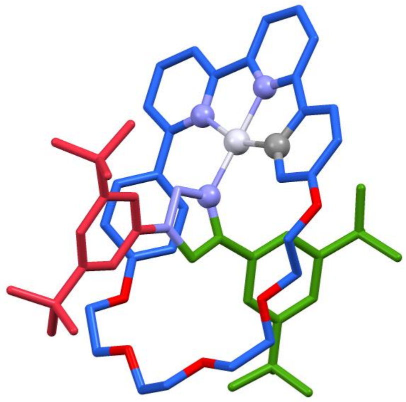

Table S2. Crystal data and structure refinement for [Pt(**1**)]BF_4

Identification code	**[Pt(1)]BF_4**
Empirical formula	$C_{60}H_{72}BF_4N_5O_5Pt$
Formula weight	1225.12
Temperature/K	100
Crystal system	triclinic
Space group	P-1
a/Å	12.22920(10)
b/Å	14.6267(2)
c/Å	18.2680(2)
α/°	81.1080(10)
β/°	76.0000(10)
γ/°	74.0800(10)
Volume/Å^3	3035.32(6)
Z	2
ρ_{calc}g/cm^3	1.340
μ/mm^{-1}	2.373
F(000)	1252.0
Crystal size/mm^3	0.2 × 0.05 × 0.02
Radiation	MoKα (λ = 0.71073)
2Θ range for data collection/°	4.616 to 64.172
Index ranges	-17 ≤ h ≤ 17, -21 ≤ k ≤ 17, -27 ≤ l ≤ 25
Reflections collected	73086
Independent reflections	19448 [R_{int} = 0.0286, R_{sigma} = 0.0261]
Data/restraints/parameters	19448/0/697
Goodness-of-fit on F^2	0.836
Final R indexes [I>=2σ (I)]	R_1 = 0.0297, wR_2 = 0.0950
Final R indexes [all data]	R_1 = 0.0329, wR_2 = 0.0983
Largest diff. peak/hole / e Å^{-3}	2.97/-0.88

3.3 Pt(k^4-4)BF_4

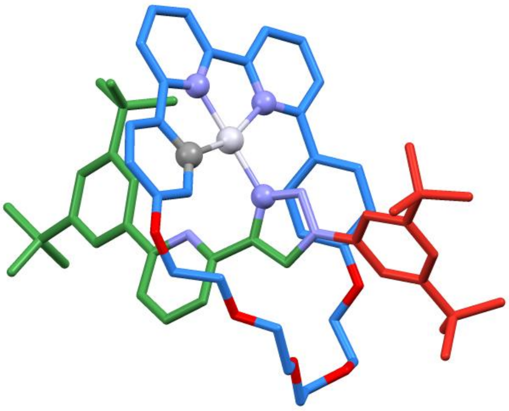

Table S3. Crystal data and structure refinement for Pt(k^4-**4**)BF_4

Identification code	**Pt(k^4-4)BF_4**
Empirical formula	$C_{66}H_{77}BF_4N_8O_5Pt$
Formula weight	1344.25
Temperature/K	100.0
Crystal system	monoclinic
Space group	$P2_1/n$
a/Å	21.7380(4)
b/Å	14.9289(3)
c/Å	21.8888(5)
α/°	90
β/°	107.531(2)
γ/°	90
Volume/Å^3	6773.5(3)
Z	4
ρ_{calc}g/cm^3	1.318
μ/mm^{-1}	2.134
F(000)	2752.0
Crystal size/mm^3	0.1 × 0.05 × 0.05
Radiation	MoKα (λ = 0.71073)
2Θ range for data collection/°	3.578 to 64.006
Index ranges	-29 ≤ h ≤ 31, -20 ≤ k ≤ 20, -29 ≤ l ≤ 31
Reflections collected	94830
Independent reflections	20137 [R_{int} = 0.0284, R_{sigma} = 0.0270]
Data/restraints/parameters	20137/4407/751
Goodness-of-fit on F^2	0.866
Final R indexes [I>=2σ (I)]	R_1 = 0.0306, wR_2 = 0.1057
Final R indexes [all data]	R_1 = 0.0412, wR_2 = 0.1134
Largest diff. peak/hole / e Å^{-3}	1.68/-1.26

3.4 [Pt(k^5-4)(OH)](SbF_6)$_2$

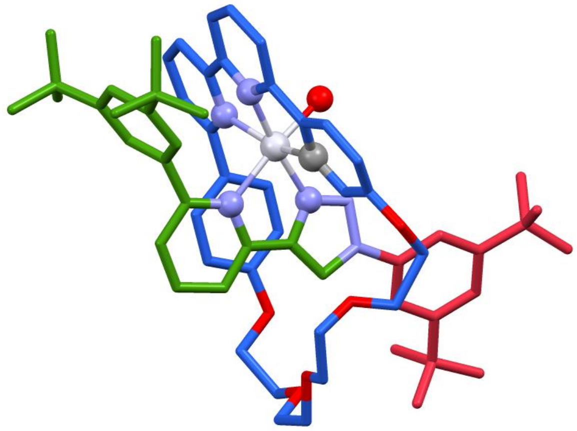

Table S4. Crystal data and structure refinement for [Pt(k^5-**4**)(OH)](SbF_6)$_2$

Identification code	PtNNC-Ag-2
Empirical formula	$C_{65}H_{76}F_6N_6O_6PtSb$
Formula weight	1468.15
Temperature/K	100(2)
Crystal system	triclinic
Space group	P-1
a/Å	11.78747(13)
b/Å	15.5279(2)
c/Å	22.4447(3)
α/°	101.2515(11)
β/°	102.1438(10)
γ/°	103.7234(10)
Volume/Å^3	3769.48(9)
Z	2
ρ_{calc}g/cm^3	1.294
μ/mm^{-1}	2.273
F(000)	1478.0
Crystal size/mm^3	0.05 × 0.04 × 0.03
Radiation	MoKα (λ = 0.71073)
2Θ range for data collection/°	3.676 to 61.62
Index ranges	-16 ≤ h ≤ 15, -22 ≤ k ≤ 22, -31 ≤ l ≤ 30
Reflections collected	75959
Independent reflections	21637 [R_{int} = 0.0285, R_{sigma} = 0.0253]
Data/restraints/parameters	21637/103/833
Goodness-of-fit on F^2	1.053
Final R indexes [I>=2σ (I)]	R_1 = 0.0410, wR_2 = 0.1157
Final R indexes [all data]	R_1 = 0.0443, wR_2 = 0.1182
Largest diff. peak/hole / e Å^{-3}	1.61/-2.88

3.5 [Pt(k^5-4)OH]$(BF_4)_2$

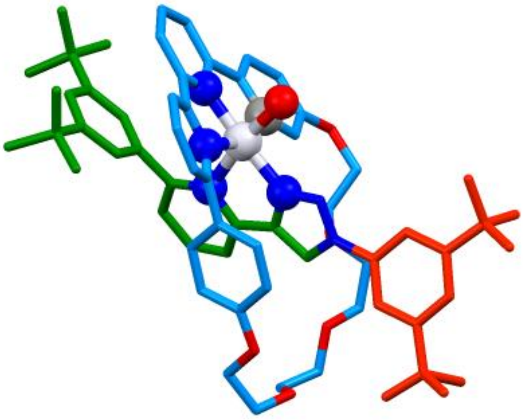

Table S5 Crystal data and structure refinement for [Pt(k^5-**4**)OH]$(BF_4)_2$

Identification code	[Pt(k^5-**4**)OH]$(BF_4)_2$
Empirical formula	$B_2C_{65}F_8N_6O_6PtH65$
Formula weight	1329.92
Temperature/K	100(2)
Crystal system	triclinic
Space group	P-1
a/Å	10.1984(4)
b/Å	18.2072(7)
c/Å	19.7789(7)
α/°	105.686(3)
β/°	99.655(3)
γ/°	96.115(3)
Volume/Å^3	3440.9(2)
Z	2
ρ_{calc}g/cm^3	1.284
μ/mm^{-1}	2.109
F(000)	1281.0
Crystal size/mm^3	0.05 × 0.04 × 0.02
Radiation	MoKα (λ = 0.71073)
2Θ range for data collection/°	5.744 to 59.75
Index ranges	-12 ≤ h ≤ 14, -24 ≤ k ≤ 24, -27 ≤ l ≤ 27
Reflections collected	81332
Independent reflections	18175 [R_{int} = 0.2849, R_{sigma} = 0.2676]
Data/restraints/parameters	18175/746/806
Goodness-of-fit on F^2	0.985
Final R indexes [I>=2σ (I)]	R_1 = 0.1139, wR_2 = 0.2185
Final R indexes [all data]	R_1 = 0.1889, wR_2 = 0.2575
Largest diff. peak/hole / e Å^{-3}	4.11/-1.66

3.6 [Pt(k^4-4)Ag(CH_3CN)](SbF_6)$_2$

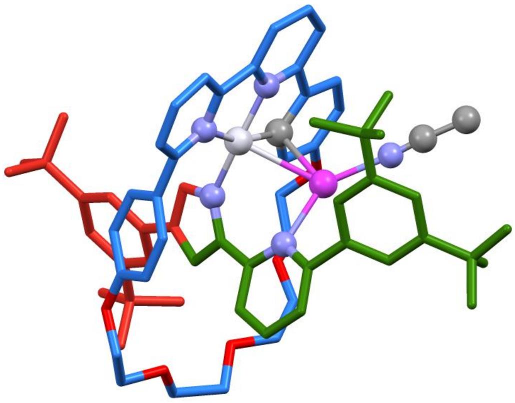

Table S6. Crystal data and structure refinement for [Pt(**4**)Ag(CH_3CN)](SbF_6)$_2$

Identification code	**[Pt(4)Ag(CH_3CN)](SbF_6)$_2$**
Empirical formula	$C_{67}H_{77}AgF_{15}N_7O_5PtSb_{2.5}$
Formula weight	1952.69
Temperature/K	100(2)
Crystal system	triclinic
Space group	P-1
a/Å	13.0371(5)
b/Å	18.3984(8)
c/Å	18.4639(8)
α/°	88.810(3)
β/°	89.789(3)
γ/°	83.438(3)
Volume/Å^3	4398.8(3)
Z	2
ρ_{calc}g/cm^3	1.474
μ/mm^{-1}	2.637
F(000)	1911.0
Crystal size/mm^3	0.3 × 0.2 × 0.08
Radiation	MoKα (λ = 0.71073)
2Θ range for data collection/°	4.232 to 50.054
Index ranges	-15 ≤ h ≤ 15, -21 ≤ k ≤ 21, -21 ≤ l ≤ 21
Reflections collected	85505
Independent reflections	15515 [R_{int} = 0.0727, R_{sigma} = 0.0510]
Data/restraints/parameters	15515/1177/940
Goodness-of-fit on F^2	1.040
Final R indexes [I>=2σ (I)]	R_1 = 0.0651, wR_2 = 0.1632
Final R indexes [all data]	R_1 = 0.0804, wR_2 = 0.1722
Largest diff. peak/hole / e Å^{-3}	6.97/-2.47

3.7 [Pt(k^4-4)Ag(Et_2O)](SbF_6)$_2$

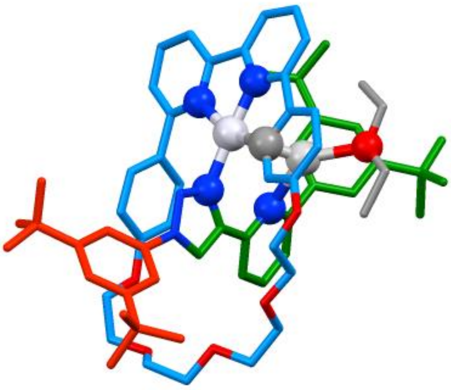

Table S7 Crystal data and structure refinement for [Pt(k^4-**4**)Ag(Et_2O)](SbF_6)$_2$.

Identification code	[Pt(k^4-**4**)Ag(Et_2O)](SbF_6)$_2$
Empirical formula	C69H85AgF12N6O6PtSb2
Formula weight	1868.88
Temperature/K	100(2)
Crystal system	monoclinic
Space group	P21/n
a/Å	18.7462(4)
b/Å	15.4022(3)
c/Å	27.6759(5)
α/°	90
β/°	104.791(2)
γ/°	90
Volume/Å3	7726.1(3)
Z	4
ρcalcg/cm3	1.607
μ/mm-1	11.478
F(000)	3696.0
Crystal size/mm3	0.2 × 0.1 × 0.03
Radiation	CuKα (λ = 1.54178)
2Θ range for data collection/°	5.144 to 140.134
Index ranges	-22 ≤ h ≤ 22, -15 ≤ k ≤ 18, -33 ≤ l ≤ 33
Reflections collected	84286
Independent reflections	14409 [Rint = 0.1181, Rsigma = 0.0617]
Data/restraints/parameters	14409/39/916
Goodness-of-fit on F2	1.039
Final R indexes [I>=2σ (I)]	R1 = 0.0676, wR2 = 0.1755
Final R indexes [all data]	R1 = 0.0984, wR2 = 0.1971
Largest diff. peak/hole / e Å-3	2.33/-2.15

4 Stability of Pt(II) complex

4.1 Slow decomposition of complex [Pt(2)(3)]BF_4 in $CDCl_3$

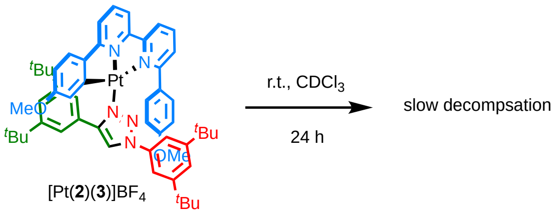

Scheme S1 Slow decomposition of complex [Pt(**2**)(**3**)]BF_4 in $CDCl_3$

The non-interlocked complexes [Pt(**2**)(**3**)]BF_4 was found to slowly decompose in $CDCl_3$ at room temperature (**Figure S47**). Leaving complex [Pt(**2**)(**3**)]BF_4 in $CDCl_3$ at a concentration of 2.5 mM at room temperature for 24 h led to the formation of small portion of axle **3**.

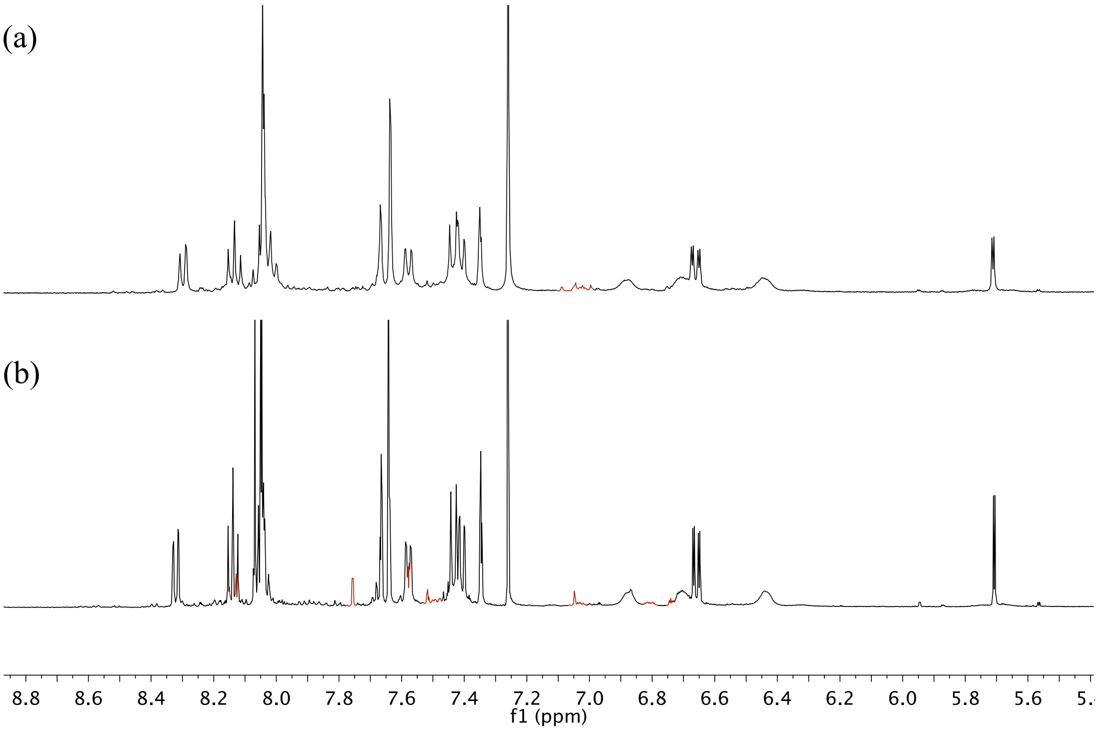

Figure S47. Partial ^{1}H NMR ($CDCl_3$, 400 MHz, 298 K) of (a) complex [Pt(**2**)(**3**)]BF_4; (b) complex [Pt(**2**)(**3**)]BF_4 after 24 h in $CDCl_3$.

4.2 Slow decomposition of complex [Pt(2)(5)]BF_4 in $CDCl_3$

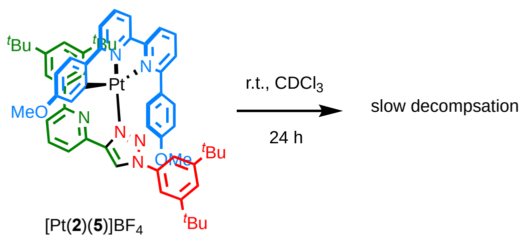

Scheme S2. Slow decomposition of complex [Pt(**2**)(**5**)]BF_4 in $CDCl_3$

Leaving complex [Pt(**2**)(**5**)]BF_4 at a concentration of 2.5 mM in $CDCl_3$ at room temperature for 24 h led to the formation of a small amount of Pt(**2**)Cl and axle **5** (**Figure S48**).

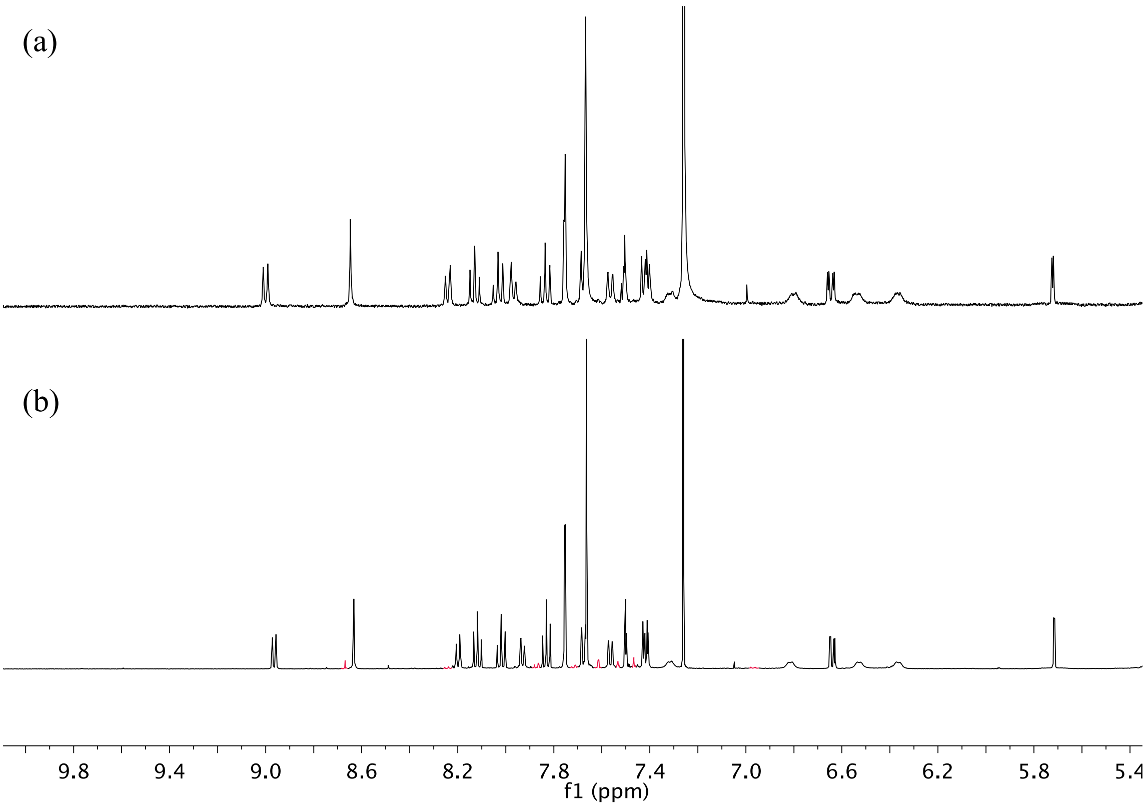

Figure S48. Partial ^{1}H NMR ($CDCl_3$, 400 MHz, 298 K) of (a) complex [Pt(**2**)(**5**)]BF_4; (b) complex [Pt(**2**)(**5**)]BF_4 after 24 h in $CDCl_3$.

4.3 Ligand substitution of [Pt(2)(3)]BF$_4$

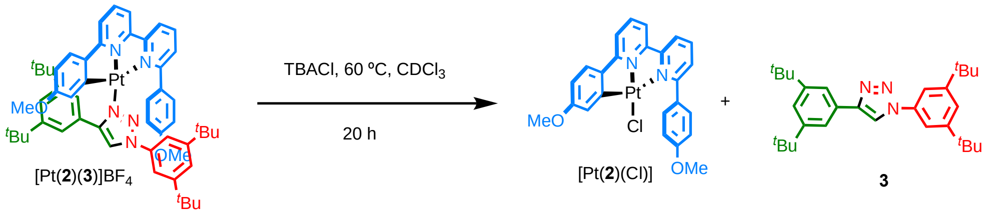

Scheme S3. Demonstration of ligand substitution of complex [Pt(**2**)(**3**)]BF$_4$ in the presence of TBACl at 60 ºC

As shown in **Scheme S3**, the non-interlocked complex [Pt(**2**)(**3**)]BF$_4$ decomposed to [Pt(**2**)]Cl and axle **3** in the presence of 2 equivalents of TBACl in CDCl$_3$ at 60 ºC, and reached completion after 20 h (**Figure S49**).

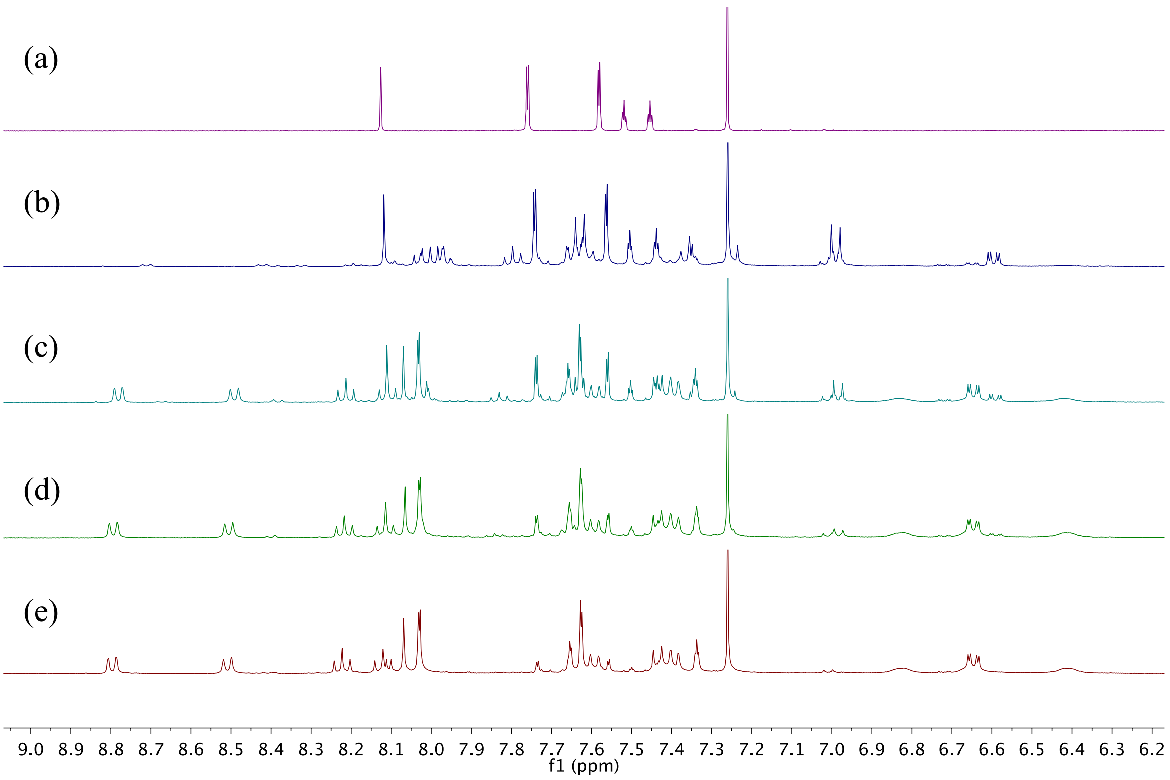

Figure S49. Partial ^{1}H NMR (CDCl$_3$, 400 MHz, 298 K) of (a) axle **3**; and the reaction of [Pt(**2**)(**3**)]BF$_4$ with TBACl after (b) 20 h; (c) 3.5 h; (d) 0.5 h; (e) complex [Pt(**2**)(**3**)]BF$_4$

4.4 Ligand substitution of [Pt(2)(5)]BF4

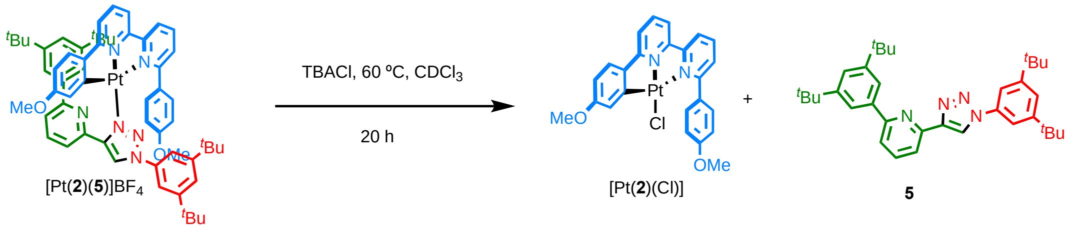

Scheme S4. Ligand substitution of complex [Pt(**2**)(**5**)]BF_4 by TBACl at 60 ºC

The non-interlocked complex [Pt(**2**)(**5**)]BF_4 was also found to decompose to [Pt(**2**)]Cl and axle **5** in the presence of 2 equivalents of TBACl in $CDCl_3$ at 60 ºC, and reached completion after 20 h (**Figure S50**).

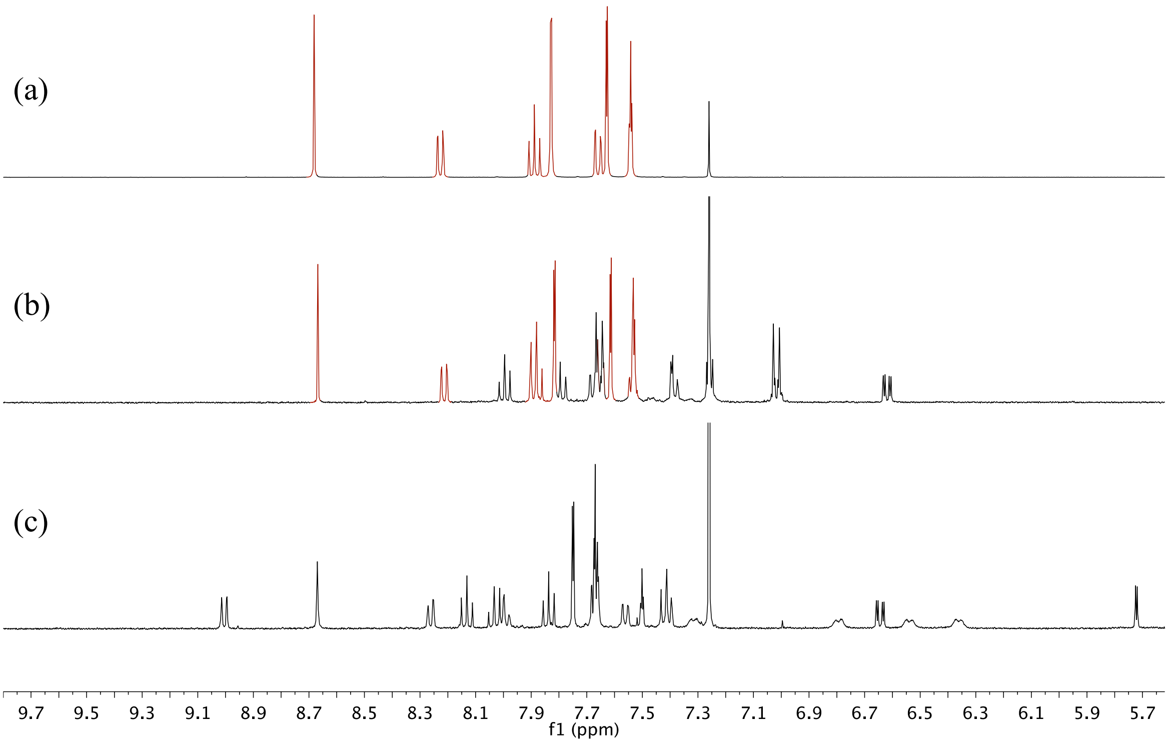

Figure S50. Partial ^1H NMR ($CDCl_3$, 400 MHz, 298 K) of (a) axle **5**; (b) mixture of [Pt(**2**)(**5**)]BF_4 and TBACl after 20 h; (c) complex [Pt(**2**)(**5**)]BF_4

4.5 Ligand substitution of [Pt(1)]BF_4

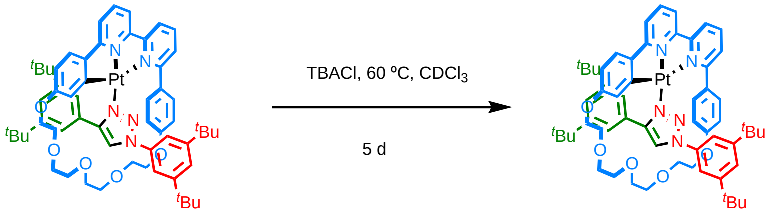

Scheme S5. Ligand substitution of complex [Pt(**1**)]BF_4 in the presence of TBACl at 60 ºC

The interlocked complex [Pt(**1**)]BF_4 remained intact in the presence of 2 equiv. of TBACl under same conditions as no decomposition was observed, even after a period of 5 days (**Figure S51**).

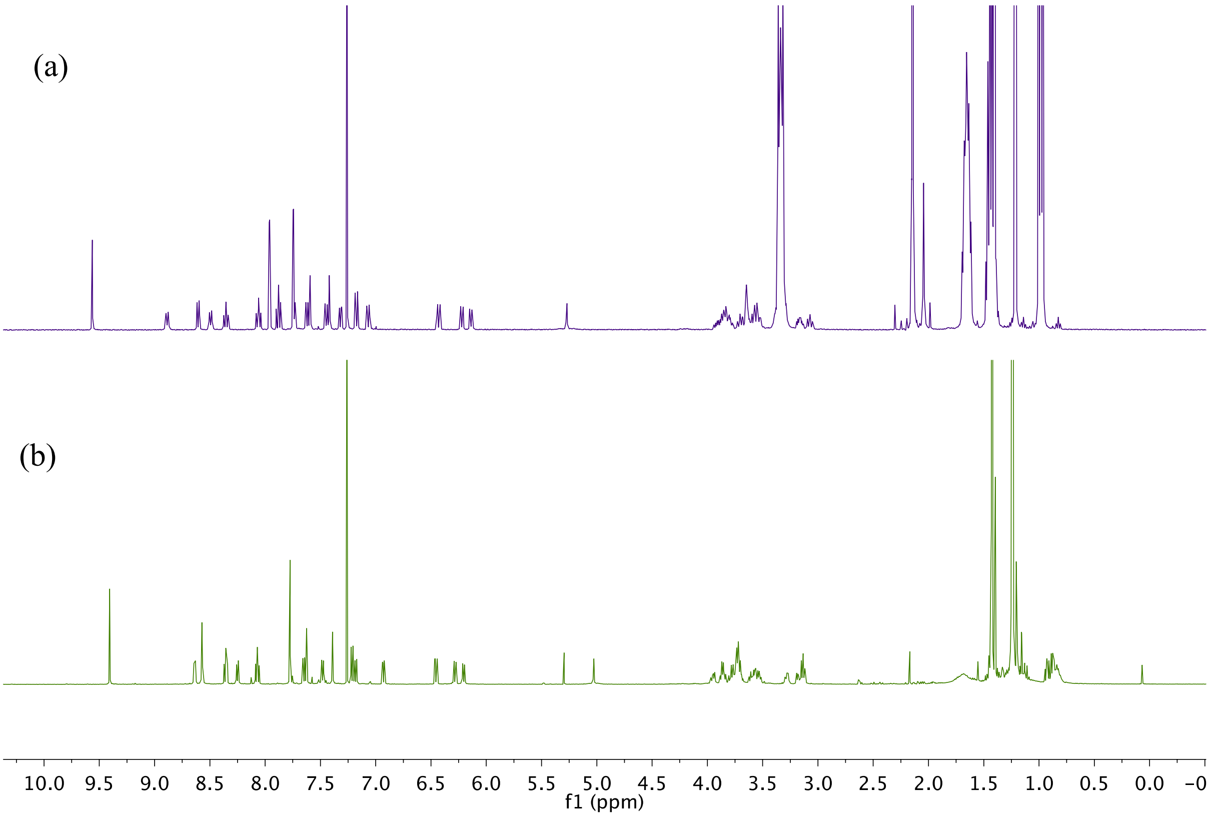

Figure S51. Partial ^{1}H NMR ($CDCl_3$, 400 MHz, 298 K) of (a) mixture of [Pt(**1**)]BF_4 and TBACl after 5 d; (b) complex [Pt(**1**)]BF_4

4.6 Ligand substitution of [Pt(k^4-4)]BF_4

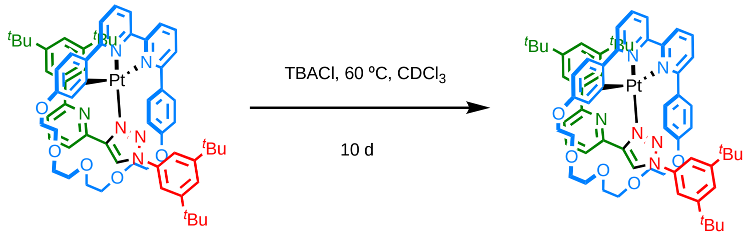

Scheme S6 Ligand substitution of complex [Pt(k⁴-**4**)]BF_4 by TBACl at 60 ºC

Similar for complex [Pt(**1**)]BF_4, no decomposition of [Pt(k^4-**4**)]BF_4 was observed after 5 days stirring at 60 ºC in the presence of 2 equiv. TBACl.

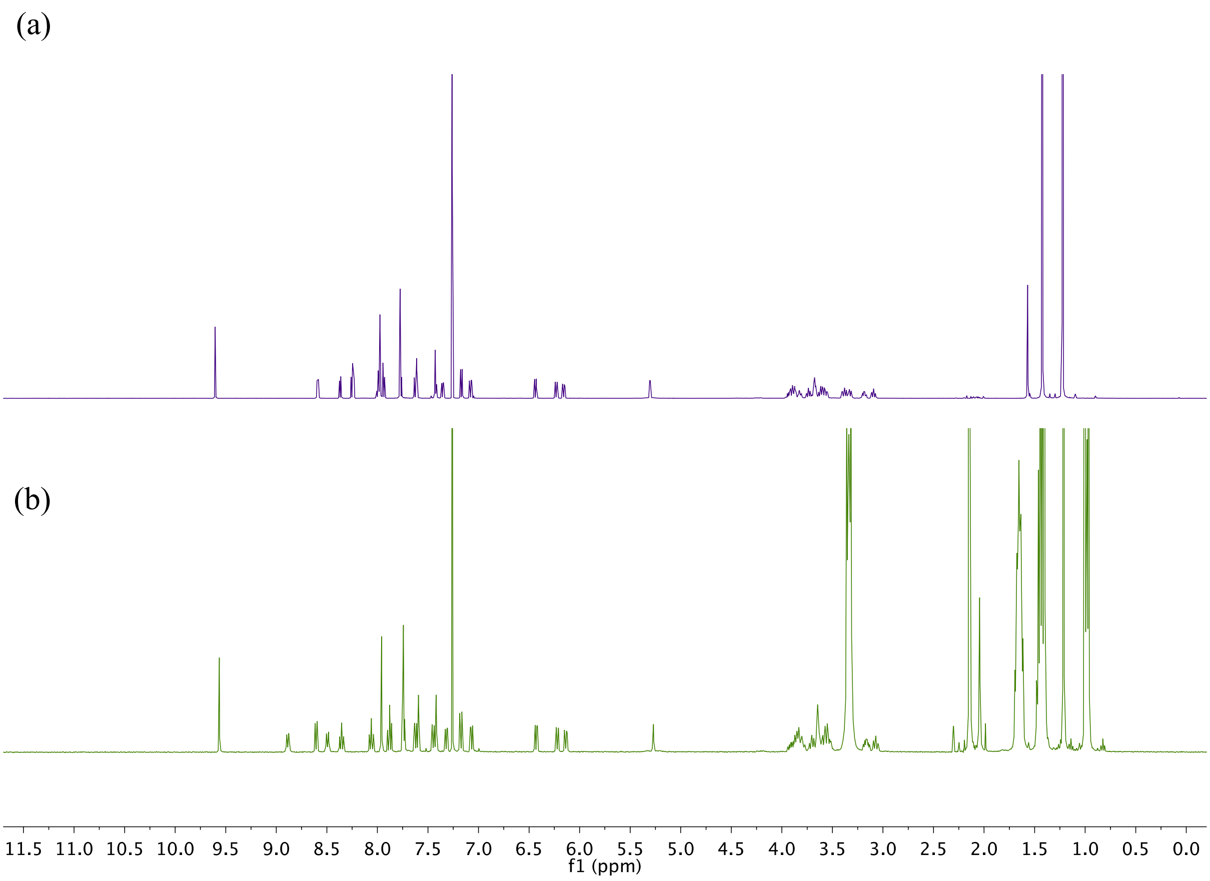

Figure S52. Partial ^1H NMR ($CDCl_3$, 400 MHz, 298 K) of (a) complex [Pt(k^4-**4**)]BF_4; (b) mixture of [Pt(k^4-**4**)]BF_4 and TBACl after 5 d

4.7 Oxidation of [Pt(1)]BF4 with *tert*-butyl hydroperoxide

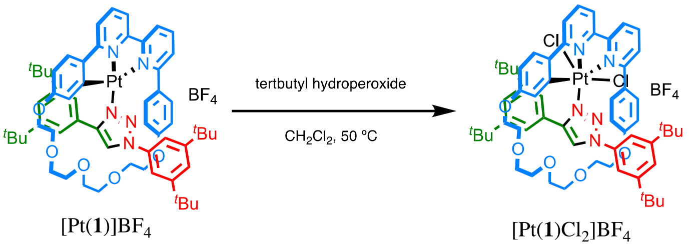

Scheme S7. Oxidation of complex [Pt(**1**)]BF4

Pt(**1**)BF4 (18.2 mg, 0.015 mmol, 1 eq), $^{t}BuO_2H$ (5-6 M in decane, 30 uL, 1.5 mmol, 100 eq) were mixed in CH_2Cl_2 (1.5 mL) and heating at 50 ºC. Aliquots (0.4 mL) were withdrawn, diluted with CH_2Cl_2 then washed with NH_4BF_4. The organic phase was dried over $MgSO_4$ and the solvent was removed *in vacuo*. The reaction mixture was then analysed by ^1H NMR and ESI-LRMS.

Full conversion of complex [Pt(**1**)]BF4 was observed after 20 h reaction time to give a single major product by ^1H NMR (**Figure S53**). ESI-LRMS results, suggests the formation of [Pt(**1**)Cl2]BF4 (*m/z* = 1208.5) in which two Cl^- are coordinating to Pt(IV) as additional ligands, presumably through the oxidation of CH_2Cl_2. Unfortunately, we were unable to prepare crystals suitable for SCXRD to confirm the identity of the product.

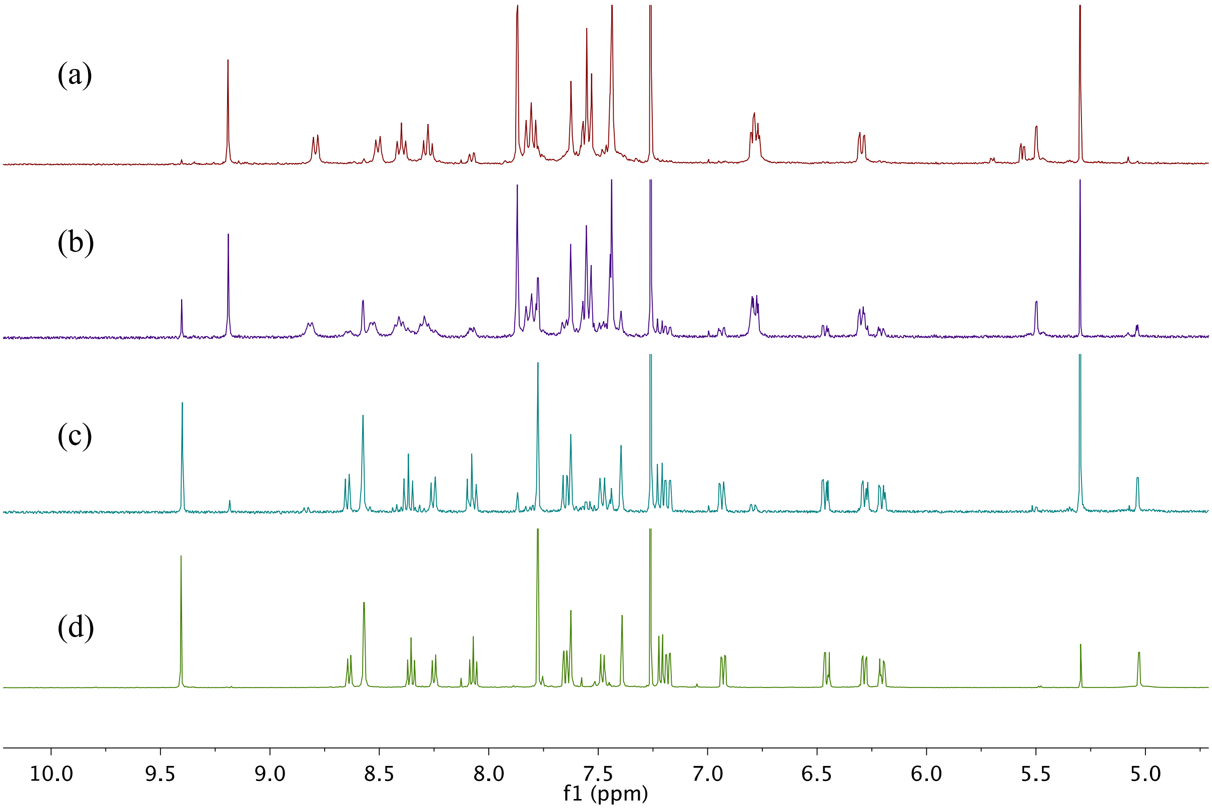

Figure S53. Partial ^1H NMR ($CDCl_3$, 400 MHz, 298 K) of reaction (a) monitored after 36 h after NH_4BF_4 work up; (b) monitored after 18 h after NH_4BF_4 work up; (c) monitored after 4 h after NH_4BF_4 work up; (d) complex [Pt(1)]BF4

4.8 Oxidation of [Pt(2)(3)]BF_4 with *tert*-butyl hydroperoxide

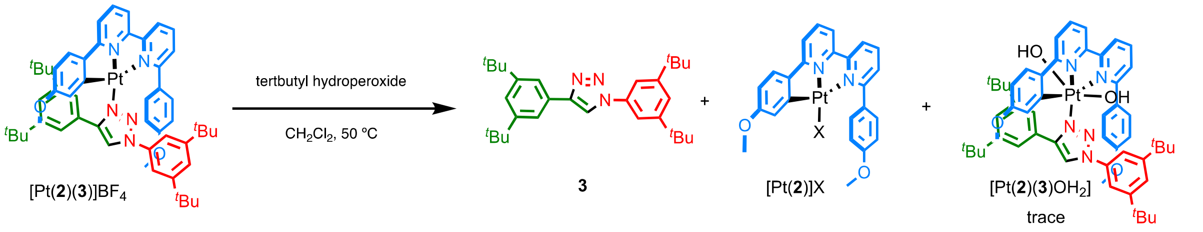

Scheme S8. Oxidation of complex [Pt(**2**)(**3**)]BF_4

[Pt(**2**)(**3**)]BF_4 (10 mg, 0.009 mmol, 1 eq), $^tBuO_2H$ (5-6 M, 18 uL, 0.9 mmol, 100 eq) were mixed in CH_2Cl_2 (0.7 mL) and the mixture heated at 50 ºC. Aliquots (0.4 mL) were withdrawn, diluted with CH_2Cl_2 then washed with NH_4BF_4. The organic phase was dried over $MgSO_4$ and the solvent was removed *in vacuo*. The reaction mixture was then analysed by ^1H NMR and LR-MS.

^1H NMR analysis revealed formation of axle **3** along with trace amount of new species in only 4 h (**Figure S54**). ESI-LRMS suggests the minor new species could be oxidised product [Pt(**2**)(**3**)$(OH)_2$] (m/z = 1042.6). However, the high ratio of axle **3** formation over new species in the mixture suggests significant decomposition under these conditions due to the instability of the Pt-triazole bond.

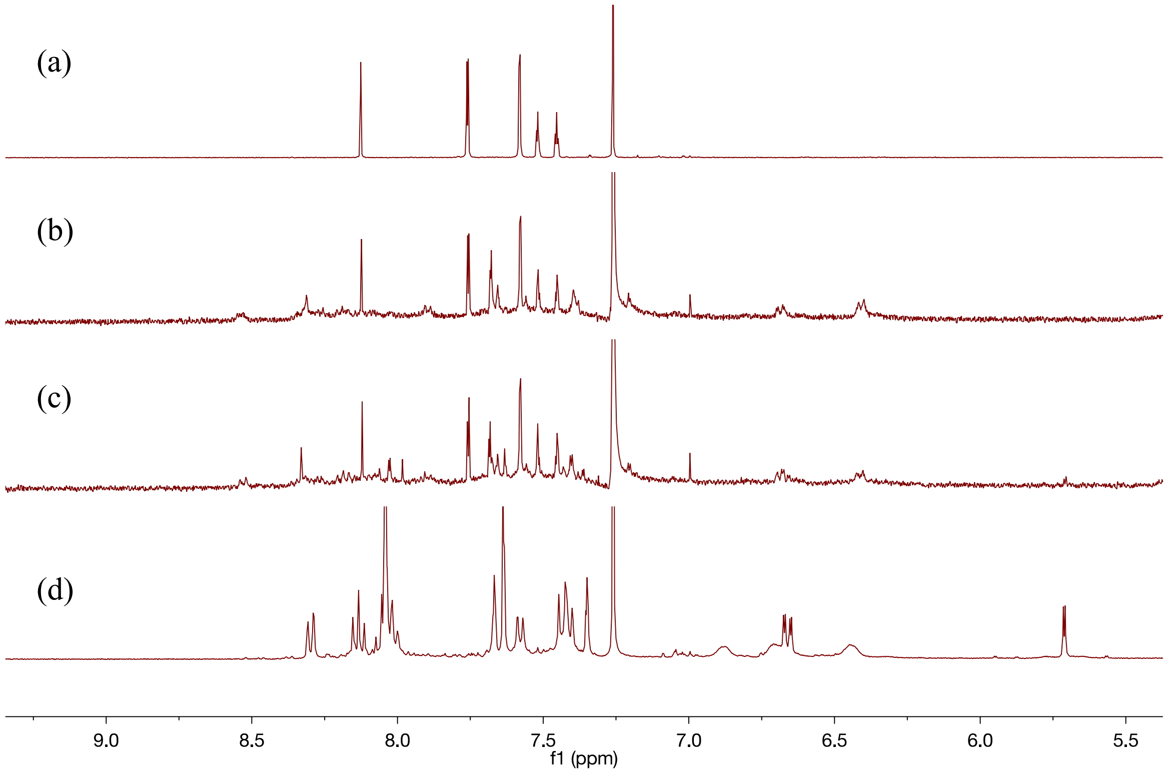

Figure S54. Partial ^1H NMR ($CDCl_3$, 400 MHz, 298 K) of (a) axle **3**; (b) reaction of complex [Pt(**2**)(**3**)]BF_4 with after overnight; (c) aliquot after 4 h; (d) complex [Pt(**2**)(**3**)]BF_4

4.9 Oxidation of [Pt(*k*⁴-4)]BF_4 with *tert*-butyl hydroperoxide

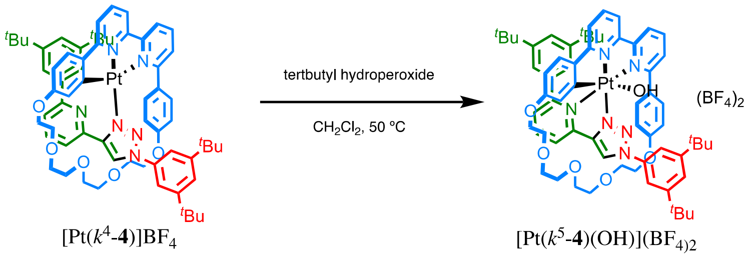

Scheme S9. Oxidation of complex [Pt(k⁴-**4**)]BF_4

Pt(k^4-**4**)BF_4 (20 mg, 0.015 mmol, 1 eq), ${}^tBuO_2H$ (5-6 M, 30 uL, 1.5 mmol, 100 eq) were mixed in CH_2Cl_2 (1.5 mL), heating at 50 ºC. The aliquot samples (0.4 mL) were diluted with CH_2Cl_2 then washed with NH_4BF_4. The organic phase was dried over $MgSO_4$ and the solvent was removed *in vacuo*. The reaction mixture was then analysed by ${}^1H$ NMR and LR-MS.

${}^1H$ NMR reveals full conversion of starting material after 2 h reaction time to give a single major product, although additional species are also present (**Figure S55**). ESI-LRMS suggests the formation of [Pt(k^5-**4**)(OH)]$(BF_4)_2$ (m/z = 616.3). An authentic sample of this compound was obtained by oxidation with Ag(I) under ambient light and characterised by SCXRD (**Table S4**). Alternatively, the same compound was obtained by crystallisation of the product of the reaction with H_2O_2 (**Table S5**).

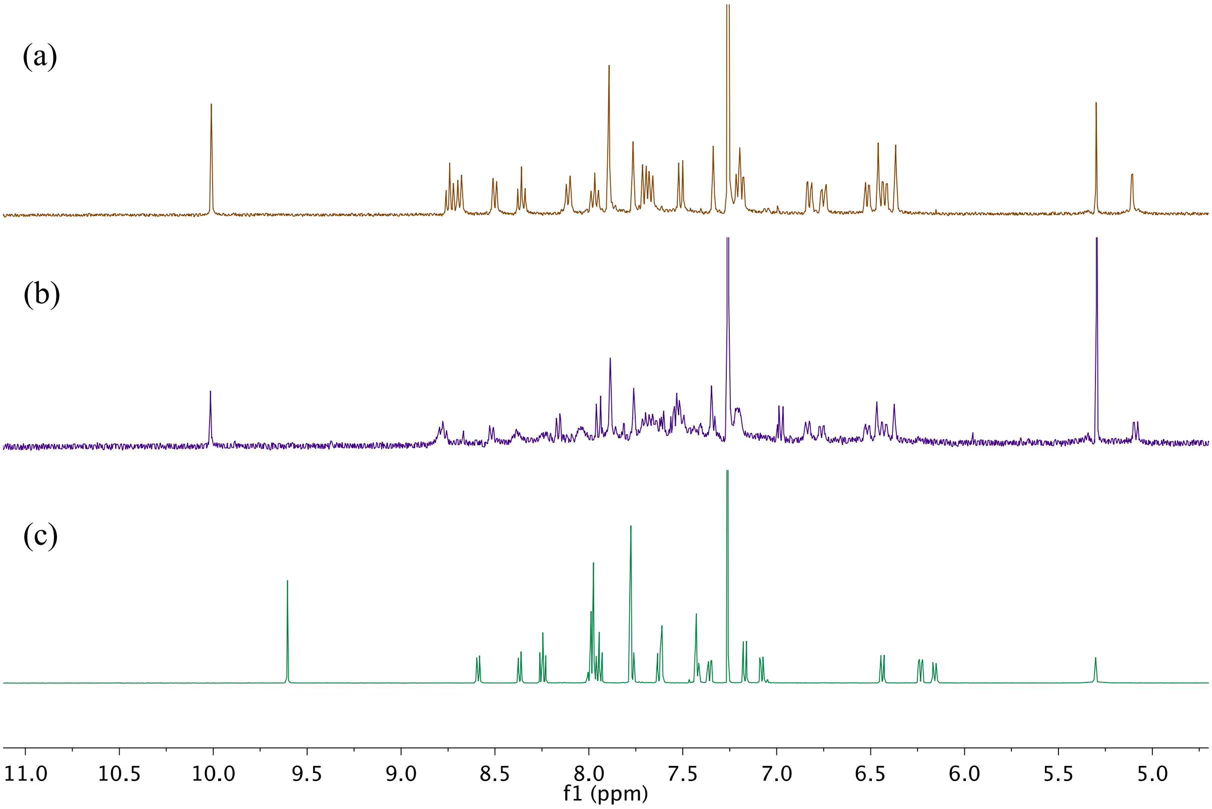

Figure S55. Partial ${}^1H$ NMR ($CDCl_3$, 400 MHz, 298 K) of (a) [Pt(k⁵-**4**)(OH)]$(BF_4)_2$ obtained by oxidation with H_2O_2. (b) product of reaction of [Pt(k⁴-**4**)]BF_4 with ${}^tBuO_2H$ for 2 h followed by NH_4BF_4 wash; (c) complex [Pt(k⁴-**4**)]BF_4.

4.10 Oxidation of [Pt(k^4-4)]BF_4 with H_2O_2

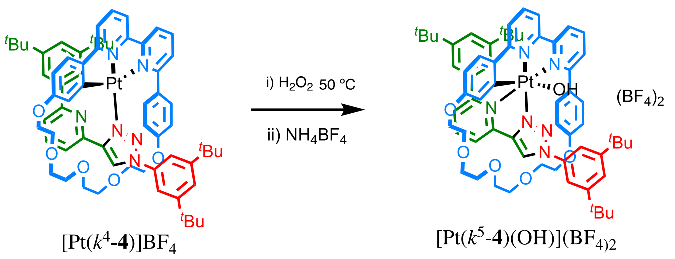

Scheme S10. Oxidation of complex [Pt(k4-**4**)]BF_4

Pt(k^4-**4**)BF_4 (30 mg, 0.022 mmol) was heated in H_2O_2 (30% w/w in H_2O, 4 mL) at 50 º. Aliquots were withdrawn (0.5 mL), diluted with CH_2Cl_2 then washed with NH_4BF_4. The organic phase was dried over $MgSO_4$ and the solvent was removed *in vacuo*. The reaction mixture was then analysed by ^1H NMR and LR-MS.

^1H NMR analysis revealed clean conversion of [Pt(k^4-**4**)]BF_4 to [Pt(k^5-**4**)(OH)]$(BF_4)_2$ over 20 h with no dissociation observed (**Figure S56**). ESI-LRMS supported this interpretation (*m*/*z* = 616.3)). Crystals suitable for SCXRD (**Table S5**) confirmed the formation of the Pt(IV) species.

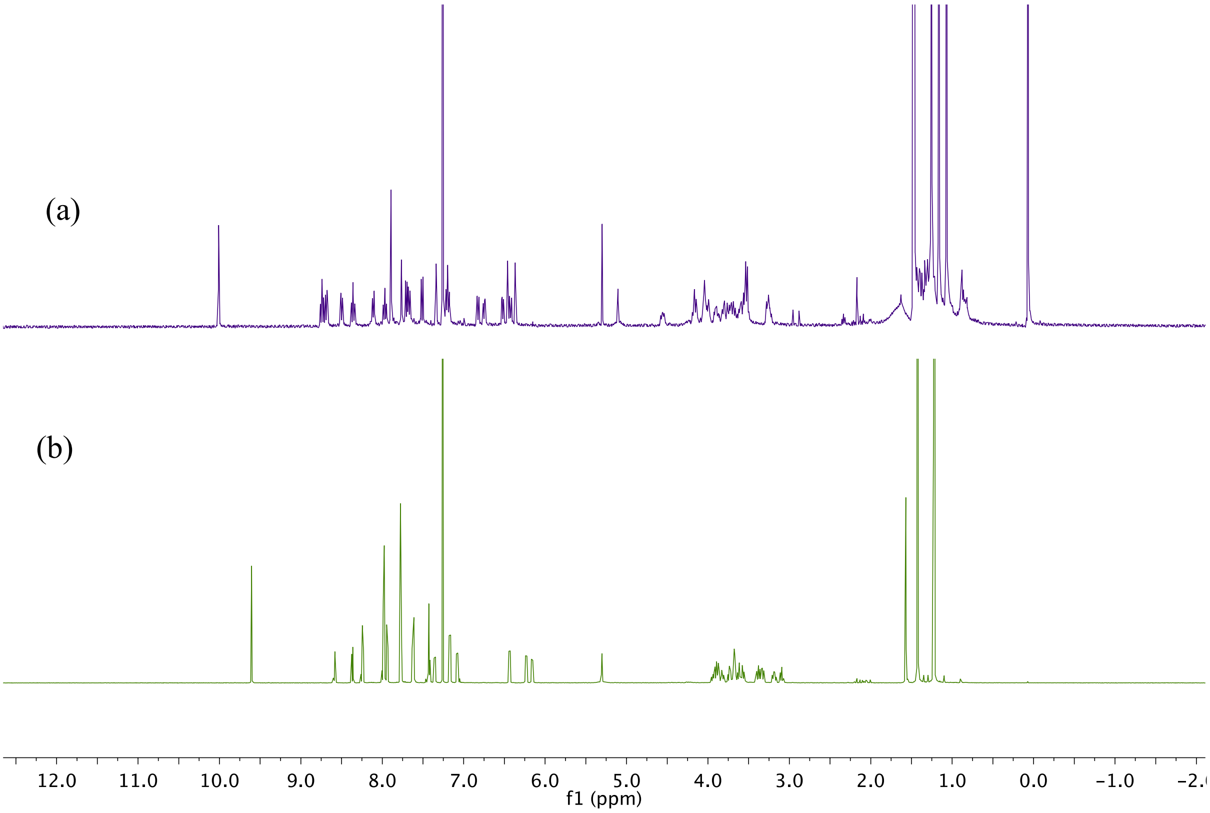

Figure S56. Partial ^1H NMR ($CDCl_3$, 400 MHz, 298 K) of (a) product obtained after reacting complex [Pt(k4-**4**)]BF_4 with H_2O_2 for 20 h followed by NH_4BF_4 wash; (b) complex [Pt(k4-**4**)]BF_4.

4.11 Oxidation of [Pt(2)(5)]BF_4 with *tert*-butyl hydroperoxide

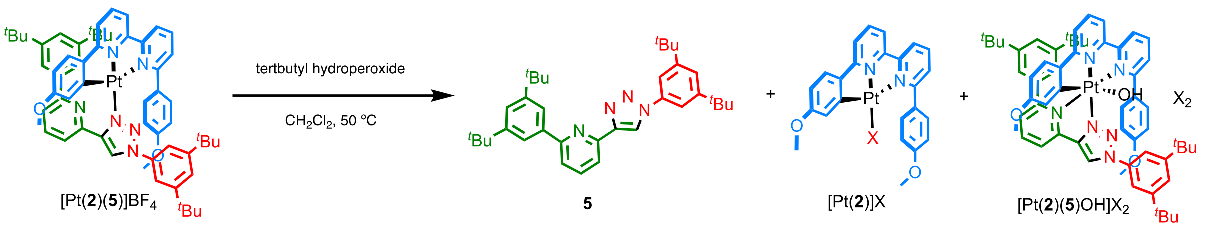

Scheme S11. Oxidation of complex [Pt(**2**)(**5**)]BF_4

[Pt(**2**)(**5**)]BF_4 (19 mg, 0.016 mmol, 1 eq), $^tBuO_2H$ (5-6 M, 30 uL, 1.6 mmol, 100 eq) were mixed in CH_2Cl_2 (1.5 mL) and heated at 50 ºC. Aliquots (0.4 mL) were withdrawn, diluted with CH_2Cl_2 then washed with NH_4BF_4. The organic phase was dried over $MgSO_4$ and the solvent was removed *in vacuo*. The aliquots were analysed by ^1H NMR and LR-MS.

^1H NMR (**Figure S57**) indicated complete consumption of [Pt(**2**)(**5**)]BF_4 after 20 h with small amounts of axle **5** observed in addition to a several new Pt species. ESI-LRMS indicated the formation of [Pt(**2**)(**5**)(OH)]X_2 (m/z = 551.2), although this new species could not be purified.

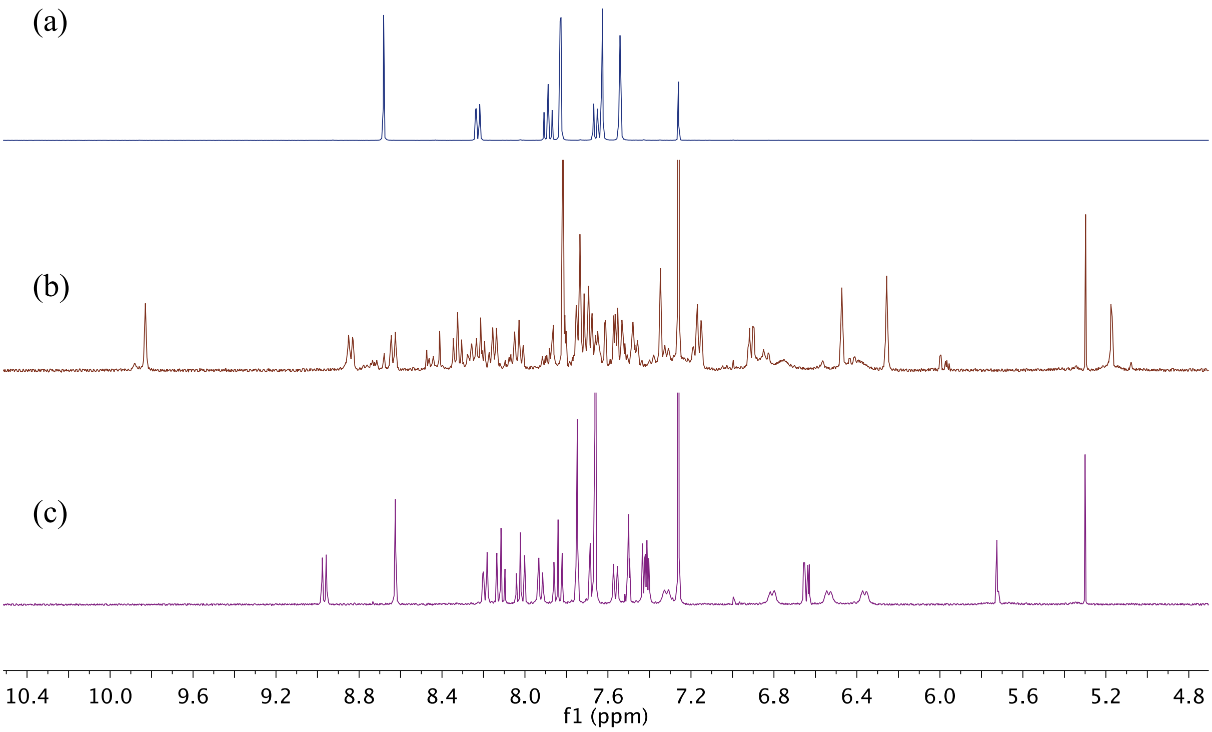

Figure S57. Partial ^1H NMR ($CDCl_3$, 400 MHz, 298 K) of (a) axle **5**; (b) product mixture obtained by reacting [Pt(**2**)(**5**)]BF_4 with tert-butyl hydroperoxide over 20 h after NH_4BF_4 work up; (c) complex [Pt(**2**)(**5**)]BF_4.

4.12 Oxidation of [Pt(2)(5)]BF_4 with H_2O_2

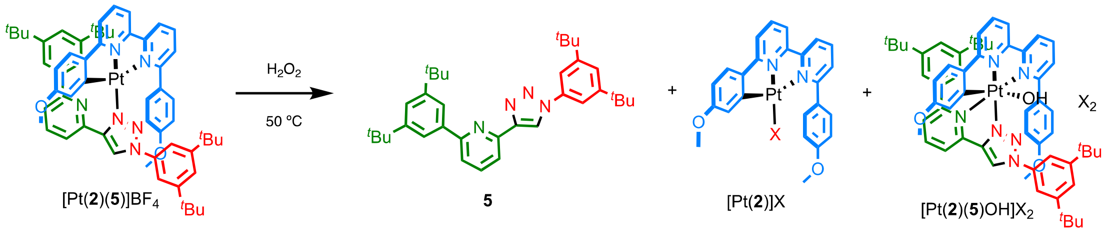

Scheme S12. The demonstration of oxidation of complex [Pt(**2**)(**5**)]BF_4 with H_2O_2

[Pt(**2**)(**5**)]BF_4 (14 mg, 0.012 mmol) was heated in H_2O_2 (30% w/w in H_2O, 2 mL) at 50 º. The aliquot samples (0.5 mL) were diluted with CH_2Cl_2 then washed with NH_4BF_4. The organic phase was dried over $MgSO_4$ and the solvent was removed *in vacuo*. The reaction mixture was then analysed by ^1H NMR and LR-MS.

^1H NMR (**Figure S58**) indicated complete consumption of [Pt(**2**)(**5**)]BF_4 after 3 h and formation of single major species. ESI-LRMS (m/z = 551.2) suggests this product is [Pt(**2**)(**5**)(OH)]X_2. Leaving the reaction overnight led to significant decomposition to produce axle **5**, suggesting the lability of such a Pt-triazole bond without the protection of mechanical bond.

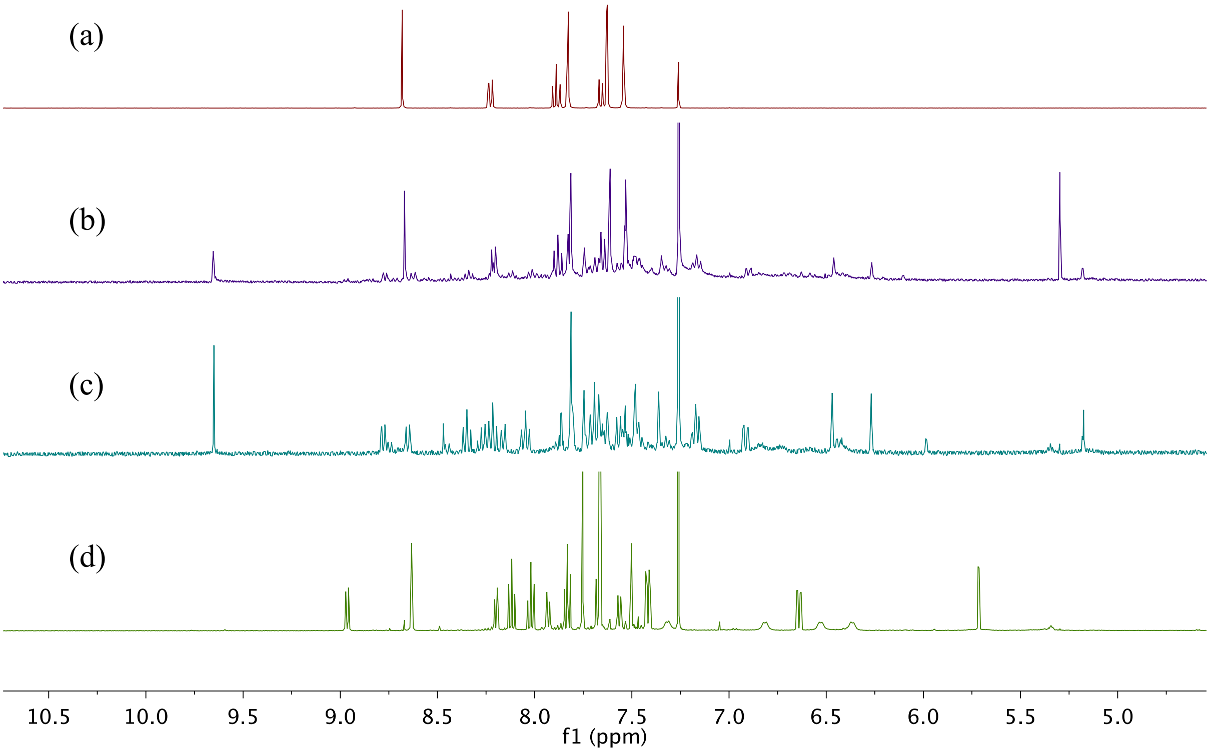

Figure S58. Partial ^1H NMR ($CDCl_3$, 400 MHz, 298 K) of (a) axle **5**; (b) product of reaction of [Pt(**2**)(**5**)]BF_4 with H_2O_2 for 20 h after NH_4BF_4 work up; (c) complex [Pt(**2**)(**5**)]BF_4 reacting with H_2O_2 after 3 h after NH_4BF_4 work up;(d) complex [Pt(**2**)(**5**)]BF_4

5 Photophysical data

5.1 Photophysical data for [Pt(1H)Cl$_2$(DMSO)]

Table S8. Photophysical data for [Pt(**1**H)Cl$_2$(DMSO)].

Absorption λ_{max} / nm (ε / M^{-1} cm^{-1})	258 (36500), 317sh (8680)
Emission λ_{max} / nm	383 (very weak)
$\Phi_{lum} \times 10^2$ [a]	--[e]
τ / ms [b]	--[e]
k_r / 10^3 s^{-1} [c]	--[e]
Σk_{nr} / 10^5 s^{-1} [c]	--[e]
Emission at 77K [d] — λ_{max} / nm	610
Emission at 77K [d] — τ / ms	140

[a] Luminescence quantum yield in deoxygenated solution, measured using [Ru(bpy)$_3$]Cl$_2$ (aq) as the standard. [b] Luminescence lifetime in deoxygenated solution; values in parenthesis are for air-equilibrated solution. [c] Radiative k_r and non-radiative Σk_{nr} rate constants estimated using the approximation that the emissive state is formed with unitary efficiency and thus k_r = F / t and Sk_{nr} = (1–F) / t. [d] Emission data at 77 K recorded in EPA (diethyl ether / isopentane / ethanol, 2:2:1 v/v). [e] No emission is observed for this complex in solution at room temperature.

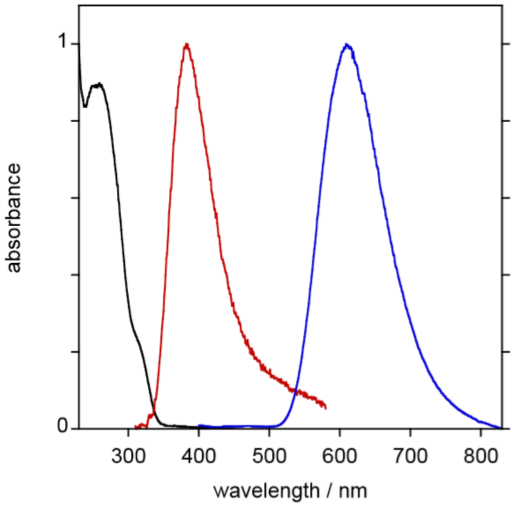

Figure S59. Absorption spectra in CH$_2$Cl$_2$ (black line) and emission spectra in CH$_2$Cl$_2$ at 298 K and in EPA at 77 K (red and blue lines respectively) for [Pt(**1**H)Cl$_2$(DMSO)].

5.2 Solid State Photophysical Data

The solid-state emission spectra and lifetimes were recorded for powdered samples held between quartz slides orientated at 54.7° from the horizontal. A 405 nm laser diode was used for excitation of the sample, and the emitted light was detected by the same system as that described above for luminescence lifetimes in solution. Quantum yields in the solid state were determined using an integrating sphere. Finely powdered samples were contained within Spectralon sample holders of 10 mm diameter. Powdered BaSO4 was used as a non-emissive blank. Scattered light at the excitation wavelength λex = 400 nm for sample and blank was measured using a neutral density filter of O.D. = 2, while the emission region was monitored in the absence of the filter.

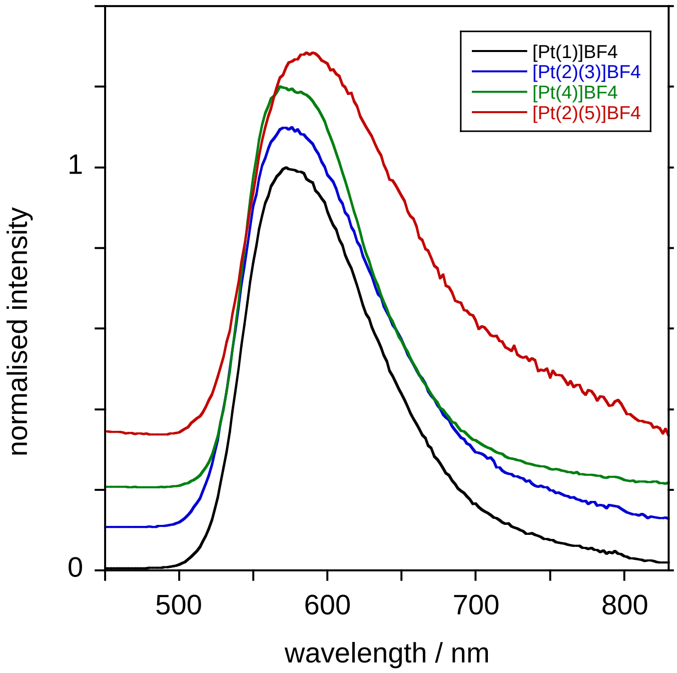

Figure S60 Spectra of the Pt(II) complexes in the solid state at 295 K. An offset of 0.1 units on the intensity axis has been applied the four different samples for clarity.

Table S9 Luminescence lifetimes of the Pt(II) complexes in the solid state at 295 K.

Complex	Luminescence lifetime μs[(a)]	Quantum yield × 10^2 [(b)]
[Pt(**1**)]BF_4	2.5	4.7
[Pt(**2**)(**3**)]BF_4	2.5	3.9
[Pt(k^4-**4**)]BF_4	2.6	6.7
[Pt(**2**)(**5**)]BF_4	1.6	4.5

(a) Estimated uncertainty in τ is ± 0.5 μs. *(b)* Estimated uncertainty in τ is ± 2 × 10^{-2}.

6 Effect of additives on [Pt(k^4-4)]BF_4

6.1 ^{1}H NMR spectra of complex [Pt(k^4-4)]BF_4 with 1 equivalent of H^+, Ag^I, Cu^I or Au^I

To investigate the binding of [Pt(k^4-**4**)]BF_4 with cations, the complex was combined with 1 equivalent TFA, $AgSbF_6$, Me_2SAuCl or $Cu(MeCN)_4PF_6$ and the solution stirred for 2 h at rt. The solvent was removed and the residue was redissolved in $CDCl_3$ for NMR analysis.

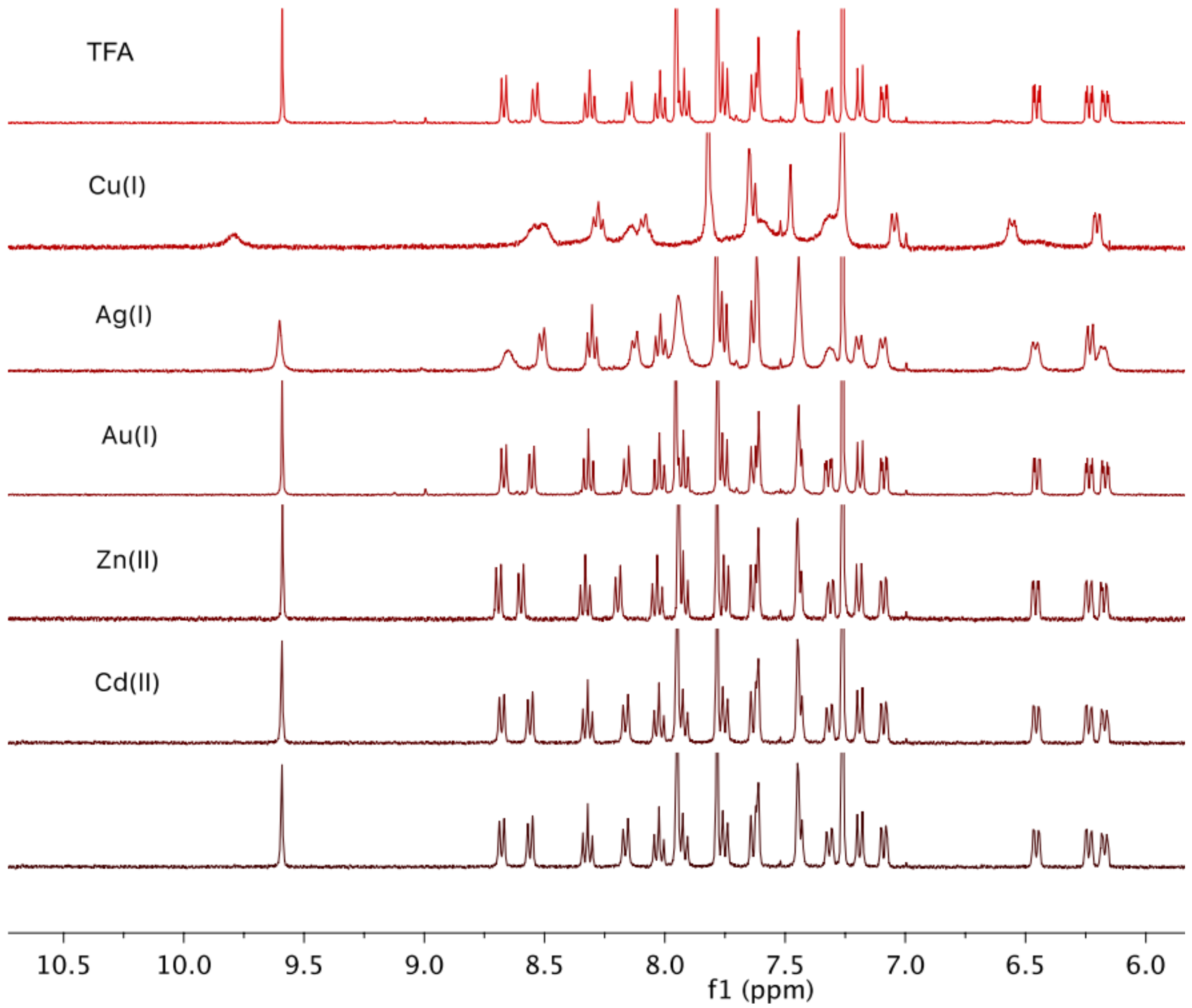

Figure S61 ^{1}H NMR of [Pt(k^4-**4**)]BF_4 (bottom) with 1 equivalent cations H^+ (TFA), Cu(I) ($Cu(MeCN)_4PF_6$), Ag(I) ($AgSbF_6$), Au(I) (Me_2SAuCl), Zn(II) ($Zn(ClO_4)_2$) or Cd(II) ($Cd(ClO_4)_2$) in $CDCl_3$ at 298K

6.2 ^{1}H NMR Titration of complex [Pt(k^4-4)]BF_4 with $AgSbF_6$ in CD_2Cl_2

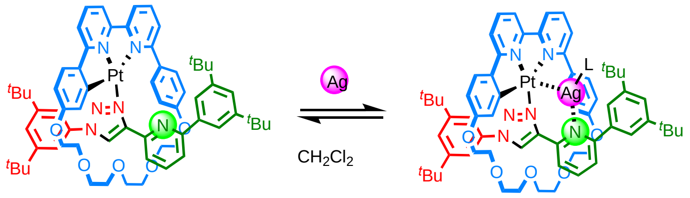

Scheme S13 Titration of $AgSbF_6$ to complex [Pt(k^4-**4**)]BF_4 at 298K

A qualitative ^{1}H NMR titration of complex [Pt(k^4-**4**)]BF_4 with $AgSbF_6$ in CD_2Cl_2 was carried out (**Figure S62**). The addition of the $AgSbF_6$ led to broadening and shifting of many signals associated with the starting complex. For example, axle triazole proton H_a moved to higher ppm while macrocycle proton H_A moved to lower ppm. Once 4 equivalents of $AgSbF_6$ had been added the signals resolved to reveal a new well defined complex.

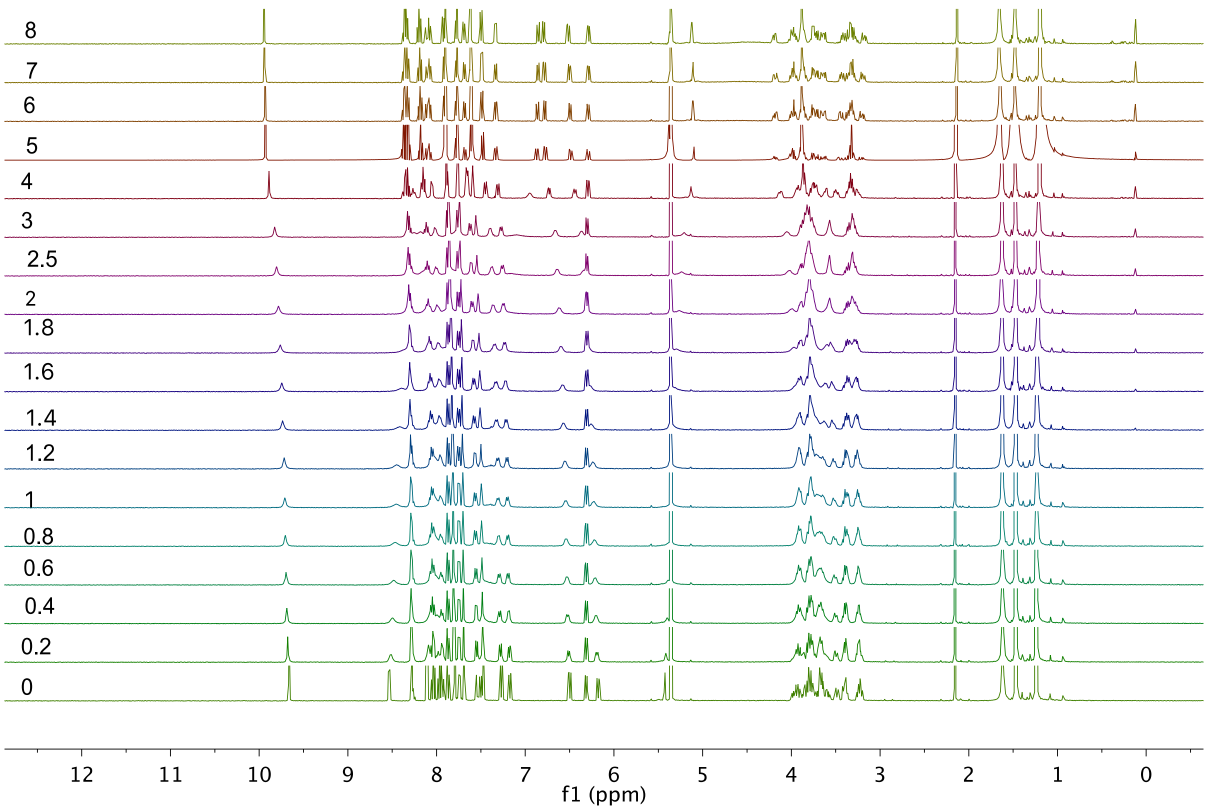

Figure S62 ^{1}H NMR titration of [Pt(k^4-**4**)]BF_4 with $AgSbF_6$ in CD_2Cl_2 (C = 0.011 M)

The non-linear fitting of the NMR change at 6.55 ppm against the equivalent of Ag(I) gave K_a as 13.1 M based on a 1:1 binding model (**Figure S63**). However, as $AgSbF_6$ is not freely soluble in CD_2Cl_2 the binding curves obtained are not accurate. The results are reported here for completeness but the binding constant obtained and the associated error are largely meaningless.

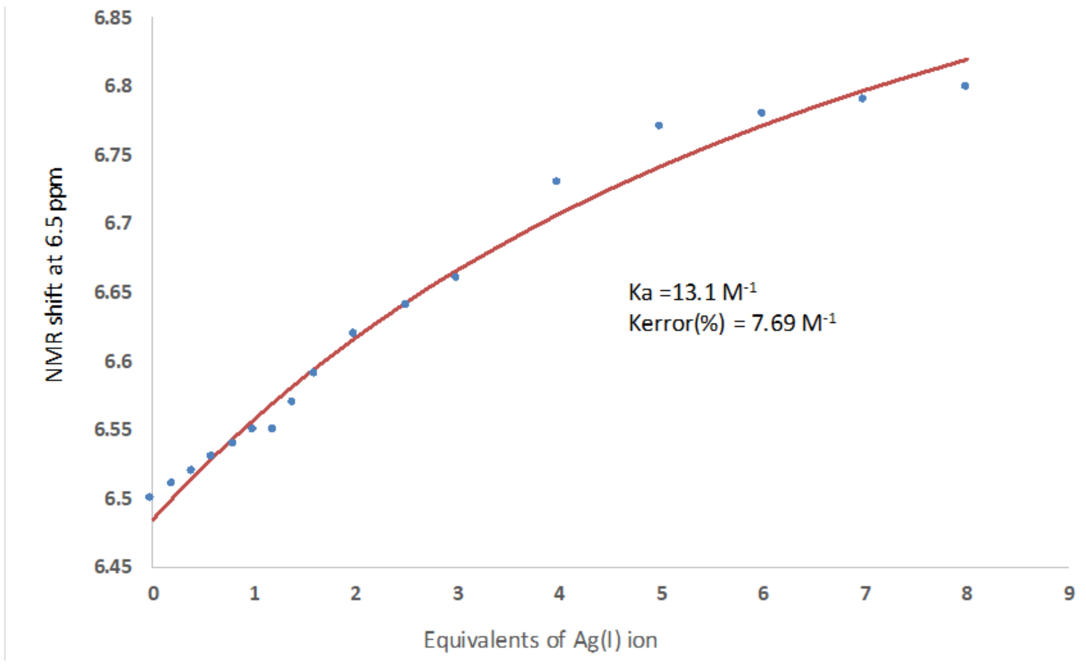

Figure S63 Binding isotherm of [Pt(k4-**4**)]BF_4 with $AgSbF_6$ in (CD_2Cl_2 C = 0.011 M)

6.3 UV-Vis titration of complex [Pt(k^4-4)]BF_4 with $AgSbF_6$

UV-vis titration of [Pt(k^4-**4**)]BF_4 with $AgSbF_6$ (0-3 equivalents) revealed that the band at 368 nm gradually decreases, while the absorption bands at 325 and 387-500 nm increase. Well-defined isosbestic points at 352 and 387 nm suggest the distribution of two species during the titration. Non-linear fitting of the absorption changes against the equivalent of cation gave K_a as 2.4×10^4 M based on a 1:1 binding model, although it should be noted that the curve fit has rather a large error, possibly due to the poor solubility of $AgSbF_6$ even at these lower concentrations. Job plot analysis supports a 1 : 1 binding mode (see 6.5) but it should be noted that N^2 of the triazole may act as a second binding site, allowing the formation of M_2L species, which complicates the analysis.

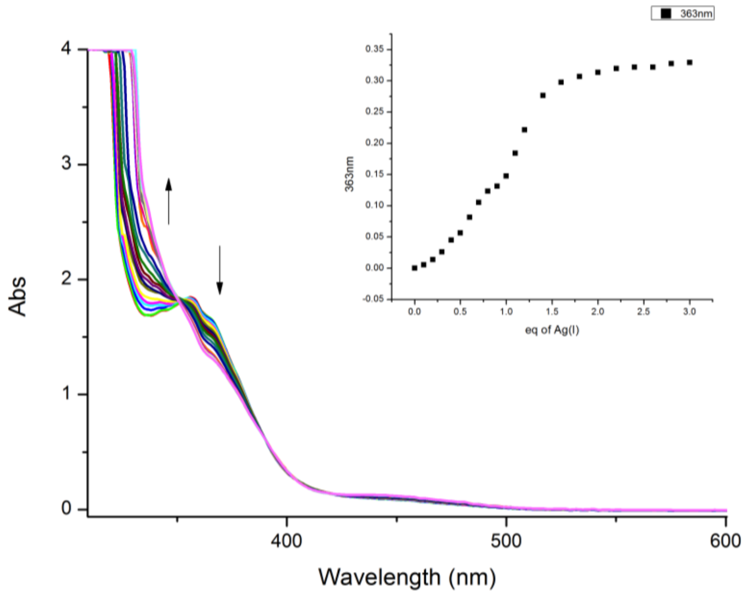

Figure S64. UV-Vis titration of [Pt(**4**)BF_4] with $AgSbF_6$ in CH_2Cl_2 C = 3 x 10^{-5} M

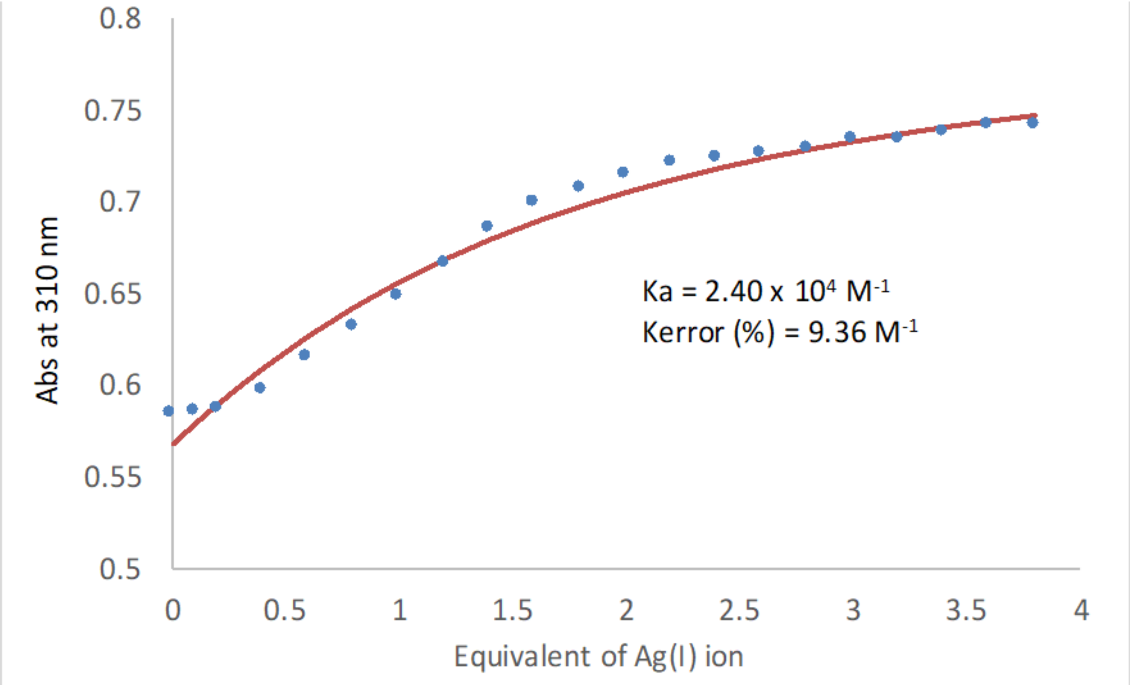

Figure S65 Non-linear fit of the titration of [Pt(**4**)BF$_4$] with AgSbF$_6$ in CH$_2$Cl$_2$ C = 3 x 10^{-5} M

6.4 Emission titrations of complex [Pt(k^4-4)]BF$_4$ with AgSbF$_6$

The intensity of the emission band at 570 nm increases gradually with progressive addition of Ag(I) (0-3.5 equivalents). The non-linear fitting of the emission changes at 565 nm against the equivalent of cation gave K_a as 7.5 × 10^3 M based on a 1:1 binding model (**Figure S72**), albeit with a relatively poor goodness of fit (see comments above).

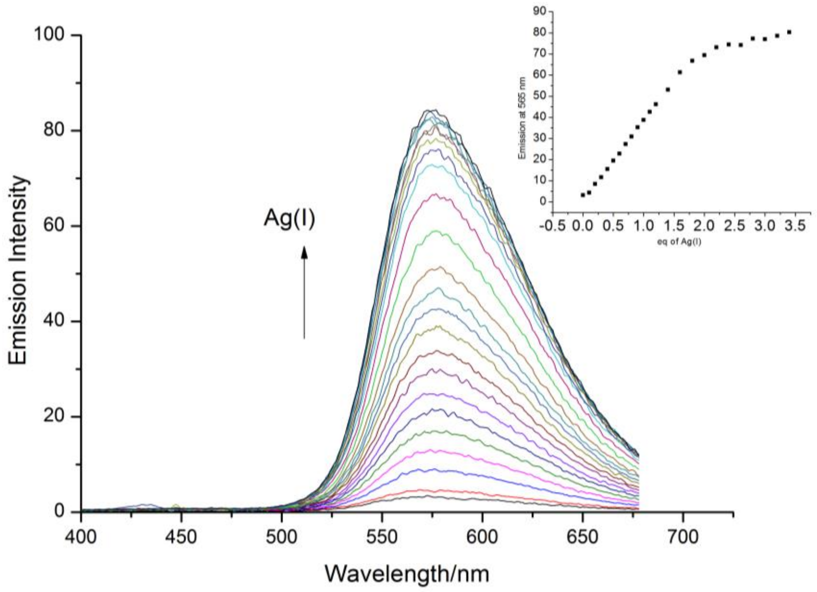

Figure S66 Emission titration of [Pt(k^4-**4**)]BF$_4$ with AgSbF$_6$ in CH$_2$Cl$_2$ C = 3 x 10^{-5} M

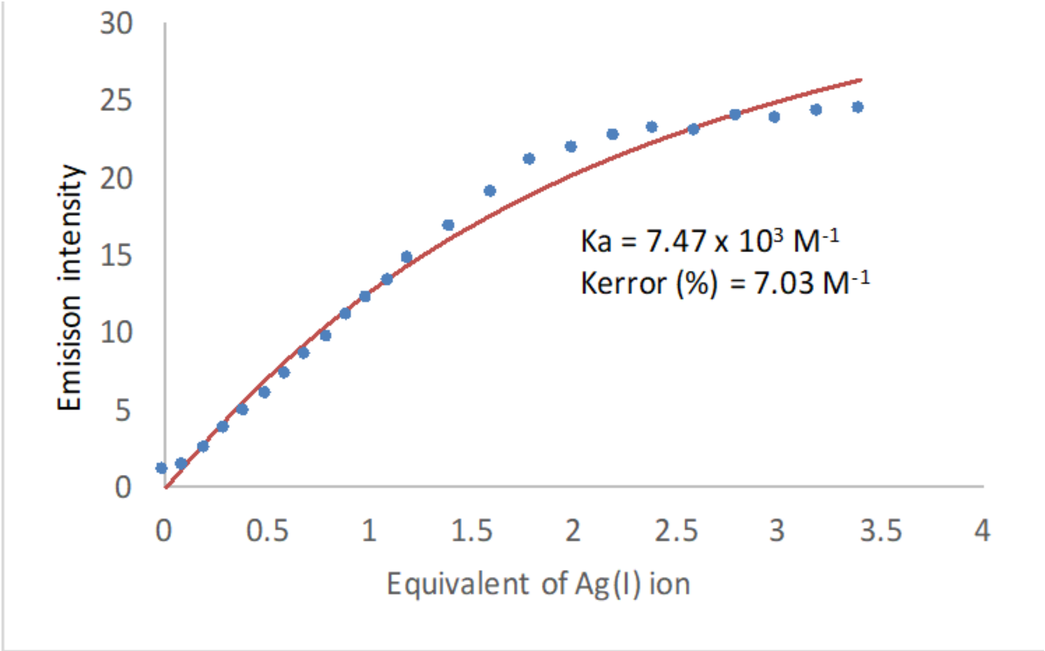

Figure S67 Non-linear fit of the emission titration of [Pt(k[4]-**4**)]BF_4 with $AgSbF_6$ in CH_2Cl_2 C = 3 x 10^{-5} M

6.5 Job Plot of complex [Pt(k^4-4)]BF_4 with $AgSbF_6$

A Job plot was conducted by UV-Vis and found to support the proposed 1 : 1 binding model.

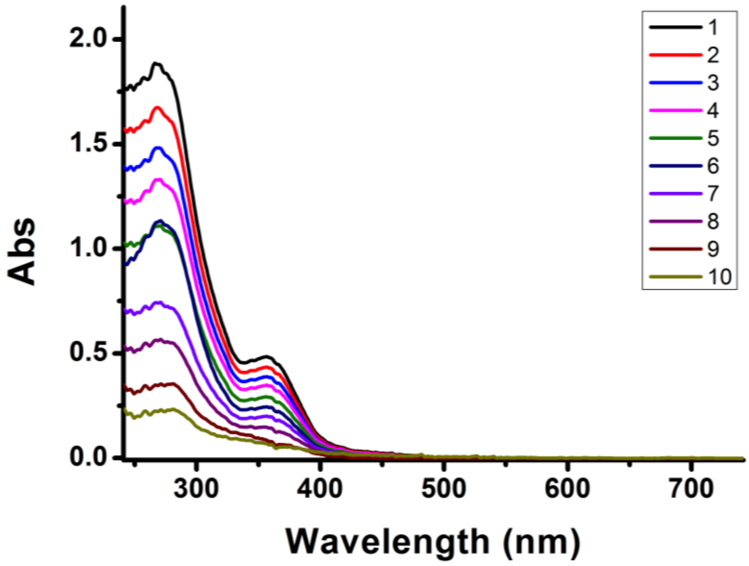

Figure S68. Job plot of [Pt(k^4-**4**)]BF_4 with $AgSbF_6$ in CH_2Cl_2 total C = 3 x 10^{-5} M

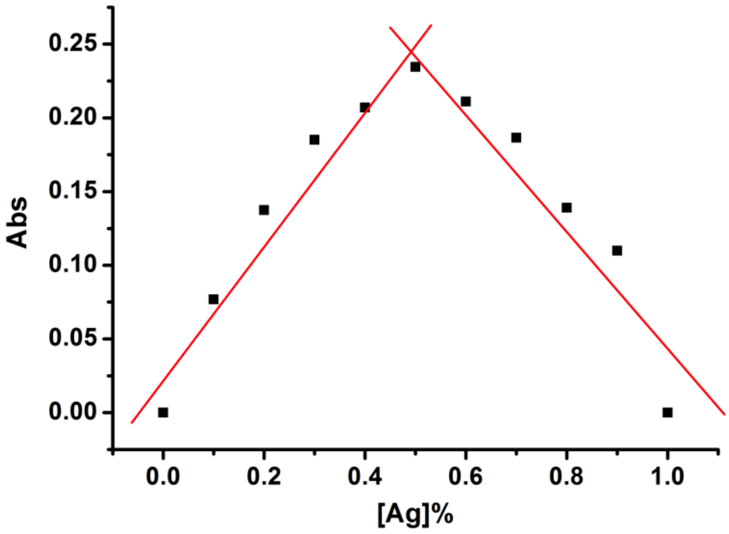

Figure S69. Job plot of [Pt(k^4-**4**)]BF_4 with $AgSbF_6$ in CH_2Cl_2 total C = 3 x 10^{-5} M

6.6 Titration of [Pt(k^4-4)]BF_4 with Cu(I)

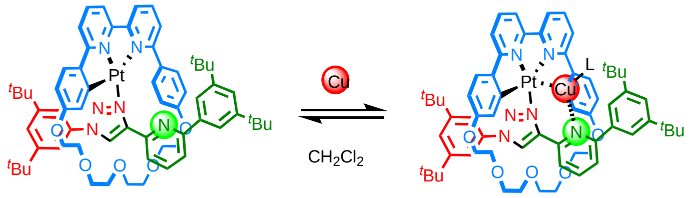

Scheme S14 Titration of $Cu(MeCN)_4PF_6$ to complex [Pt(k^4-4)]BF_4 at 298 K

During the titration of [Pt(k^4-**4**)]BF_4 with incremental amounts of [$Cu(MeCN)_4$]PF_6 (0-6 equivalents), the bands at 270 and 368 nm gradually decrease, while the absorption at bands at 238, 325 and 387-500 nm increase with well-defined isosbestic points at 270, 352 and 387 nm, indicating the distribution of two species during the titration. The non-linear fitting of the absorption change against the equivalent of cation gave K_d as 2.4×10^{-6} based on a 1:1 binding model.

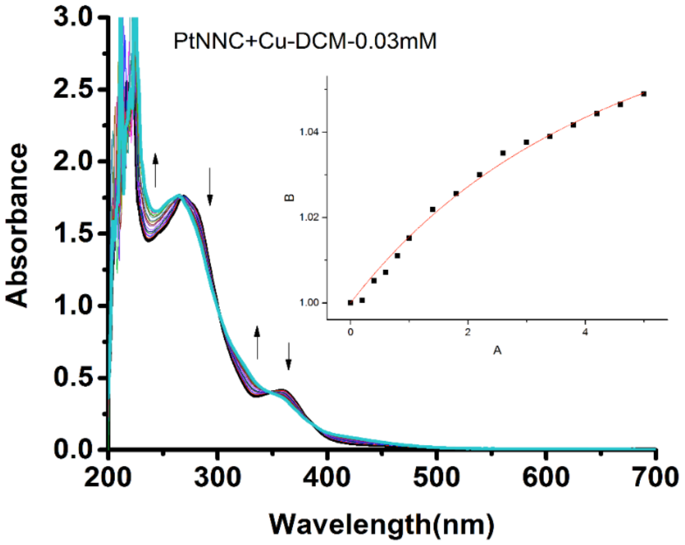

Figure S70. UV-Vis spectral changes of [Pt(k^4-**4**)]BF_4 (3×10^{-5} M) in CH_2Cl_2, upon addition of [Cu(MeCN)$_4$]PF_6, insert: A plot of the absorbance change at 353 nm as a function of the equivalents of [Cu(MeCN)$_4$]PF_6 and its nonlinear fit to a 1:1 binding model.

The titration of Cu(I) to [Pt(k^4-**4**)]BF_4 resulted in negligible change in the emission spectra. Upon the addition of Cu(I), the weak emission band at 578 nm gradually decreased and stopped changing after 0.5 equivalent Cu(I) addition. Although the emission at range 400-500 nm underwent a slight increase, considering the initial Φ_{lum} is only 0.12%, these changes are negligible.

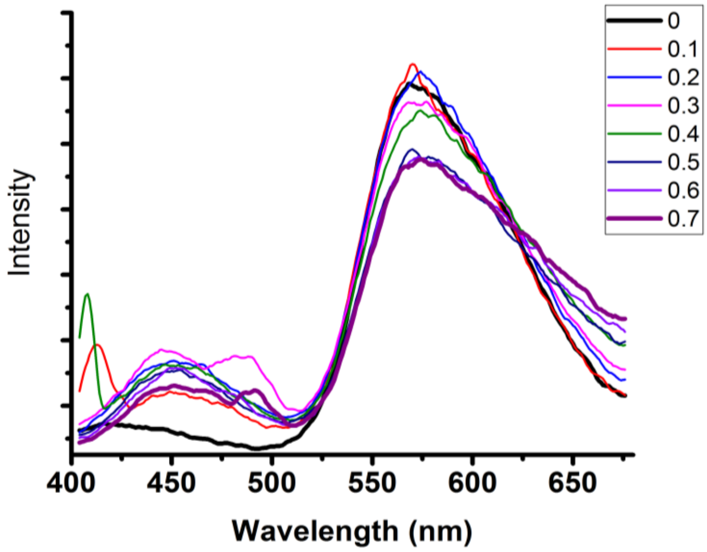

Figure 71. Emissive spectral changes of [Pt(k^4-**4**)]BF_4 (3×10^{-5} M) in CH_2Cl_2, upon addition of [Cu(MeCN)$_4$]PF_6.

6.7 Titration of [Pt(k4-4)]BF_4 with [Au(Cl)(Me_2S)]

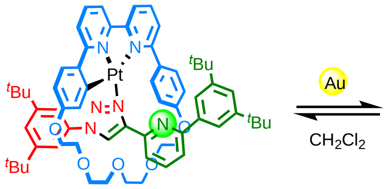

Scheme S15 Titration of Au(SMe_2)Cl to complex [Pt(k4-**4**)]BF_4 at 298 K

Titration of [Pt(k^4-**4**)]BF_4 with [Au(Cl)(Me_2S)] resulted in the absorption band at 274 and 352 nm gradually decreasing while the absorption in 230-250 nm range increases with one well-defined isosbestic point at 248 nm. As shown in **Figure S72**, the change in absorption against the equivalents of Au(I) shows a linear trend and failed to reach saturation, which attributed to very weak binding of [Pt(k4-**4**)]BF_4 with Au(I), and, unfortunately, we were not able to determine the binding constant.

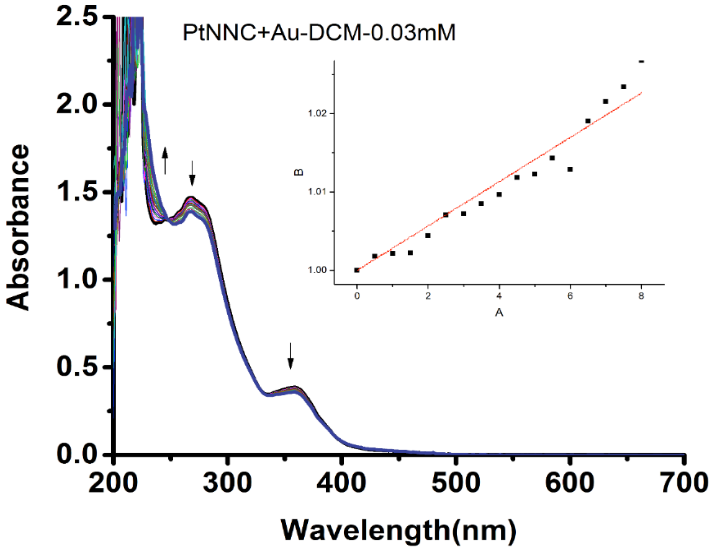

Figure S72. UV-Vis spectral changes of [Pt(k4-4)]BF_4 (3×10^{-5} M) in CH_2Cl_2, upon addition of Au(I), insert: A plot of the absorbance change at 353 nm as a function of the equivalent of Au(I) and its theoretical fit for the 1:1 binding of [Pt(k4-4)]BF_4 with Au(I).

7 Titration of non-interlocked Pt complex [Pt(2)(5)]BF_4 with Ag(I)

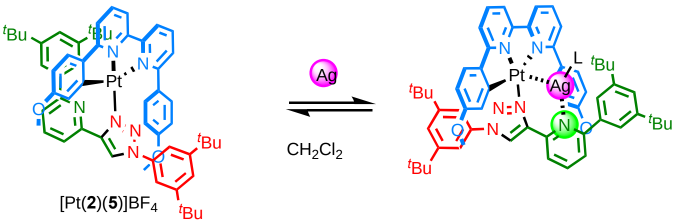

Scheme S16. The titration of complex [Pt(**2**)(**5**)]BF_4 to $AgSbF_6$ in CH_2Cl_2

7.1 UV-vis titrations of complex [Pt(2)(5)]BF_4 with $AgSbF_6$

UV-vis titration of [Pt(**2**)(**5**)]BF_4 with $AgSbF_6$ (0-3 equivalents) revealed that the band at 368 nm gradually decreases, while the absorption bands at 325 and 387-500 nm increase (**Figure S73**). Well-defined isosbestic points at 352 and 387 nm indicate the distribution of two species during the titration.

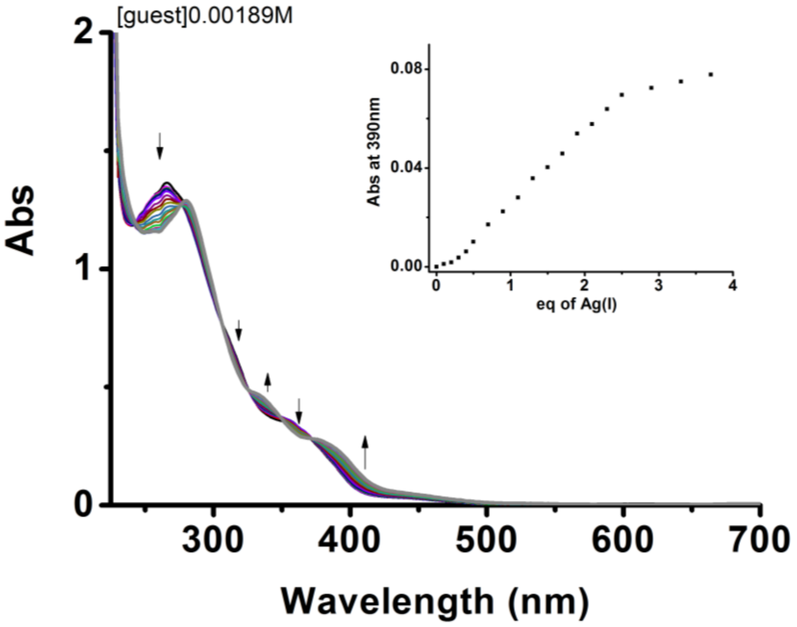

Figure S73. UV-Vis spectral changes of [Pt(**2**)(**5**)]BF_4 (1.5×10^{-5} M) in CH_2Cl_2, upon addition of $AgSbF_6$ (1.9×10^{35} M) , insert: A plot of the absorbance change at 390 nm as a function of the equivalent of $AgSbF_6$.

The intensity of the emission band at 570 nm increases gradually with progressive addition of Ag(I) (0-3.5 equivalents). The non-linear fitting of the emission changes at 565 nm against the equivalent of cation gave K_a as 7.5×10^4 M based on a 1:1 binding model (**Figure S74**).

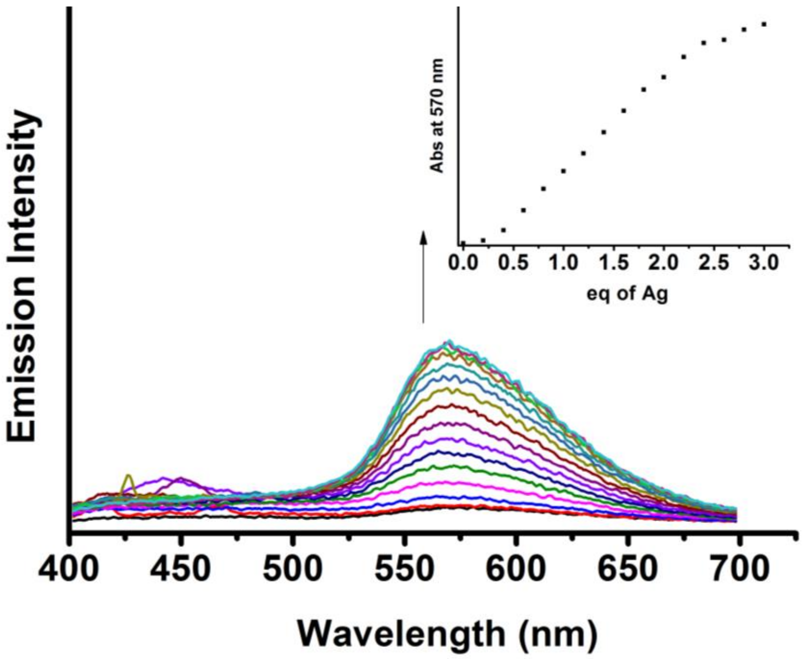

Figure S74. Emissive spectral changes of [Pt(**2**)(**5**)]BF_4 (1.5 × 10^{-5} M) in CH_2Cl_2, upon addition of $AgSbF_6$, insert: A plot of the emission change at 570 nm as a function of the equivalent of $AgSbF_6$

8 Addition of Δ-trisphat tetrabutylammonium salt to [Pt(1)]BF_4

Combining [Pt(**1**)]BF_4 with Δ-trisphat tetrabutylammonium salt resulted in the splitting of some of the ^{1}H NMR signals corresponding to the Pt complex into diasterotopic pairs, consistent with the mechanically planar chiral nature of these Pt complexes (**Figure S75**).

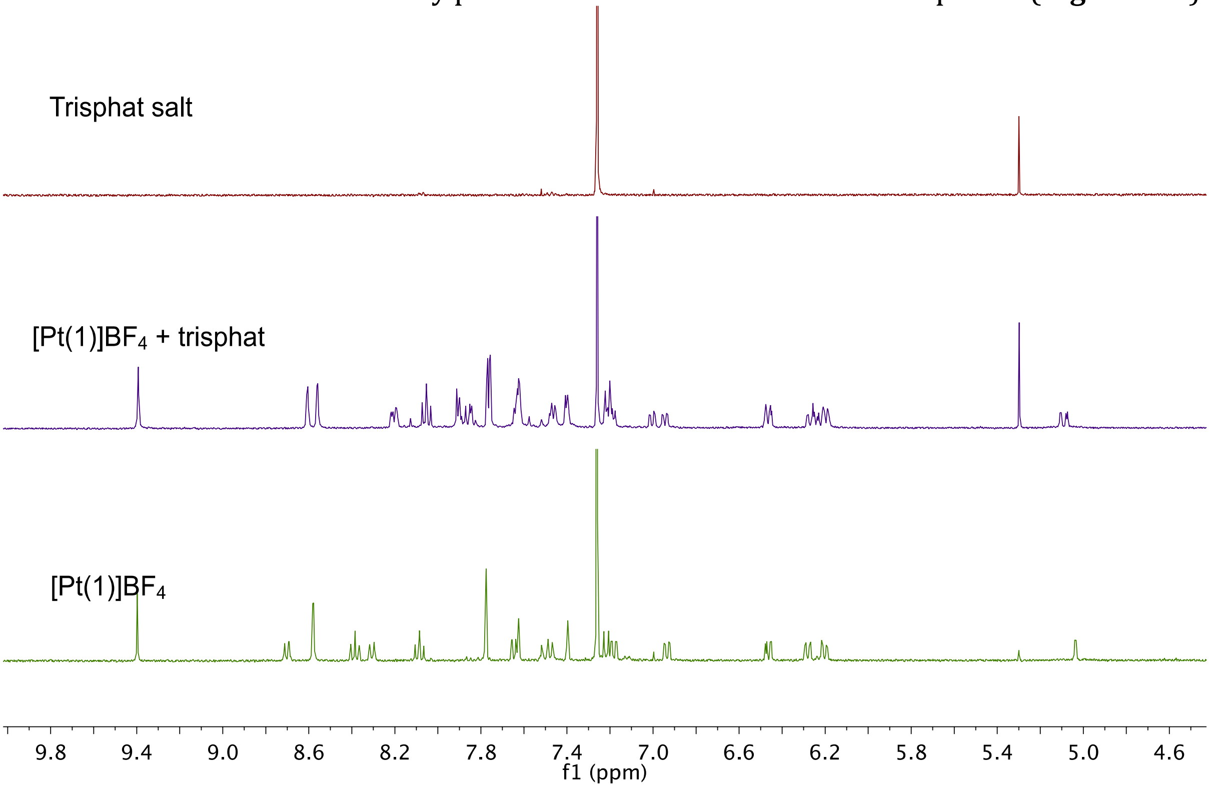

Figure S75. Comparisons of partial ^{1}H NMR of [Pt(**1**)]BF_4 and [Pt(**1**)]BF_4 with 1 equivalent trisphat tetrabutylammonium salt.

9 References

1 A. Pigorsch and M. Köckerling, *Cryst. Growth Des.* 2016, **16**, 4240–4246.
2 G. T. Potter, G. C. Jayson, G. J. Miller and J. M. Gardiner, *J. Org. Chem.* 2016, **81**, 3443–3446.
3 K. Nakamaru, *Bull. Chem. Soc. Japan* 1981, **55**, 2697.
4 J. E. M. Lewis, R. J. Bordoli, M. Denis, C. J. Fletcher, M. Galli, E. A. Neal, E. M. Rochette and S. M. Goldup, *Chem. Sci.*, 2016, **7**, 3154–3161
5 M. Cirulli, A. Kaur, J. E. M. Lewis, Z. Zhang, J. A. Kitchen, S. M. Goldup and M. M. Roessler, *J. Am. Chem. Soc.*, 2019, **141**, 879–889
6 R. S. Stoll, M. V. Peters, A. Kuhn, S. Heiles, R. Goddard, M. Bühl, C. M. Thiele and S. Hecht, *J. Am. Chem. Soc.* 2009, **131**, 357–367.
7 W. Zhu and D. Ma, *Chem. Commun.* 2004, 888.
8 R. J. Bordoli and S. M. Goldup, *J. Am. Chem. Soc.*, 2014, **136**, 4817–4820
9 O. V. Dolomanov, L. J. Bourhis, R. J. Gildea, J. A. K. Howard and H. Puschmann, *J. Appl. Crystallogr.*, 2009, **42**, 339–341
10 G. M. Sheldrick, *Acta Crystallogr. Sect. A Found. Adv.*, 2015, **71**, 3–8
11 G. M. Sheldrick, *Acta Crystallogr. Sect. C Struct. Chem.*, 2015, **71**, 3–8
12 L. J. Bourhis, O. V. Dolomanov, R. J. Gildea, J. A. K. Howard and H. Puschmann, *Acta Crystallogr. Sect. A Found. Adv.*, 2015, **71**, 59–75
